# Direct Catalytic Asymmetric Doubly Vinylogous Michael Addition of α,β-Unsaturated γ-Butyrolactams to Dienones**

**DOI:** 10.1002/anie.201504276

**Published:** 2015-07-15

**Authors:** Xiaodong Gu, Tingting Guo, Yuanyuan Dai, Allegra Franchino, Jie Fei, Chuncheng Zou, Darren J Dixon, Jinxing Ye

**Affiliations:** Engineering Research Centre of Pharmaceutical Process Chemistry, Ministry of Education, Shanghai Key Laboratory of New Drug Design, School of Pharmacy East China University of Science and Technology, 130 Meilong Road, Shanghai 200237 (China); Department of Chemistry, Chemistry Research Laboratory University of Oxford, 12 Mansfield Road, Oxford OX1 3TA (UK)

**Keywords:** asymmetric catalysis, conjugation, cyclizations, nucleophilic addition, organocatalysis

## Abstract

An asymmetric doubly vinylogous Michael addition (DVMA) of α,β-unsaturated γ-butyrolactams to sterically congested β-substituted cyclic dienones with high site-, diastereo-, and enantioselectivity has been achieved. An unprecedented DVMA/vinylogous Michael addition/isomerization cascade reaction affords chiral fused tricyclic γ-lactams with four newly formed stereocenters.

Remote stereocontrol in catalytic asymmetric reactions is a major challenge in modern organic synthesis.^1^, ^2^ Recently, asymmetric organocatalysis has been successfully applied to the functionalization of unsaturated carbonyl compounds at their γ-, δ-, and ε-positions with high stereo- and site-selectivity.^1^ The two basic activation strategies exploit LUMO-lowering and HOMO-raising effects, whereby iminium ions and either di- or trienamines are formed by condensation of the carbonyl substrates with the amine function of chiral organocatalysts.^1^, ^3^ Melchiorre and co-workers achieved the δ-functionalization of enones by using a cinchona-based primary amine, which forms an iminium ion with the polyunsaturated carbonyl substrate, thus delivering the LUMO-lowering effect through the conjugated π system.^4^ Enamine catalysis^5^ was also successfully applied to vinylogous systems. Di- and trienamine catalysis,5b,c usually employing chiral secondary amines to activate the γ and ε sites of unsaturated aldehydes, has led for instance to a series of Diels–Alder cycloadditions5e and other remote functionalization reactions.^6^

In most studies a single vinylogous substrate, either the electrophilic or nucleophilic partner, was used.^1^, ^3^–^7^ In 2013 Jørgensen and co-workers reported the first organocatalytic doubly vinylogous Michael-type reaction, namely the 1,6-addition of alkylidene lactones to 2,4-dienals with the formation of a new stereocenter (Scheme 1a).^8^ It is significantly difficult to simultaneously activate the two vinylogous partners at their remote reactive sites whilst achieving high regio-, diastereo-, and enantiocontrol. Indeed, to the best of our knowledge there are no precedents for the catalytic, asymmetric doubly vinylogous Michael addition (DVMA) to 2,4-dienones, the much less reactive analogues of 2,4-dienals. The realization of such a reaction would prove the broad applicability of the organocatalytic vinylogous activation patterns, thus representing a significant advance in the field. Moreover, asymmetric doubly vinylogous reactions naturally leave two unsaturated C–C bonds in the product and provide a potential opportunity for additional transformations for the construction of complex chiral molecules.

**scheme 1:**
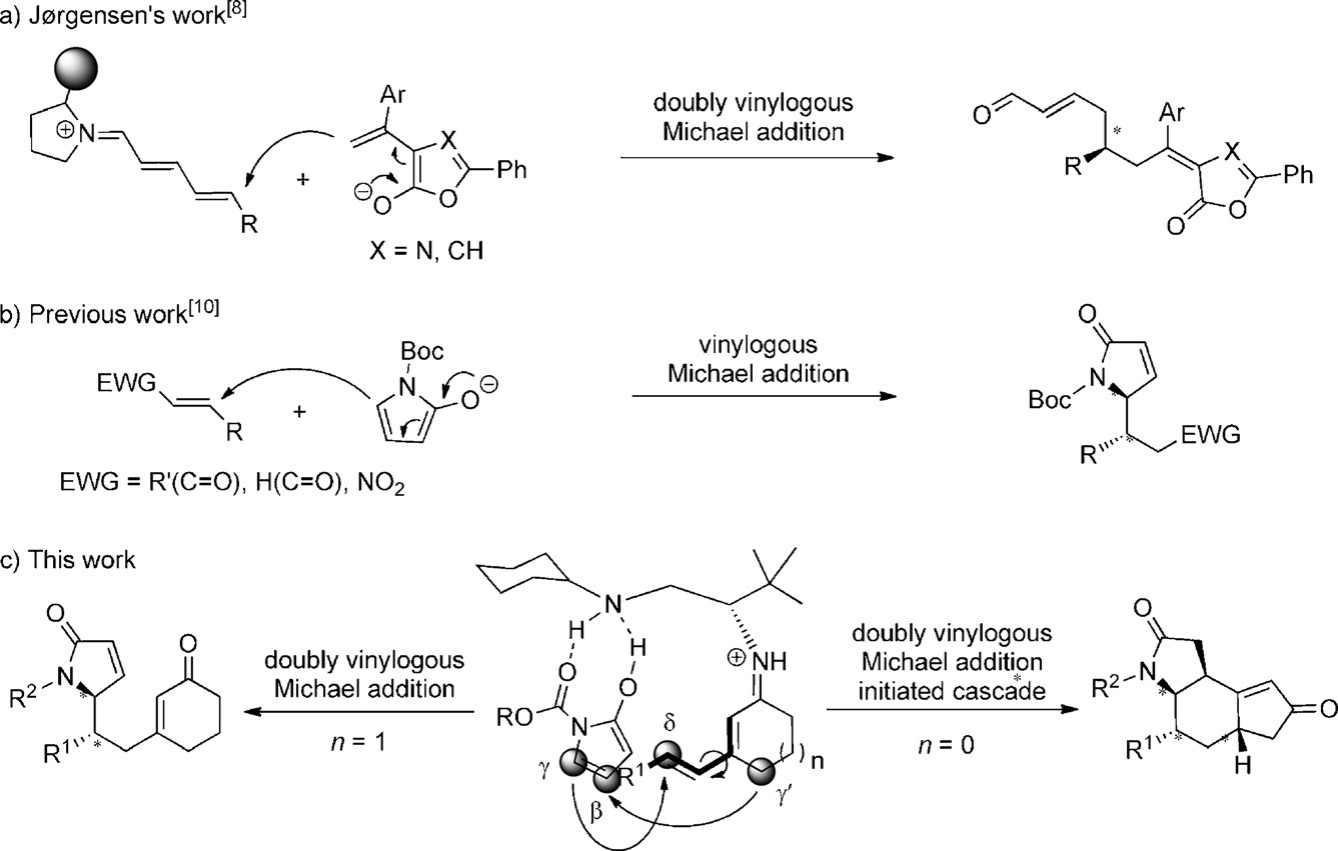
a) First asymmetric organocatalytic doubly vinylogous Michael addition. b) Use of α,β-unsaturated γ-butyrolactams in vinylogous Michael additions. c) This work: unprecedented asymmetric organocatalytic DVMA and a related cascade between α,β-unsaturated γ-butyrolactams and dienones. Boc=*tert*-butoxycarbonyl.

Herein we report the first doubly vinylogous Michael addition to 2,4-dienones by using a diamine derived from *tert*-leucine as an organocatalyst (Scheme 1c).^9^ The challenging γ to δ 1,6-addition reaction of N-protected α,β-unsaturated γ-butyrolactams^10^ to sterically congested β-substituted cyclic dienones proceeds with high regio- and stereoselectivity wherein strong hydrogen-bonding interactions between the N-protected, deprotonated butyrolactam and the catalyst are believed to be responsible for the observed control. In addition we report that by using 3-alkenyl cyclopent-2-enones as substrates, the initial DVMA is followed by a vinylogous Michael addition/isomerization cascade, thus affording tricyclic γ-lactams with four new stereocenters.

Our investigations began with a screen of a set of chiral diamines (**3 a–d**) in the DVMA of the dienone **1 a** and the N-protected α,β-unsaturated γ-butyrolactam **2 a** as shown in Table 1. The l-*tert*-leucine derivative **3 d** afforded the desired product **4 a** with encouraging conversion and good stereoselectivity (entry 4). This catalyst performed well in most solvents, but provided the best *ee* values in chlorinated solvents (89–91 % *ee*, entries 4–6), compared to the 85 % *ee* obtained with ethers and the less than 80 % *ee* obtained with polar solvents (see Table S1 in the Supporting Information). An extensive screening of acidic additives (see Table S2 in the Supporting Information) allowed identification of *p*-anisic acid as ideal. By using 20 mol % of *p*-anisic acid in CH_2_Cl_2_ at 4 °C, the product was obtained with 19:1 d.r. and 91 % *ee* (Table 1, entry 13; see Tables S3–S6 in the Supporting Information for full optimization studies). Finally, by increasing the amount of *p*-anisic acid to 40 mol % and adjusting the dienone/butyrolactam ratio to 2:1, the d.r. and *ee* values were slightly increased (entry 14). When the reaction was run under these optimized reaction conditions for 60 hours, the desired product was obtained with 95 % yield upon isolation, in greater than 19:1 d.r. and 91 % *ee* (see Table 2).

**Table 1:**
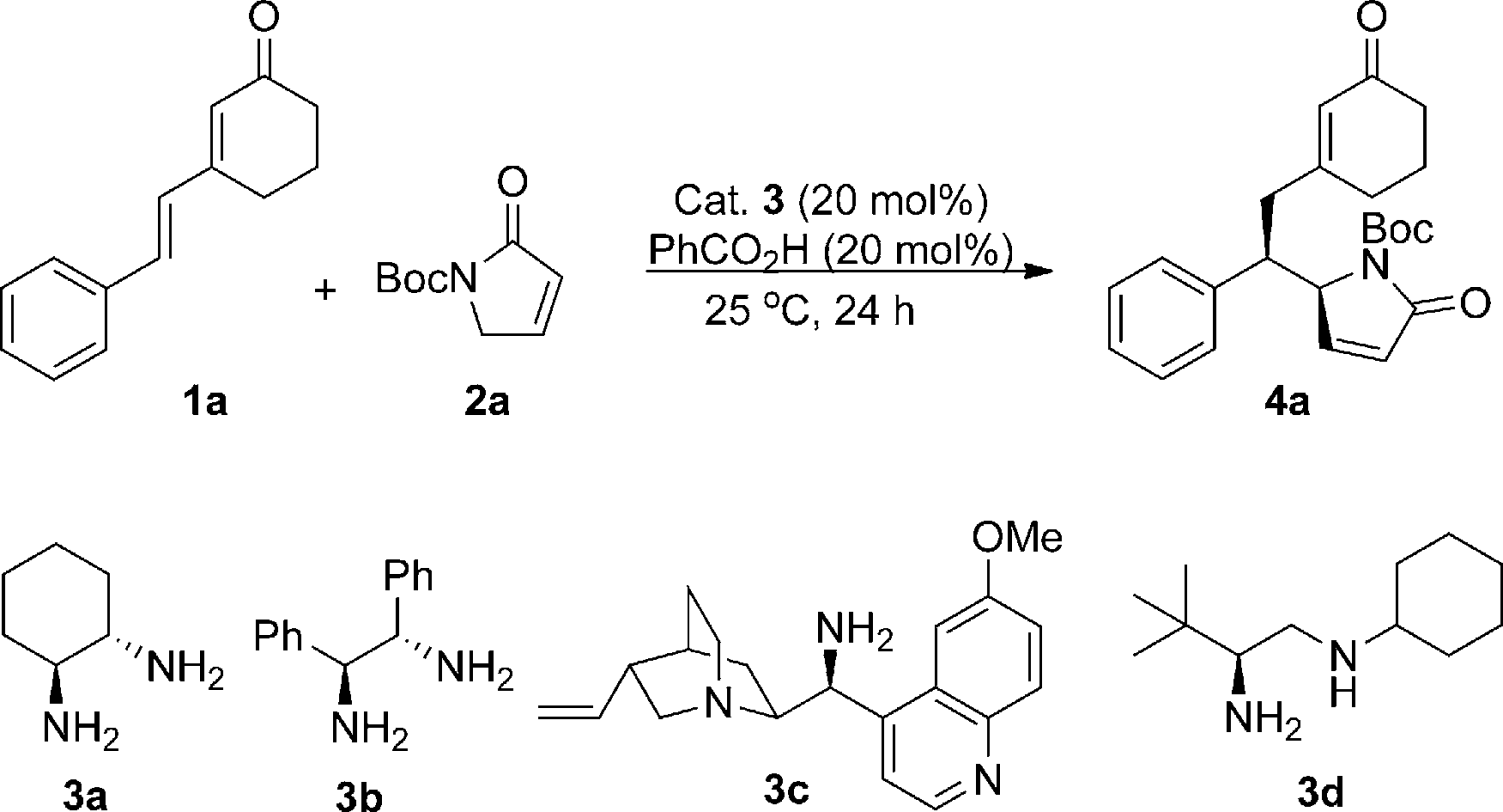
Catalyst screening and optimization of the doubly vinylogous Michael addition (DVMA) between 1 a and 2 a.[a]

Entry	Cat.	Solvent	Conv. [%][b]	d.r.[b]	*ee* [%][c]
1	**3 a**	CDCl_3_	16	4:1	−7
2	**3 b**	CDCl_3_	trace	n.d.	n.d.
3	**3 c**	CDCl_3_	trace	n.d.	n.d.
4	**3 d**	CDCl_3_	55	7:1	89
5	**3 d**	CH_2_Cl_2_	74	19:1	90
6	**3 d**	1,2-DCE	88	12:1	91
7	**3 d**	toluene	96	9:1	79
8	**3 d**	MTBE	95	7:1	82
9	**3 d**	EtOAc	86	6:1	86
10	**3 d**	*i-*PrOH	65	2:1	79
11[d,e]	**3 d**	1,2-DCE	86	16:1	91
12[d]	**3 d**	CH_2_Cl_2_	91	19:1	89
13[d,e]	**3 d**	CH_2_Cl_2_	76	19:1	91
14[e,f]	**3 d**	CH_2_Cl_2_	88	>19:1	92

[a] Reactions performed using 1.0 equiv of **2 a** (0.15 mmol, 0.5 m), 1.5 equiv of **1 a**, 0.2 equiv of catalyst **3**, and 0.2 equiv of PhCO_2_H at 25 °C for 24 h, unless otherwise stated.

[b] Conversion and d.r. values determined by ^1^H NMR analysis of the crude reaction mixture.

[c] Determined by HPLC analysis using a chiral stationary phase.

[d] With 0.2 equiv of *p*-anisic acid.

[e] Reaction performed at 4 °C for 48 h.

[f] With 0.4 equiv of *p*-anisic acid and 2.0 equiv of **1 a**. 1,2-DCE=1,2-dichloroethane, MTBE=methyl *tert*-butyl ether.

Next, the scope of the asymmetric DVMA with respect to dienone reaction partners was explored. By using the N-Boc-protected γ-butyrolactam **2 a** as the reacting partner, an extensive range of 3-alkenyl cyclohex-2-enones were transformed into the desired products **4** with good to excellent stereoselectivity (Table 2). Aryl-substituted dienones with electron-donating substituents in the *para*- and *meta*-positions of the aromatic ring gave excellent enantioselectivities and diastereoselectivities (**4 b**,**c**,**f**), whilst substrates with substituents in the *ortho*-position resulted in a slightly diminished *ee* value (**4 d**,**e**). The enantioselectivity remained excellent when the aryl ring bore electron-withdrawing and halogen substituents (**4 g**–**j**), although in the presence of the nitro group the d.r. value was reduced. Also, less reactive aliphatic substituted dienones (**4 k**,**l**) and bulky substrates with gem-dimethyl groups on the cyclohexenone (**4 m**,**n**) were well-tolerated. The absolute configuration of **4 p** was determined by X-ray crystallographic analysis.^11^

**Table 2:** Scope of the DVMA with respect to the dienones.[a]

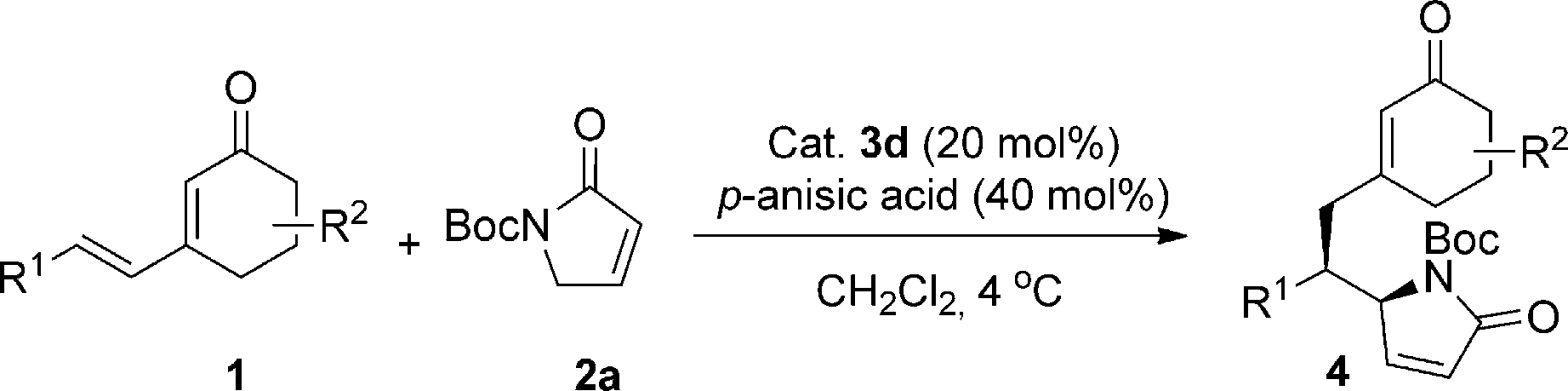
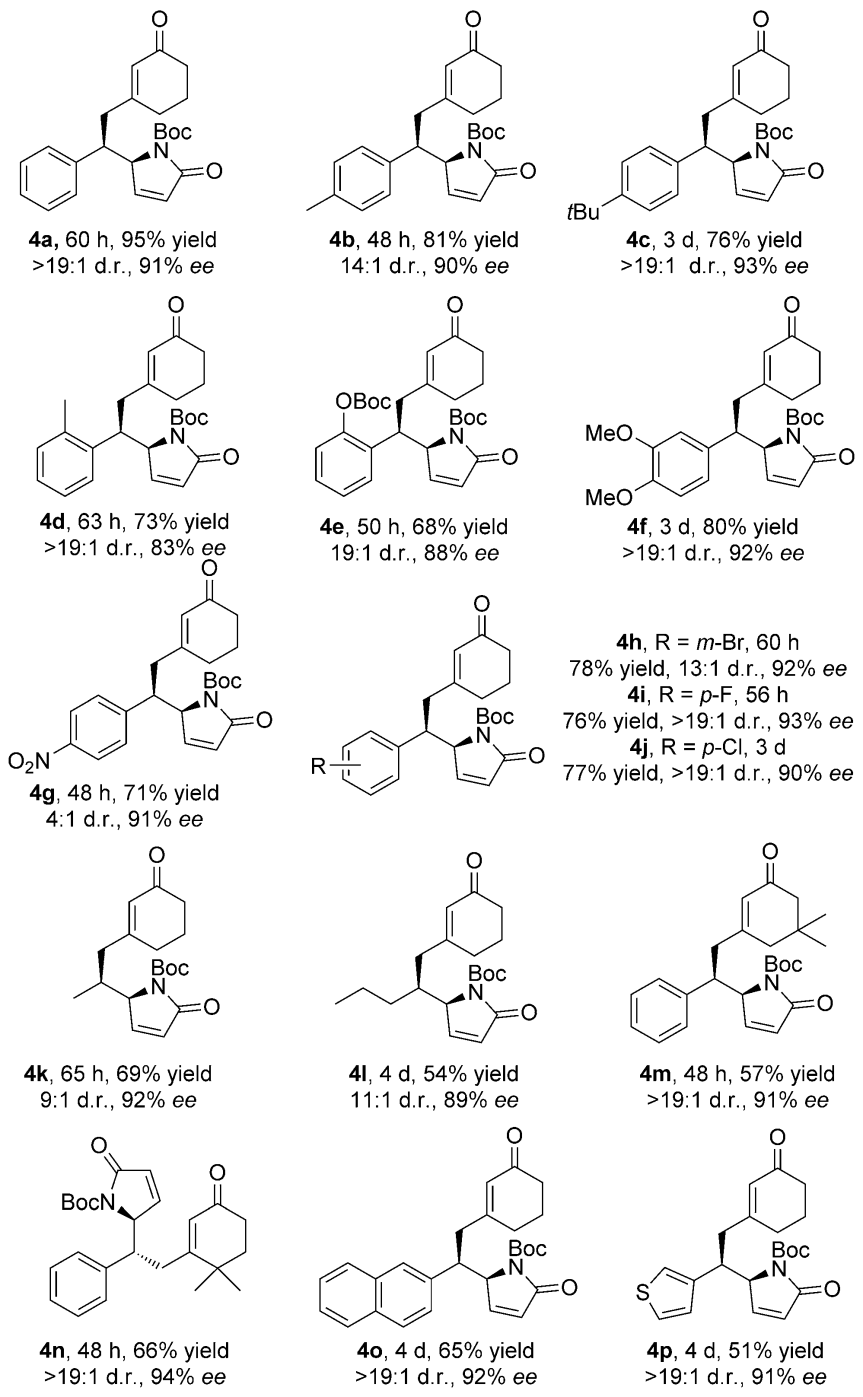

[a] Reactions performed using 1.0 equiv of **2** (0.2 mmol, 0.5 m), 2.0 equiv of **1**, 0.2 equiv of **3 d**, and 0.4 equiv of *p-*anisic acid in CH_2_Cl_2_ at 4 °C. Yields of isolated products are given. The d.r. values were determined by ^1^H NMR analysis of the crude reaction mixture. The *ee* values were determined by HPLC analysis using a chiral stationary phase.

The reaction was scaled up to obtain 1.06 grams of **4 a** (Scheme 2). By lowering the catalyst loading to 10 mol %, raising the temperature to 25 °C, and prolonging the reaction time to 72 hours the yield of the isolated product (93 %) and enantioselectivity (90 % *ee*) were comparable to those obtained on smaller scale.

**scheme 2:**
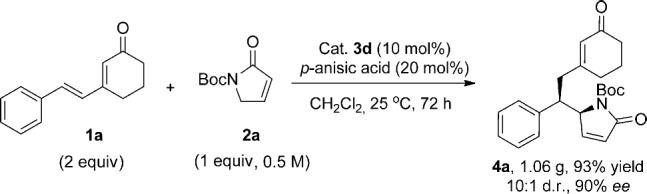
Large-scale preparation of 4 a.

N-Ts- and N-Cbz-protected α,β-unsaturated γ-butyrolactams were also compatible with the reaction conditions (Table 3, **4 q**–**4 v**). Compared to N-Boc-protected substrates, the enantioselectivity remained good (83–91 % *ee*). However the yields (45–72 %) and diastereoselectivities (7:1 to 10:1 d.r.) were slightly diminished.

**Table 3:** Scope of the DVMA with respect to the N-protected α,β-unsaturated γ-butyrolactams.^[a]^

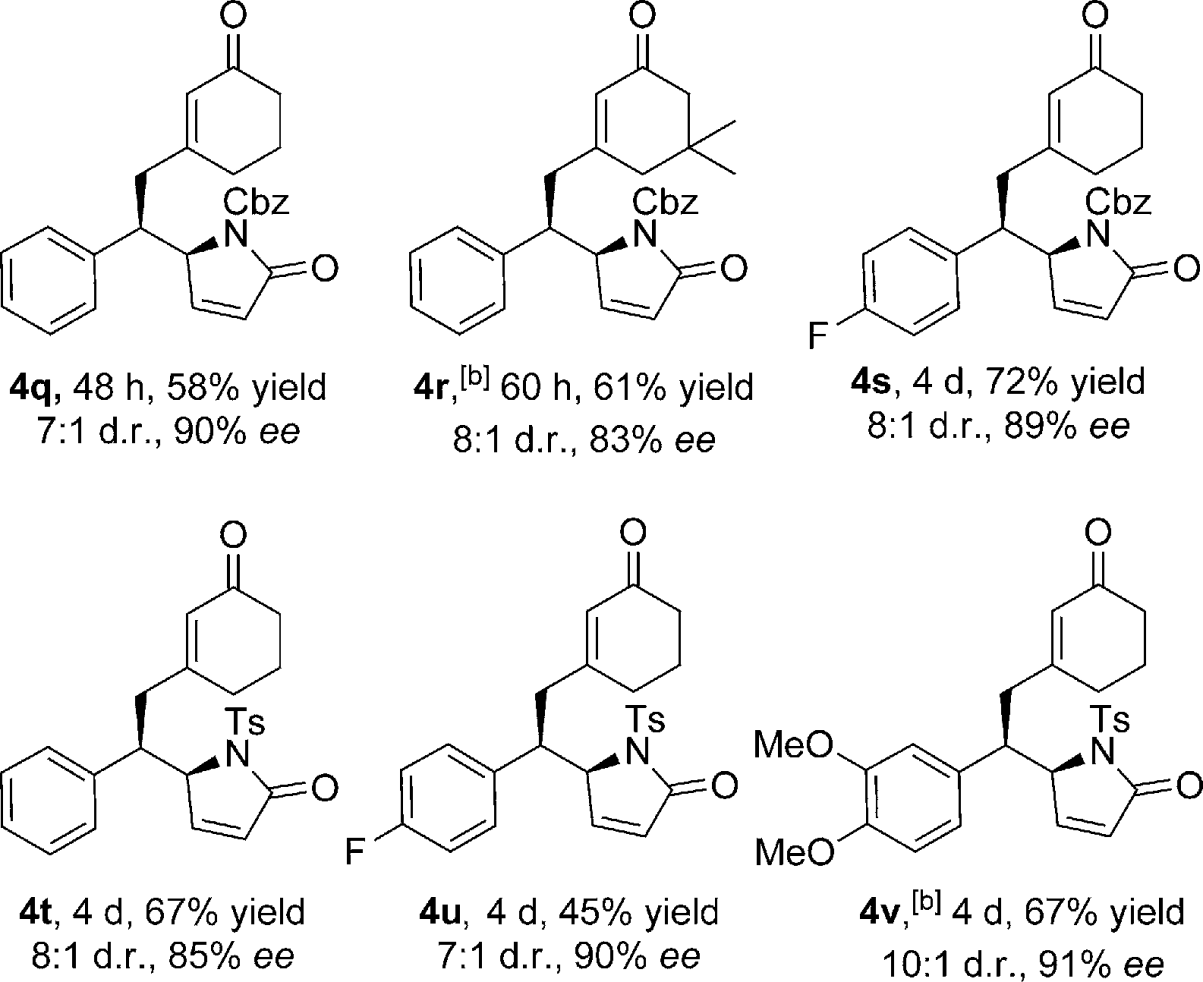

[a] Reactions performed using 1.0 equiv of **2** (0.2 mmol, 0.5 m), 2.0 equiv of **1**, 0.2 equiv of **3 d**, and 0.2 equiv *p*-anisic acid in CH_2_Cl_2_ at 4 °C, unless otherwise stated. Yields of isolated products are given. The d.r. values were determined by ^1^H NMR analysis of the crude reaction mixture. The *ee* values were determined by HPLC analysis using a chiral stationary phase. [b] Reaction performed at RT. Cbz=carboxybenzyl, Ts=4-toluenesulfonyl.

Interestingly, when switching from six- to five-membered cyclic dienones, a doubly vinylogous Michael addition/vinylogous Michael addition/isomerization cascade resulted (Table 4). The cascade reaction proceeded with excellent enantioselectivity (92–99 % *ee*), but poor to moderate diastereoselectivity (d.r. from 1:1 to 5:1). In our proposed mechanism for the cascade reaction (Scheme 3), the initial DVMA is followed by a vinylogous Michael addition from the γ-position of the cyclopentenone to the β-position of the butyrolactam. Migration of the C–C double bond to the other side of the carbonyl group may be ascribed to an isomerization via the dienamine of cyclopentenone,^12^ presumably driven by the thermodynamic stability of the product **6**.

**scheme 3:**
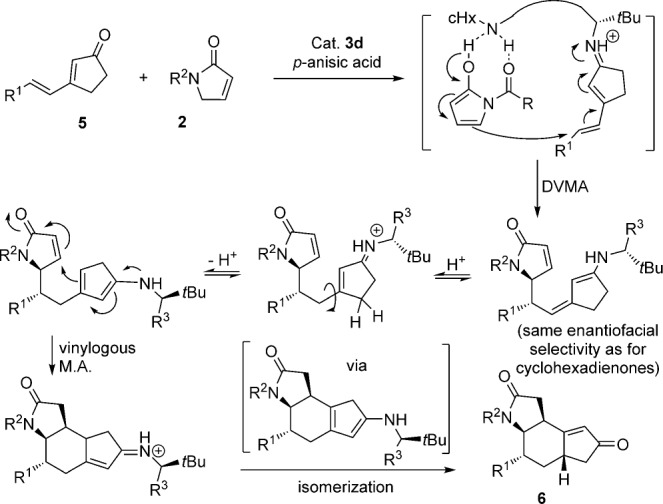
Postulated mechanism for the cascade reaction.

**Table 4:** Scope of the cascade reaction between 3-alkenyl cyclopent-2-enones and N-protected α,β-unsaturated γ-butyrolactams.^[a]^

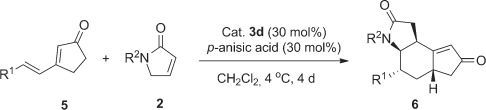
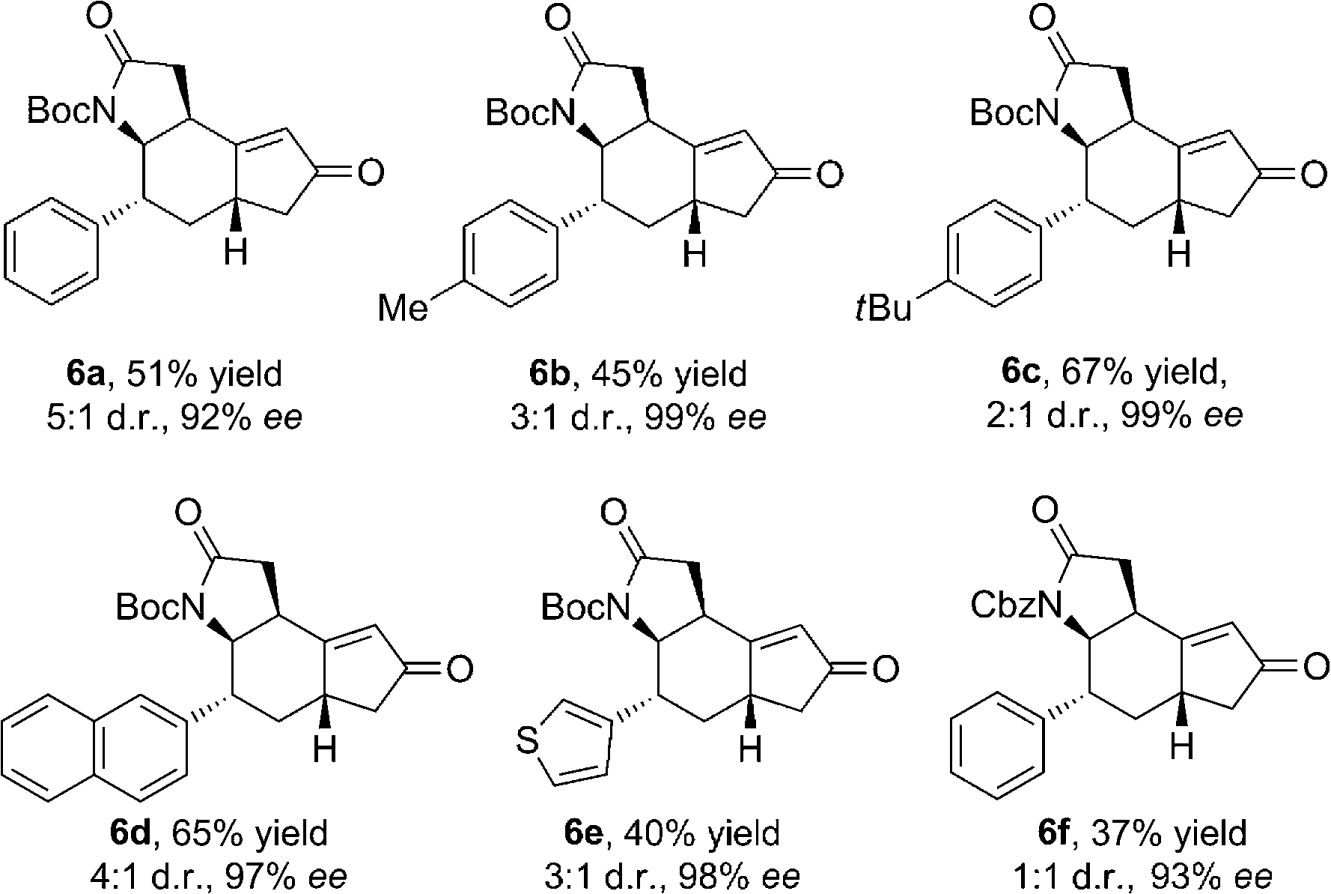

[a] Reactions performed using 1.0 equiv of **2** (0.2 mmol, 0.5 m), 2.0 equiv of **5**, 0.3 equiv of **3 d**, and 0.3 equiv *p-*anisic acid in CH_2_Cl_2_ at 4 °C for 4 days. Yields of the isolated products are given. The d.r. values were determined by ^1^H NMR analysis of the crude reaction mixture. The *ee* values were determined by HPLC analysis using a chiral stationary phase.

This mechanism is supported by the outcomes of the intermolecular vinylogous additions of 3-phenylcyclopent-2-enone (**7 a**) and 3-phenylcyclohex-2-enone (**7 b**) to the α,β-unsaturated γ-butyrolactam **2 a** (Scheme 4). A vinylogous Michael addition between **7 a** and **2 a**, which mimics the second step of the reaction cascade, took place with 30 mol % catalyst at 40 °C in 43 % yield. On the contrary, no reaction was observed using **7 b**.

**scheme 4:**
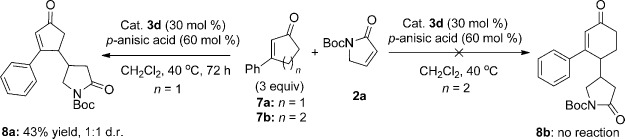
Vinylogous Michael addition.

The relative stereochemical configuration of the products of the cascade reaction (Table 4) was determined by single-crystal X-ray analysis of the compound **6 d**. The absolute configuration was assigned by analogy with that determined for the six-membered ring analogues, under the assumption that 3-alkenyl cyclopent-2-enones undergo the DVMA with the same enantiofacial selectivity.

In conclusion, we have developed a novel asymmetric direct doubly vinylogous Michael addition between α,β-unsaturated γ-butyrolactams and sterically congested β-substituted cyclic dienones, affording products with significant levels of diastereo- and enantioselectivity. Remote transmission of the stereochemical information was successfully realized through the two conjugated π systems by taking advantage of a bifunctional diamine catalyst. In addition, this method has provided access to chiral tricyclic γ-lactams with up to four newly formed stereocenters, generated from 3-alkenyl cyclopentenones substrates by an unprecedented vinylogous Michael addition/vinylogous Michael addition/isomerization cascade.

## Supporting Information

As a service to our authors and readers, this journal provides supporting information supplied by the authors. Such materials are peer reviewed and may be re-organized for online delivery, but are not copy-edited or typeset. Technical support issues arising from supporting information (other than missing files) should be addressed to the authors.

miscellaneous_information
