# Supplementary material for: Direct Catalytic Asymmetric Doubly Vinylogous Michael Addition of α,β-Unsaturated γ-Butyrolactams to Dienones
Source: Angew Chem Int Ed Engl. 2015 Jul 15;54(35):10249–53. doi: 10.1002/anie.201504276 (PMC4678421; doi:10.1002/anie.201504276)
Supplement: Supplementary file 1 [file anie0054-10249-sd1.pdf]

Angewandte Chemie
Eine Zeitschrift der Gesellschaft Deutscher Chemiker

Supporting Information

Direct Catalytic Asymmetric Doubly Vinylogous Michael Addition of α,β-Unsaturated γ-Butyrolactams to Dienones**

Xiaodong Gu, Tingting Guo, Yuanyuan Dai, Allegra Franchino, Jie Fei, Chuncheng Zou, Darren J. Dixon, and Jinxing Ye**

anie_201504276_sm_miscellaneous_information.pdf

Table of Contents

A: General Remarks and Starting Materials...................................... S2

B: Optimisation Tables.. S3

C: Doubly Vinylogous Michael Adducts and Derivatives................. S6

D: Determination of Absolute and Relative Configuration............ S18

E: NMR Spectra of Doubly Vinylogous Michael Adducts and Derivatives S23

F: HPLC Traces of Doubly Vinylogous Michael Adducts and Derivatives S53

A: General Remarks and Starting Materials

General Remarks

Proton nuclear magnetic resonance (^{1}H NMR) spectra and carbon nuclear magnetic resonance (^{13}C NMR) spectra were recorded on a Bruker AV-400 spectrometer (400 MHz and 100 MHz) or AV-500 spectrometer (500 MHz and 125 MHz). Chemical shifts (δ) for protons are reported in parts per million (ppm) downfield from tetramethylsilane and are referenced to the residual solvent peak ($CDCl_3$: δ 7.26). Chemical shifts (δ) for carbon are reported in parts per million downfield from tetramethylsilane and are referenced to the carbon resonances of the solvent ($CDCl_3$: δ 77.16). Data are reported as follows: chemical shift, multiplicity (br = broad, s = singlet, d = doublet, dd = doublet of doublets, t = triplet, dt = doublet of triplets, q = quartet, quint = quintet, m = multiplet), coupling constants (*J*) in Hertz (Hz), integration; "app" is used to denote the apparent splitting of a signal.

High resolution mass spectrometry (HRMS) was carried out using a Waters Micromass GCT spectrometer equipped with an ESI source.

Optical rotations were measured on an Autopol III automatic polarimeter (Rudolph Research analytical). $[\alpha]_D^T$ values are reported in 10^{-1} deg cm^2 g^{-1}; concentrations (*c*) are quoted in g/100 mL; D refers to the D-line of sodium (589 nm); temperatures (T) are given in degrees Celsius (℃).

Melting points were measured on a XT3A apparatus.

Enantiomeric excesses were determined by HPLC analysis on an Agilent HPLC 1200 Series instrument, using the chiral stationary phase column (25 cm x 4.6 mm internal diameter) specified in the individual experiment.

Starting Materials

All solvents and inorganic reagents were purchased from commercial suppliers and used without purification. Dienones **1a**, **1b**, **1e**, **1l**, **1m**, were prepared from 1,3-cyclohexanedione through a palladium-catalyzed Heck cross coupling reaction according to literature procedures.[1] **1c**, **1d**, **1f-1k**, **1n-1p** were prepared by a Horner-Wadsworth-Emmons reaction according to the literature.[2] 3-Alkenylcyclopent-2-enones (**5a-5e**) were prepared according to the same literature procedures.[1,2] The different *N*-protected α,β-unsaturated γ-butyrolactams **2** were synthesized following literature procedures.[3] Enones **7a** and **7b** were prepared from the Phenyl Grignard reagent and 3-ethoxycyclohex-2-enone, 3-ethoxycyclopent-2-enone according to literature procedures.[4]

---

(1) X. Fu, S. Zhang, J. Yin, T. L. McAllister, S. A. Jiang, C.-H. Tann, T. K. Thiruvengadam, F. Zhang, *Tetrahedron Letters* **2002**, *43*, 573-576.

(2) E. Wenkert, M. K. Schorp, *J. Org. Chem.* **1994**, *59*, 1943-1944.

(3) (a) Z. Tian, M. Rasmussen, S. J. Wittenberger, *Org. Process Res. Dev.* **2002**, *6*, 416-418. (b) C. Curti, B. Ranieri, L. Battistini, G. Rassu, V. Zambrano, G. Pelosi, G. Casiraghi, F. Zanardi, *Adv. Synth. Catal.* **2010**, *352*, 2011-2022.

(4) (a) X. Wang, C. M. Reisinger, B. List, *J. Am. Chem. Soc.* **2008**, 130, 6070-6071. (b) M. Augustin, L. Palais, A. Alexakis, *Angew. Chem. Int. Ed.* **2005**, 44, 1376-1378. (c) N. J. A. Martin, B. List, *J. Am. Chem. Soc.* **2006**, 128, 13368-13369.

B: Optimisation Tables

Table S1. Solvent screening using diamine **3d**/benzoic acid combination.[a]

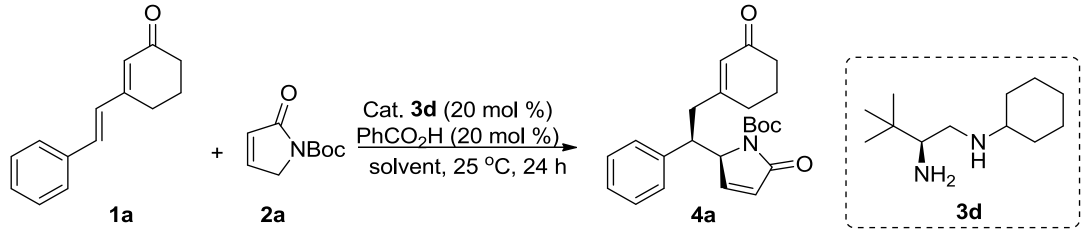

entry	solvent	conv. (%)[b]	dr[b]	ee (%)[c]
1	$CHCl_3$	55	7:1	89
2	DCM	74	19:1	90
3	1,2-DCE	88	12:1	91
4	toluene	96	9:1	79
5	THF	72	7:1	85
6	dioxane	25	6:1	88
7	MTBE	95	7:1	82
8	*i*-PrOH	65	2:1	79
9	AcOEt	86	6:1	86
10	DMF	34	5:1	77

[a] All reactions were performed using 1.0 equiv of **2a** (0.15 mmol, 0.5 M), 1.5 equiv of dienone **1a**, 0.2 equiv of catalyst **3d** and 0.2 equiv of $PhCO_2H$ at 25 ℃ for 24 hours. [b] Determined by ^1H NMR analysis of the crude reaction mixture. [c] Determined by HPLC on chiral stationary phase.

Table S2. Acid screening using diamine **3d** in DCM.[a]

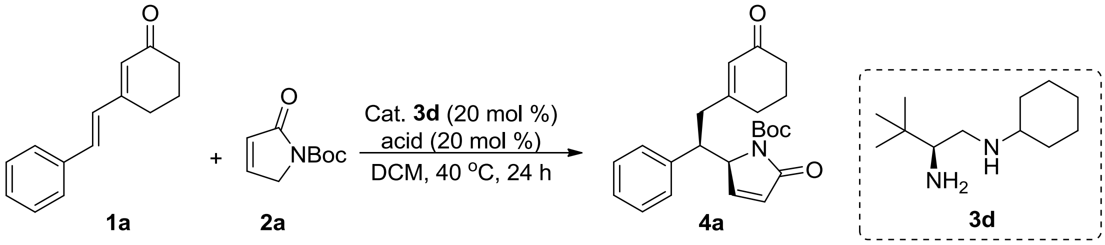

entry	acid	conv. (%)[b]	dr[b]	ee (%)[c]
1	$PhCO_2H$	95	11:1	88
2	*p*-MeO-$C_6H_4CO_2H$	91	19:1	89
3	*o*-F-$C_6H_4CO_2H$	85	13:1	87
4	*o*-NO_2-$C_6H_4CO_2H$	40	10:1	86
5	L-Boc-*tert*-Leu	92	15:1	86
6	D-Boc-Phe	88	13:1	86
7	L-Boc-Trp	74	6:1	85
8	CH_3CO_2H	87	9:1	87
9	CF_3CO_2H	26	10:1	82
10	CF_3SO_3H	Trace	ND	78

[a]All reactions were performed using 1.0 equiv of **2a** (0.15 mmol, 0.5 M), 1.5 equiv of dienone **1a**, 0.2 equiv of catalyst **3d** and 0.2 equiv of acid in DCM at 40 ℃ for 24 hours. [b] Determined by ^1H NMR analysis of the crude reaction mixture. [c] Determined by HPLC on chiral stationary phase.

Table S3. Temperature screening using diamine **3d**/*p*-anisic acid combination.[a]

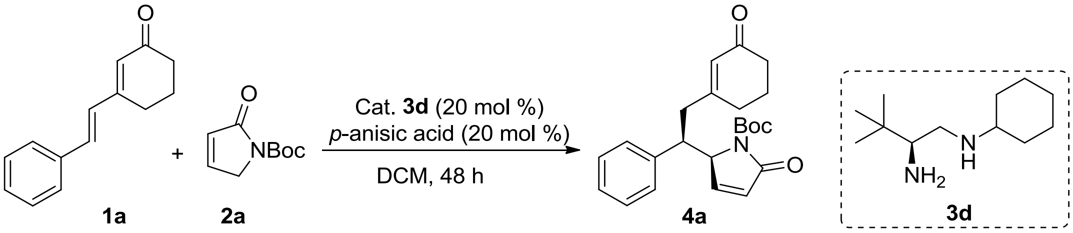

entry	T (°C)	conv. (%)[b]	dr[b]	ee (%)[c]
1	−40	36	14:1	90
2	4	76	19:1	91
3	25	85	13:1	90
4	40	91	19:1	89
5[d]	−40	37	13:1	90
6[d]	4	86	16:1	91

[a] All reactions were performed using 1.0 equiv of **2a** (0.15 mmol, 0.5 M), 1.5 equiv of dienone **1a**, 0.2 equiv of catalyst **3d** and 0.2 equiv of *p*-anisic acid in DCM for 48 hours, unless otherwise noted. [b] Determined by ^1H NMR analysis of the crude reaction mixture. [c] Determined by HPLC on chiral stationary phase. [d] Solvent: 1,2-DCE.

Table S4. Effect of the amount of acid on the doubly vinylogous Michael addition.[a]

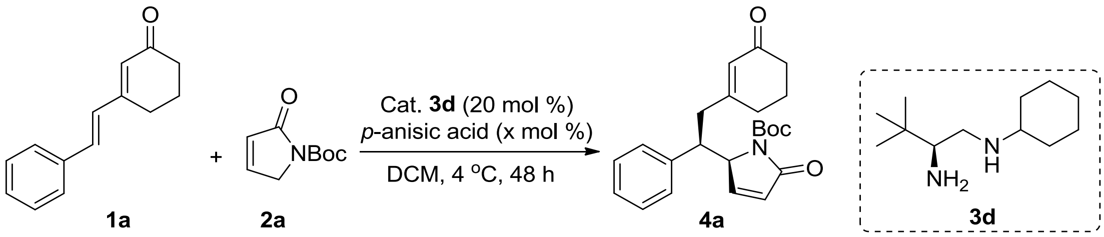

entry	acid loading (mol %)	conv.(%)[b]	dr[b]	ee (%)[c]
1	0	21	9:1	94
2	10	85	20:1	90
3	20	76	19:1	91
4	30	81	16:1	90
5	40	74	23:1	92

[a] All reactions were performed using 1.0 equiv of **2a** (0.15 mmol, 0.5 M), 1.5 equiv of dienone **1a**, 0.2 equiv of catalyst **3d** and *p*-anisic acid in DCM at 4 ℃ for 48 hours. [b] Determined by ^{1}H NMR analysis of the crude reaction mixture. [c] Determined by HPLC on chiral stationary phase.

Table S5. Effect of the ratio of starting materials on the doubly vinylogous Michael addition.[a]

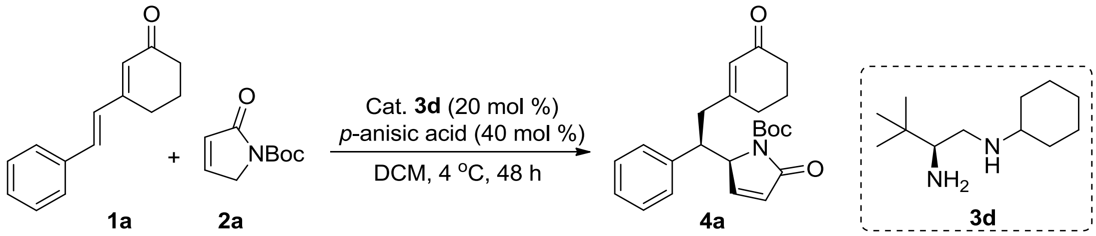

entry	**1a**:**2a**	conv. (%)[b]	dr[b]	ee (%)[c]
1	1:2	70	15:1	92
2	1.5:1	74	23:1	92
3	2:1	88	23:1	91
4	3:1	92	20:1	91

[a] All reactions were performed using 0.2 equiv of catalyst **3d** and 0.4 equiv of *p*-anisic acid in DCM at 4 ℃ for 48 hours. [b] Determined by ^{1}H NMR analysis of the crude reaction mixture. [c] Determined by HPLC on chiral stationary phase.

Table S6. Effect of the loading of catalyst on the doubly vinylogous Michael addition.[a]

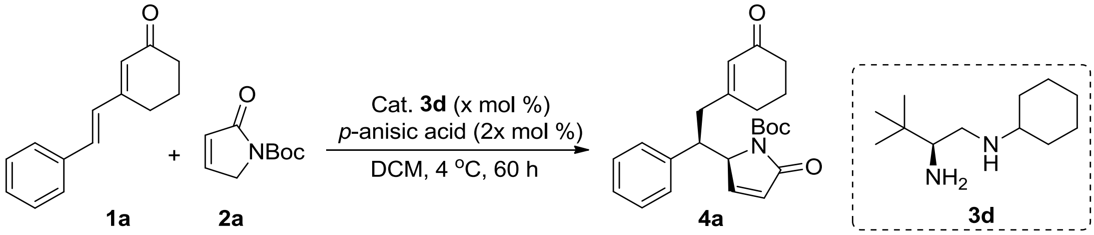

entry	x	yield (%)[b]	ee (%)[c]
1	10	82	91
2	20	95	92
3[d]	10	68	93

[a] All reactions were carried out using 1.0 equiv of **2a** (0.15 mmol, 0.5 M), 2.0 equiv of dienone **1a** in DCM at 4 ℃ for 60 hours, unless otherwise noted. [b] Isolated yield. [c] Determined by HPLC on chiral stationary phase. [d] Solvent: 1,2-DCE.

C: Doubly Vinylogous Michael Adducts and Derivatives

General Procedure: Catalyst **3d** (0.2-0.3 equiv as indicated in the single procedures), *p*-anisic acid (0.2-0.4 equiv as indicated in the single procedures) and *N*-substituted α,β-unsaturated γ-butyrolactam **2** (0.2 mmol, 1 equiv) were added in sequence to a solution of dienone **1** or **5** (0.4 mmol, 2 equiv) in DCM (0.4 mL). The reaction mixture was stirred at 4 °C for 48-96 h (as indicated in the single procedures), then the solvent was removed *in vacuo*. The residue was purified by flash silica gel chromatography to yield the desired product.

***tert*-Butyl (*S*)-2-oxo-5-((*R*)-2-(3-oxocyclohex-1-enyl)-1-phenylethyl)-2,5-dihydro-1*H*-pyrrole-1-carboxylate (4a)**

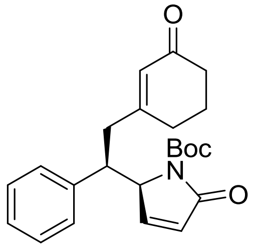

The reaction was carried out with 20 mol % of catalyst **3d** and 40 mol % of *p*-anisic acid for 60 hours. The product was obtained by column chromatography (PE/AcOEt = 3/1) as a white solid in 95% yield. M.p.: 133-134 °C; ^{1}H NMR (400 MHz, $CDCl_3$): δ 1.64 (s, 9H), 1.83-1.94 (m, 2H), 2.13-2.34 (m, 5H), 2.45 (dd, *J* = 11.6, 15.6 Hz, 1H), 4.05-4.10 (m, 1H), 4.77-4.79 (m, 1H), 5.72 (s, 1H), 6.19 (d, *J* = 6.0 Hz, 1H), 7.02 (dd, *J* = 1.6, 6.0 Hz, 1H), 7.25-7.32 (m, 3H), 7.36-7.40 (m, 2H); ^{13}C NMR (100 MHz, $CDCl_3$): δ 22.5, 28.3, 29.6, 33.8, 37.1, 42.6, 67.3, 83.7, 127.5, 127.8, 128.1, 128.2, 129.1, 138.2, 147.4, 149.5, 162.6, 169.1, 199.2; $[\alpha]_D^{25}$ −123 (*c* 1.00, CH_2Cl_2); HRMS (ESI) calcd for $(C_{23}H_{27}NO_4 + NH_4)^+$ 399.2284, found 399.2278; HPLC (DAICEL Chiralpak AS-H, *n*-hexane/ethanol = 7/3, flow 0.7 mL/min, detection at 240 nm) retention time = 13.7 min (major) and 26.4 min (minor), 91% ee.

tert-Butyl (_S_)-2-oxo-5-((_R_)-2-(3-oxocyclohex-1-enyl)-1-_p_-tolylethyl)-2,5-dihydro-1_H_-pyrrole-1-carboxylate (4b)

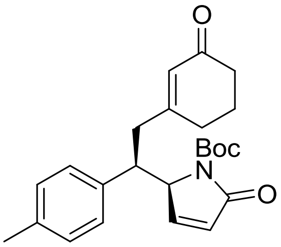

The reaction was carried out with 20 mol % of catalyst **3d** and 40 mol % of _p_-anisic acid for 48 hours. The product was obtained by column chromatography (PE/AcOEt = 4/1) as colorless oil in 81% yield. ^{1}H NMR (400 MHz, $CDCl_3$): δ 1.64 (s, 9H), 1.84-1.92 (m, 2H), 2.11-2.19 (m, 2H), 2.20-2.31 (m, 3H), 2.34 (s, 3H), 2.42 (dd, _J_ = 11.6, 15.6 Hz, 1H), 4.03 (td, _J_ = 4.0, 11.6 Hz, 1H), 4.75 ("app" quint, _J_ = 2.0 Hz, 1H), 5.70 (s, 1H), 6.18 (dd, _J_ = 1.6, 6.4 Hz, 1H), 7.03 (dd, _J_ = 2.0, 6.0 Hz, 1H), 7.12-7.19 (m, 4H); ^{13}C NMR (100 MHz, $CDCl_3$): δ 21.0, 22.5, 28.3, 29.6, 33.9, 37.1, 42.2, 67.4, 83.6, 127.5, 127.9, 128.1, 129.8, 135.1, 137.5, 147.6, 149.5, 162.8, 169.2, 199.3; $[\alpha]_D^{25}$ −120 (_c_ 1.00, CH_2Cl_2); HRMS (ESI) calcd for $(C_{24}H_{29}NO_4 + NH_4)^+$ 413.2440, found 413.2442; HPLC (DAICEL Chiralpak IA-H, _n_-hexane/ethanol = 7/3, flow 0.8 mL/min, detection at 240 nm) retention time = 18.7 min (major) and 20.3 min (minor), 90% ee.

tert-Butyl (_S_)-2-oxo-5-((_R_)-2-(3-oxocyclohex-1-enyl)-4-_tert_-butylphenyl)-2,5-dihydro- 1_H_-pyrrole-1-carboxylate (4c)

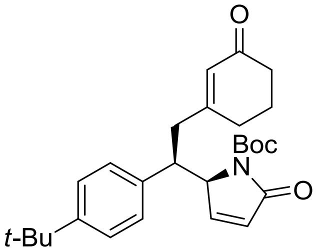

The reaction was carried out with 20 mol % of catalyst **3d** and 40 mol % of _p_-anisic acid for 3 days. The product was obtained by column chromatography (PE/AcOEt = 4/1) as colorless oil in 76% yield. ^{1}H NMR (400 MHz, $CDCl_3$): δ 1.32 (s, 9H), 1.65 (s, 9H), 1.84-1.95 (m, 2H), 2.14-2.31 (m, 5H), 2.45 (dd, _J_ = 11.6, 15.6 Hz, 1H), 4.03 (td, _J_ = 4.0, 10.8 Hz, 1H), 4.76-4.77 (m, 1H), 5.72 (s, 1H), 6.18 (d, _J_ = 6.0 Hz, 1H), 7.04 (m, 1H), 7.18 (d, _J_ = 8.0 Hz, 2H), 7.37 (d, _J_ = 8.0 Hz, 2H); ^{13}C NMR (100 MHz, $CDCl_3$): δ 22.4, 28.2, 29.6, 31.3, 33.8, 34.5, 37.1, 42.0, 67.4, 83.6, 126.0, 127.3, 127.6, 127.9, 135.0, 147.8, 149.4, 150.6 162.9, 169.4, 199.4; $[\alpha]_D^{25}$ −135 (_c_ 1.00, CH_2Cl_2); HRMS (ESI) calcd for $(C_{27}H_{35}NO_4 + NH_4)^+$ 455.2910, found 455.2911; HPLC (DAICEL Chiralpak IA-H, _n_-hexane/ethanol = 7/3, flow 0.8 mL/min, detection at 240 nm) retention time = 6.9 min (major) and 7.2 min (minor), 93% ee.

tert-Butyl (_S_)-2-oxo-5-((_R_)-2-(3-oxocyclohex-1-enyl)-1-_o_-tolylethyl)-2,5-dihydro-1_H_-pyrrole-1-carboxylate (4d)

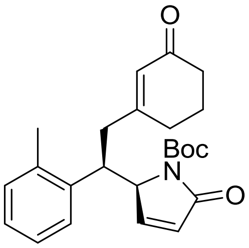

The reaction was carried out with 20 mol % of catalyst **3d** and 40 mol % of _p_-anisic acid for 63 hours. The product was obtained by column chromatography (PE/AcOEt = 4/1) as colorless oil in 73% yield. ^{1}H NMR (400 MHz, $CDCl_3$): δ 1.62 (s, 9H), 1.81-1.89 (m, 2H), 2.11-2.16 (m, 2H), 2.19-2.30 (m, 2H), 2.38 (dd, _J_ = 4.0, 15.6 Hz, 1H), 2.47, (dd, _J_ = 11.2, 15.2 Hz m, 1H), 2.55 (s, 3H), 4.31-4.37 (m, 1H), 4.71-4.73 (m, 1H), 5.67 (s, 1H), 6.19 (dd, _J_ = 1.2, 6.4 Hz, 1H), 7.01-7.07 (m, 2H), 7.16-7.26 (m, 3H); ^{13}C NMR (100 MHz, $CDCl_3$): δ 19.8, 22.5, 28.3, 29.7, 34.0, 37.1, 38.2, 64.6, 83.7, 126.2, 126.8, 127.4, 127.5, 128.1, 131.4, 135.9, 137.3, 147.9, 150.0, 162.9, 169.0, 199.3; $[\alpha]_D^{25}$ −115 (_c_ 1.00, CH_2Cl_2); HRMS (ESI) calcd for $(C_{24}H_{29}NO_4 + NH_4)^+$ 413.2440, found 413.2443; HPLC

(DAICEL Chiralpak AS-H, *n*-hexane/ethanol = 7/3, flow 0.7 mL/min, detection at 240 nm) retention time = 10.9 min (major) and 17.9 min (minor), 83% ee.

***tert*-Butyl (*S*)-2-oxo-5-((*R*)-2-(3-oxocyclohex-1-en-1-yl)-1-(2-(pivaloyloxy)phenyl) ethyl)-2,5-dihydro-1*H*-pyrrole-1-carboxylate (4e)**

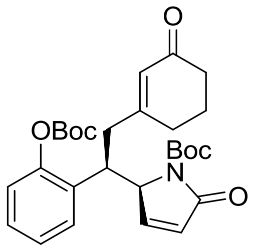

The reaction was carried out with 20 mol % of catalyst **3d** and 40 mol % of *p*-anisic acid for 50 hours. The product was obtained by column chromatography (PE/AcOEt = 4/1) as a white solid in 68% yield. M.p.: 78-79 °C; ^{1}H NMR (400 MHz, $CDCl_3$): δ 1.60 (s, 9H), 1.63 (s, 9H), 1.82-1.91 (m, 2H), 2.02-2.09 (m, 1H), 2.12-2.30 (m, 3H), 2.33-2.47 (m, 2H), 4.20-4.48 (m, 1H), 4.84-4.86 (m, 1H), 5.75 (s, 1H), 6.15 (dd, *J* = 1.6, 6.0 Hz, 1H), 6.99 (dd, *J* = 2.0, 6.0 Hz, 1H), 7.16-7.21 (m, 2H), 7.24-7.29 (m, 1H), 7.31-7.36 (m, 1H); ^{13}C NMR (100 MHz, $CDCl_3$): δ 22.4, 27.7, 28.2, 29.1, 33.9, 36.0, 37.1, 64.9, 83.4, 84.0, 123.4, 126.4, 127.9, 128.2, 128.2, 128.9, 130.2, 147.6, 149.9, 149.9, 152.2, 162.5, 168.7, 199.3; $[\alpha]_D^{25}$ −144 (c 1.00, CH_2Cl_2); HRMS (ESI) calcd for $(C_{28}H_{35}NO_7 + NH_4)^+$ 515.2757, found 515.2758; HPLC (DAICEL Chiralpak IA-H, *n*-hexane/ethanol = 7/3, flow 0.8 mL/min, detection at 254 nm) retention time = 11.3 min (major) and 12.8 min (minor), 88% ee.

***tert*-Butyl (*S*)-2-((*R*)-1-(3,4-dimethoxyphenyl)-2-(3-oxocyclohex-1-en-1-yl)ethyl)-5-oxo-2,5-dihydro-1*H*-pyrrole-1-carboxylate (4f)**

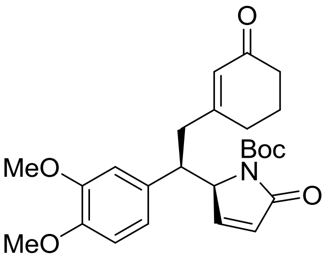

The reaction was carried out with 20 mol % of catalyst **3d** and 40 mol % of *p*-anisic acid for 72 hours. The product was obtained by column chromatography (PE/AcOEt = 4/1) as colorless oil in 80% yield. ^{1}H NMR (400 MHz, $CDCl_3$): δ 1.62 (s, 9H), 1.83-1.91 (m, 2H), 2.10-2.31 (m, 5H), 2.37 (dd, *J* = 11.2, 15.6 Hz, 1H), 3.86 (s, 3H), 3.87 (s, 3H), 3.95-4.01 (m, 1H), 4.76 (“app” quint, *J* = 2.0 Hz, 1H), 5.72 (s, 1H), 6.16 (dd, *J* = 1.6, 6.0 Hz, 1H), 6.74-6.77 (m, 2H), 6.82-6.85 (m, 1H), 7.01 (dd, *J* = 2.0, 6.0 Hz, 1H); ^{13}C NMR (100 MHz, $CDCl_3$): δ 22.5, 28.2, 29.6, 34.1, 37.1, 42.2, 55.9, 56.1, 67.3, 83.6, 111.5, 111.6, 119.9, 127.5, 128.1, 130.5, 147.7, 148.5, 149.3, 149.6, 162.8, 169.1, 199.3; $[\alpha]_D^{25}$ −135 (c 1.00, CH_2Cl_2); HRMS (ESI) calcd for $(C_{25}H_{31}NO_6 + NH_4)^+$ 459.2495, found 459.2499; HPLC (DAICEL Chiralpak AS-H, *n*-hexane/ethanol = 7/3, flow 0.7 mL/min, detection at 240 nm) retention time = 23.3 min (major) and 40.5 min (minor), 92% ee.

***tert*-Butyl (*S*)-2-((*R*)-1-(4-nitrophenyl)-2-(3-oxocyclohex-1-enyl)ethyl)-5-oxo- 2,5-dihydro-1*H*-pyrrole-1-carboxylate (4g)**

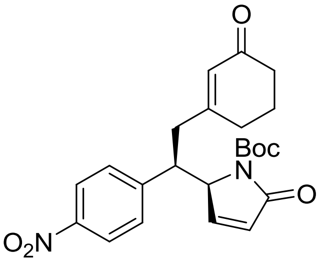

The reaction was carried out with 20 mol % of catalyst **3d** and 40 mol % of *p*-anisic acid for 48 hours. The product was obtained by column chromatography (PE/AcOEt = 1/1) as colorless oil in 71% yield. ^{1}H NMR (400 MHz, $CDCl_3$): δ 1.64 (s, 9H), 1.87-1.95 (m, 2H), 2.15-2.21 (m, 2H), 2.23-2.32 (m, 2H), 2.40 (dd, *J* = 4.0, 15.6 Hz, 1H), 2.48 (dd, *J* = 10.8, 15.6 Hz, 1H), 4.20- 4.25 (m, 1H), 4.76 (m, 1H), 5.68 (s, 1H), 6.24 (dd, *J* = 1.6, 6.0 Hz, 1H), 6.95 (dd, *J* = 1.6, 6.0 Hz, 1H), 7.48 (d, *J* = 8.8

Hz, 2H), 8.26 (d, J = 8.8 Hz, 2H); ^{13}C NMR (100 MHz, $CDCl_3$): δ 22.3, 28.1, 29.5, 33.8, 37.0, 42.3, 66.4, 84.1, 124.3, 127.6, 128.8, 129.0, 145.8, 146.1, 147.4, 149.6, 161.2, 168.4, 198.9; $[\alpha]_D^{25}$ −150 (c 1.00, CH_2Cl_2); HRMS (ESI) calcd for $(C_{23}H_{26}N_2O_6 + NH_4)^+$ 444.2135, found 444.2132; HPLC (DAICEL Chiralpak IA-H, *n*-hexane/ethanol = 7/3, flow 0.8 mL/min, detection at 240 nm) retention time = 11.7 min (major) and 13.3 min (minor), 91% ee.

***tert*-Butyl (*S*)-2-((*R*)-1-(3-bromophenyl)-2-(3-oxocyclohex-1-enyl)ethyl)-5-oxo-2,5-dihydro-1*H*-pyrrole-1-carboxylate (4h)**

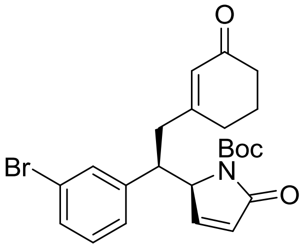

The reaction was carried out with 20 mol % of catalyst **3d** and 40 mol % of *p*-anisic acid for 60 hours. The product was obtained by column chromatography (PE/AcOEt = 3/1) as colorless oil in 78% yield. ^{1}H NMR (400 MHz, $CDCl_3$): δ 1.62 (s, 9H), 1.85-1.93 (m, 2H), 2.07-2.19 (m, 2H), 2.23-2.32 (m, 3H), 2.39 (dd, J = 10.8, 15.6 Hz, 1H), 4.01-4.06 (m, 1H), 4.75 (“app” quint, J = 2.0 Hz, 1H), 5.68 (s, 1H), 6.19 (dd, J = 1.6, 6.4 Hz, 1H), 6.98 (dd, J = 2.0, 6.4 Hz, 1H), 7.17-7.20 (m, 1H), 7.22-7.26 (m, 1H), 7.40-7.45 (m, 2H); ^{13}C NMR (100 MHz, $CDCl_3$): δ 22.4, 28.2, 29.6, 33.6, 37.0, 42.1, 66.9, 83.9, 123.2, 126.5, 127.4, 128.4, 130.6, 131.0, 131.2, 140.7, 146.8, 149.5, 161.9, 168.8, 199.1; $[\alpha]_D^{25}$ −263 (c 1.00, CH_2Cl_2); HRMS (ESI) calcd for $(C_{23}H_{26}BrNO_4 + NH_4)^+$ 479.1308, found 479.1368; HPLC (DAICEL Chiralpak AS-H, *n*-hexane/ethanol = 7/3, flow 0.7 mL/min, detection at 240 nm) retention time = 21.0 min (major) and 51.8 min (minor), 92% ee.

***tert*-Butyl (*S*)-2-((*R*)-1-(4-fluorophenyl)-2-(3-oxocyclohex-1-enyl)ethyl)-5-oxo- 2,5-dihydro-1*H*-pyrrole-1-carboxylate (4i)**

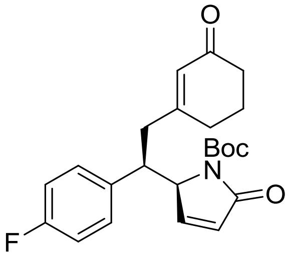

The reaction was carried out with 20 mol % of catalyst **3d** and 40 mol % of *p*-anisic acid for 56 hours. The product was obtained by column chromatography (PE/AcOEt = 4/1) as yellow solid in 76% yield. M.p.: 168-169 °C; ^{1}H NMR (400 MHz, $CDCl_3$): δ 1.61 (s, 9H), 1.83-1.91 (m, 2H), 2.10-2.42 (m, 6H), 4.01-4.07 (m, 1H), 4.71-4.74 (m, 1H), 5.67 (s, 1H), 6.17 (d, J = 6.4 Hz, 1H), 6.97-7.00 (m, 1H), 7.02-7.07 (m, 2H), 7.20-7.24 (m, 2H); ^{13}C NMR (100 MHz, $CDCl_3$): δ 22.4, 28.2, 29.6, 34.1, 37.1, 41.9, 67.1, 83.7, 116.1 (d, $^2J_{CF}$ = 21.3 Hz), 127.5, 128.3, 129.6 (d, $^3J_{CF}$ = 8.0 Hz), 134.0 (d, $^4J_{CF}$ = 3.2 Hz), 147.1, 149.5, 162.0 (d, $^1J_{CF}$ = 245.1 Hz), 162.3, 168.9, 199.1; $[\alpha]_D^{25}$ −215 (c 1.00, CH_2Cl_2); HRMS (ESI) calcd for $(C_{23}H_{26}FNO_4 + NH_4)^+$ 417.2190, found 417.2189; HPLC (DAICEL Chiralpak AS-H, *n*-hexane/ethanol = 7/3, flow 0.7 mL/min, detection at 240 nm) retention time = 15.6 min (major) and 26.5 min (minor), 93% ee.

tert-Butyl (S)-2-((R)-1-(4-chlorophenyl)-2-(3-oxocyclohex-1-enyl)ethyl)-5-oxo- 2,5-dihydro-1H-pyrrole-1-carboxylate (4j)

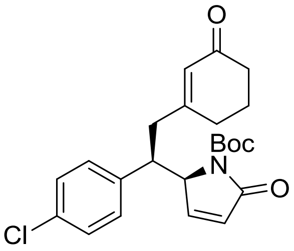

The reaction was carried out with 20 mol % of catalyst **3d** and 40 mol % of *p*-anisic acid for 72 hours. The product was obtained by column chromatography (PE/AcOEt = 4/1) as yellow oil in 77% yield. ^{1}H NMR (400 MHz, $CDCl_3$): δ 1.63 (s, 9H), 1.85-1.93 (m, 2H), 2.12-2.35 (m, 5H), 2.40 (dd, *J* = 11.2, 15.6 Hz, 1H), 4.03-4.09 (m, 1H), 4.73-4.75 (m, 1H), 5.69 (s, 1H), 6.20 (dd, *J* = 1.6, 6.4 Hz, 1H), 6.98 (dd, *J* = 1.6, 6.4 Hz, 1H), 7.21 (d, *J* = 8.4 Hz, 2H), 7.36 (d, *J* = 8.4 Hz, 2H); ^{13}C NMR (100 MHz, $CDCl_3$): δ 22.4, 28.2, 29.6, 33.8, 37.1, 42.0, 67.0, 83.9, 127.6, 128.4, 129.4, 133.7, 136.7, 146.9, 149.5, 162.1, 168.9, 199.1; $[\alpha]_D^{25}$ −160 (c 1.00, CH_2Cl_2); HRMS (ESI) calcd for $(C_{23}H_{26}ClNO_4 + NH_4)^+$ 433.1894, found 433.1895; HPLC (DAICEL Chiralpak IA-H, *n*-hexane/ethanol = 7/3, flow 0.8 mL/min, detection at 240 nm) retention time = 8.0 min (major) and 8.8 min (minor), 90% ee.

tert-Butyl (R)-2-oxo-5-((S)-1-(3-oxocyclohex-1-enyl)propan-2-yl)-2,5-dihydro- 1H-pyrrole-1-carboxylate (4k)

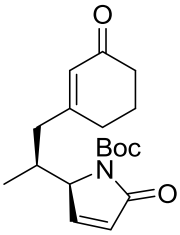

The reaction was carried out with 20 mol % of catalyst **3d** and 40 mol % of *p*-anisic acid for 65 hours. The product was obtained by column chromatography (PE/AcOEt = 3/1) as colorless oil in 69% yield. ^{1}H NMR (400 MHz, $CDCl_3$): δ 1.74 (d, *J* = 6.8 Hz, 3H), 1.24-1.32 (m, 1H), 1.55 (s, 9H), 1.63 (dd, *J* = 10.8, 13.6 Hz, 1H), 1.92-1.99 (m, 2H), 2.02-2.08 (m, 1H), 2.11-2.21 (m, 1H), 2.32-2.36 (m, 2H), 2.71-2.82 (m, 1H), 4.64-4.66 (m, 1H), 5.79 (s, 1H), 6.17 (d, *J* = 6.4 Hz, 1H), 7.19 (dd, *J* = 1.6, 6.0 Hz, 1H); ^{13}C NMR (100 MHz, $CDCl_3$): δ 16.9, 22.6, 28.1, 29.3, 31.7, 37.19, 38.5, 66.7, 83.3, 127.4, 128.2, 147.3, 149.6, 163.5, 169.0, 199.4; $[\alpha]_D^{25}$ −256 (*c* 1.00, CH_2Cl_2); HRMS (ESI) calcd for $(C_{18}H_{25}NO_4 + NH_4)^+$ 337.2127, found 337.2131; HPLC (DAICEL Chiralpak AS-H, *n*-hexane/ethanol = 7/3, flow 0.7 mL/min, detection at 240 nm) retention time = 19.3 min (major) and 26.0 min (minor), 92% ee.

tert-Butyl (R)-2-oxo-5-((S)-1-(3-oxocyclohex-1-enyl)pentan-2-yl)-2,5-dihydro- 1H-pyrrole-1-carboxylate (4l)

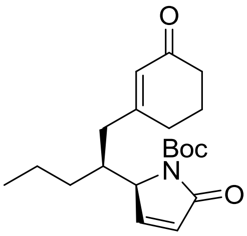

The reaction was carried out with 20 mol % of catalyst **3d** and 40 mol % of *p*-anisic acid for 4 days. The product was obtained by column chromatography (PE/AcOEt = 3/1) as colorless oil in 54% yield. Characterized as a 11:1 mixture of diastereomers. Data are given for major diastereomer only. ^{1}H NMR (400 MHz, $CDCl_3$): δ 0.97 (t, *J* = 6.8 Hz, 3H), 1.32-1.48 (m, 4H), 1.57 (s, 9H), 1.71 (dd, *J* = 9.6, 14.4 Hz, 1H), 1.95-1.99 (m, 3H), 2.07-2.27 (m, 2H), 2.33-2.38 (m, 2H), 2.62-2.68 (m, 1H), 4.79 (m, 1H), 5.79 (s, 1H), 6.19 (dd, *J* = 1.6, 6.0 Hz, 1H), 7.17 (dd, *J* = 1.6, 6.0 Hz, 1H); ^{13}C NMR (100 MHz, $CDCl_3$): δ 14.2, 20.6, 22.6, 28.2, 29.4, 34.1, 36.3, 37.2, 37.8, 64.5, 83.4, 127.3, 128.4, 147.3, 149.4, 163.8, 169.2, 199.4; $[\alpha]_D^{25}$ –238 (c 1.00, CH_2Cl_2); HRMS (ESI) calcd for $(C_{20}H_{29}NO_4 + NH_4)^+$ 365.2440, found 365.2440; HPLC (DAICEL Chiralpak IA-H, *n*-hexane/ethanol = 30/1, flow 0.8 mL/min,

detection at 240 nm) retention time = 37.5 min (minor) and 40.3 min (major), 89% ee.

***tert*-Butyl (*S*)-2-((*R*)-2-(5,5-dimethyl-3-oxocyclohex-1-enyl)-1-phenylethyl)-5-oxo-2,5-dihydro-1*H*-pyrrole-1-carboxylate (4m)**

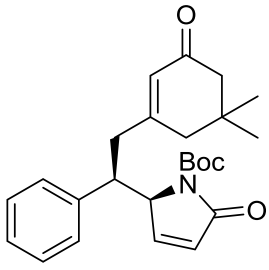

The reaction was carried out with 20 mol % of catalyst **3d** and 40 mol % of *p*-anisic acid for 48 hours. The product was obtained by column chromatography (PE/AcOEt = 4/1) as colorless oil in 57% yield. ^{1}H NMR (400 MHz, $CDCl_3$): δ 0.84 (s, 3H), 0.93 (s, 3H), 1.66 (s, 9H), 1.98-2.14 (m, 4H), 2.22-2.26 (m, 1H), 2.43 (dd, *J* = 12.0, 15.2 Hz, 1H), 4.07 (td, *J* = 3.6, 12.0 Hz, 1H), 4.78 ("app" quint, *J* = 2.0 Hz, 1H), 5.70 (s, 1H), 6.20 (dd, *J* = 1.6, 6.0 Hz, 1H) , 7.03 (dd, *J* = 2.0, 6.0 Hz, 1H), 7.24-7.32 (m, 3H), 7.35-7.39 (m, 2H); ^{13}C NMR (100 MHz, $CDCl_3$): δ 27.9, 28.3, 33.6, 33.6, 42.6, 43.8, 50.8, 67.2, 83.7, 126.7, 127.9, 128.1, 128.2, 129.1, 137.9, 147.5, 149.5, 160.2, 169.2, 199.2; $[\alpha]_D^{25}$ −122 (c 1.00, CH_2Cl_2); HRMS (ESI) calcd for $(C_{25}H_{31}NO_4 + NH_4)^+$ 427.2597, found 427.2601; HPLC (DAICEL Chiralpak IA-H, *n*-hexane/ethanol = 4/1, flow 0.6 mL/min, detection at 240 nm) retention time = 9.6 min (major) and 10.1 min (minor), 91% ee.

***tert*-Butyl (*S*)-2-((*R*)-2-(6,6-dimethyl-3-oxocyclohex-1-enyl)-1-phenylethyl)-5-oxo-2,5-dihydro-1*H*-pyrrole-1-carboxylate (4n)**

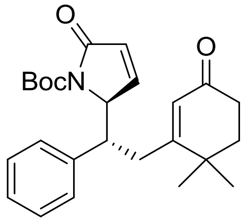

The reaction was carried out with 20 mol % of catalyst **3d** and 40 mol % of *p*-anisic acid for 48 hours. The product was obtained by column chromatography (PE/AcOEt = 4/1) as colorless oil in 66% yield. ^{1}H NMR (400 MHz, $CDCl_3$): δ 1.14 (s, 3H), 1.15 (s, 3H), 1.64 (s, 9H), 1.80 (t, *J* = 6.8 Hz, 2H), 2.25-2.36 (m, 3H), 2.45 (dd, *J* = 12.0, 18.0 Hz, 1H), 4.03-4.14 (m, 1H), 4.78-4.80 (m, 1H), 5.53 (s, 1H), 6.22 (dd, *J* = 0.8, 6.0 Hz, 1H) , 7.04 (dd, *J* = 2.0, 6.0 Hz, 1H), 7.25-7.28 (m, 2H), 7.30-7.32 (m, 1H), 7.36-7.41 (m, 2H); ^{13}C NMR (100 MHz, $CDCl_3$): δ 26.4, 26.7, 27.4, 28.2, 34.1, 35.8, 38.0, 42.5, 67.5, 83.8, 126.0, 127.8, 128.0, 128.0, 129.2, 138.4, 147.7, 149.4, 168.5, 169.3, 198.9; $[\alpha]_D^{25}$ −129 (c 1.00, CH_2Cl_2); HRMS (ESI) calcd for $(C_{25}H_{31}NO_4 + NH_4)^+$ 427.2597, found 427.2602; HPLC (DAICEL Chiralpak IA-H, *n*-hexane/ethanol = 9/1, flow 0.8 mL/min, detection at 240 nm) retention time = 12.2 min (minor) and 16.1 min (major), 94% ee.

***tert*-Butyl (*S*)-2-((*R*)-1-(naphthalen-2-yl)-2-(3-oxocyclohex-1-enyl)ethyl)-5-oxo-2,5-dihydro-1*H*-pyrrole-1-carboxylate (4o)**

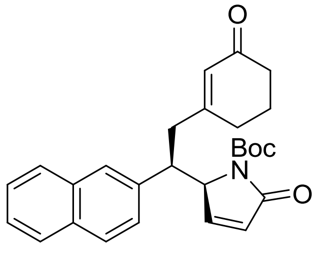

The reaction was carried out with 20 mol % of catalyst **3d** and 40 mol % of *p*-anisic acid for 4 days. The product was obtained by column chromatography (PE/AcOEt = 3/1) as colorless oil in 65% yield. ^{1}H NMR (400 MHz, $CDCl_3$): δ 1.67 (s, 9H), 1.82-1.94 (m, 2H), 2.12-2.29 (m, 4H), 2.41 (dd, *J* = 3.2, 15.6 Hz, 1H), 2.59 (dd, *J* = 11.6, 15.6 Hz, 1H), 4.25 (td, *J* = 4.0, 11.2 Hz, 1H), 4.88 ("app" quint, *J* = 2 Hz, 1H), 5.77 (s, 1H), 6.21 (dd, *J* = 1.6, 6.0 Hz, 1H), 7.05 (dd, *J* = 1.6, 6.0 Hz, 1H), 7.40 (dd, *J* = 1.6, 8.4 Hz, 1H), 7.48-7.54 (m, 2H), 7.70 (s, 1H), 7.80-7.89 (m, 3H); ^{13}C NMR (100 MHz, $CDCl_3$): δ 22.5, 28.3, 29.7, 33.9, 37.1, 42.7, 67.3, 83.8, 125.9, 126.4, 126.7, 127.0,

127.5, 127.7, 127.8, 128.2, 129.1, 132.8, 133.4, 135.7, 147.4, 149.6, 162.6, 169.1, 199.2; $[\alpha]_D^{25}$ −128 (*c* 1.00, CH_2Cl_2); HRMS (ESI) calcd for $(C_{27}H_{29}NO_4 + NH_4)^+$ 449.2440, found 449.2439; HPLC (DAICEL Chiralpak IA-H, *n*-hexane/ethanol = 7/3, flow 0.8 mL/min, detection at 240 nm) retention time = 8.7 min (major) and 10.6 min (minor), 92% ee.

***tert*-Butyl (*S*)-2-oxo-5-((*R*)-2-(3-oxocyclohex-1-enyl)-1-(thiophen-3-yl)ethyl)-2,5-dihydro-1*H*-pyrrole-1-carboxylate (4p)**

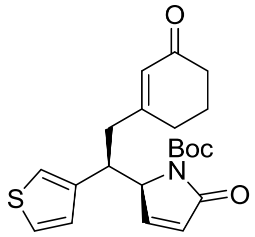

The reaction was carried out with 20 mol % of catalyst **3d** and 40 mol % of *p*-anisic acid for 4 days. The product was obtained by column chromatography (PE/AcOEt = 3/1) as a white solid in 51% yield. M.p.: 170-171 °C; ^{1}H NMR (400 MHz, $CDCl_3$): δ 1.63 (s, 9H), 1.87-1.95 (m, 2H), 2.08-2.23 (m, 2H), 2.24-2.40 (m, 4H), 4.14-4.19 (m, 1H), 4.82-4.84 (m, 1H), 5.75 (s, 1H), 6.18-6.21 (m, 1H), 7.01-7.05 (m, 2H), 7.11 (s, 1H), 7.36-7.39 (m, 1H); ^{13}C NMR (100 MHz, $CDCl_3$): δ 22.5, 28.2, 29.5, 35.4, 37.1, 38.3, 66.7, 83.7, 122.3, 126.9,126.9, 127.4, 128.2, 139.6, 147.7, 149.5, 162.6, 169.1, 199.3; $[\alpha]_D^{25}$ −220 (*c* 1.00, CH_2Cl_2); HRMS (ESI) calcd for $(C_{21}H_{25}NO_4S + Na)^+$ 410.1402, found 410.1406; HPLC (DAICEL Chiralpak IA-H, *n*-hexane/ethanol = 7/3, flow 0.8 mL/min, detection at 240 nm) retention time = 8.7 min (major) and 10.1 min (minor), 91% ee.

Benzyl (*S*)-2-oxo-5-((*R*)-2-(3-oxocyclohex-1-enyl)-1-phenylethyl)-2,5-dihydro- 1*H*-pyrrole-1-carboxylate (4q)

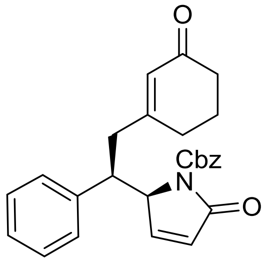

The reaction was carried out with 20 mol % of catalyst **3d** and 20 mol % of *p*-anisic acid for 48 hours. The product was obtained by column chromatography (PE/AcOEt = 1/1) as a white solid in 58% yield. M.p.: 114-115 °C; ^{1}H NMR (400 MHz, $CDCl_3$): δ 1.75-1.82 (m, 2H), 1.92-2.01 (m, 2H), 2.09-2.26 (m, 3H), 2.38-2.45 (m, 1H), 4.01 (td, *J* = 4.0, 11.2 Hz, 1H), 4.81-4.83 (m, 1H), 5.33 (d, *J* = 4.0 Hz, 1H), 5.47 (d, *J* = 4.0 Hz, 1H), 5.66 (s, 1H), 6.19-6.22 (m, 1H), 7.06 (dd, *J* = 2.0, 6.0 Hz, 1H), 7.16-7.19 (m, 2H), 7.27-7.43 (m, 6H), 7.51-7.54 (m, 2H); ^{13}C NMR (100 MHz, $CDCl_3$): δ 22.4, 29.3, 34.0, 37.1, 42.4, 67.3, 68.4, 127.6, 127.8, 127.9, 128.1, 128.6, 128.7, 128.8, 129.1, 135.1, 138.0, 148.1, 150.9, 162.3, 168.7, 199.2; $[\alpha]_D^{25}$ −129 (*c* 1.00, CH_2Cl_2); HRMS (ESI) calcd for $(C_{26}H_{25}NO_4 + NH_4)^+$ 433.2127, found 433.2130; HPLC (DAICEL Chiralpak IA-H, *n*-hexane/ethanol = 7/3, flow 0.8 mL/min, detection at 240 nm) retention time = 15.5 min (major) and 22.5 min (minor), 90% ee.

Benzyl (*S*)-2-((*R*)-2-(5,5-dimethyl-3-oxocyclohex-1-enyl)-1-phenylethyl)-5-oxo-2,5-dihydro-1*H*-pyrrole-1-carboxylate (4r)

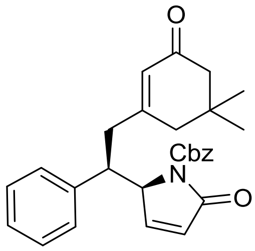

The reaction was carried out with 20 mol % of catalyst **3d** and 20 mol % of *p*-anisic acid for 60 hours. The product was obtained by column chromatography (PE/AcOEt = 2/1) as colorless oil in 61% yield. ^{1}H NMR (400 MHz, $CDCl_3$): δ 0.79 (s, 3H), 0.90 (s, 3H), 1.88 (d, *J* = 18.0 Hz, 1H), 1.94 (d, *J* = 18.0 Hz, 1H), 2.03 (d, *J* = 16.0 Hz, 1H), 2.10 (d, *J* = 16.0 Hz, 1H), 2.23 (dd, *J* = 2.8, 15.2 Hz, 1H), 2.41 (dd, *J* = 12.0, 15.2 Hz, 1H), 4.03 (td, *J* = 4.0, 12.0 Hz, 1H), 4.84 ("app" quint, *J* = 2.0 Hz, 1H), 5.36 (d, *J* = 12.0 Hz, 1H), 5.49 (d, *J* = 12.4 Hz, 1H), 5.68 (s, 1H), 6.23 (dd, *J* = 1.6, 6.4 Hz, 1H), 7.10 (dd, *J* = 2.0, 6.0 Hz, 1H), 7.16-7.18 (m, 2H), 7.30-7.46 (m, 6H), 7.52-7.55 (m, 2H); ^{13}C NMR (100 MHz, $CDCl_3$): δ 27.9, 28.2, 33.5, 33.8, 42.5, 43.5, 50.8, 67.2, 68.5, 126.8, 127.8, 128.2, 128.4, 128.8, 128.8, 129.1, 135.1, 137.8, 148.2, 151.0, 160.0, 168.7, 199.4; $[\alpha]_D^{25}$ −135 (*c* 1.00, CH_2Cl_2); HRMS(ESI) calcd for $(C_{28}H_{29}NO_4 + NH_4)^+$ 461.2440, found 461.2442; HPLC (DAICEL Chiralpak IA-H, *n*-hexane/ethanol = 7/3, flow 0.8 mL/min, detection at 240 nm) retention time = 11.2 min (major) and 12.6 min (minor), 83% ee.

Benzyl (*S*)-2-((*R*)-1-(4-fluorophenyl)-2-(3-oxocyclohex-1-enyl)ethyl)-5-oxo-2,5-dihydro-1*H*-pyrrole-1-carboxylate (4s)

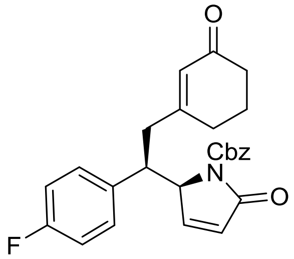

The reaction was carried out with 20 mol % of catalyst **3d** and 20 mol % of *p*-anisic acid for 4 days. The product was obtained by column chromatography (PE/AcOEt = 3/1) as Light yellow oil in 72% yield. ^{1}H NMR (400 MHz, $CDCl_3$): δ 1.76-1.84 (m, 2H), 1.88-2.03 (m, 2H), 2.13-2.28 (m, 3H), 2.37 (dd, *J* = 11.2, 15.6 Hz, 1H), 4.00 (td, *J* = 4.0, 11.2 Hz, 1H), 4.79 ("app" quint, *J* = 2.0 Hz, 1H), 5.34 (d, *J* = 12.4 Hz, 1H), 5.47 (d, *J* = 12.0 Hz, 1H), 5.64 (s, 1H), 6.22 (dd, *J* = 1.6, 6.0 Hz, 1H), 7.02-7.07 (m, 3H), 7.13-7.17 (m, 2H), 7.35-7.45 (m, 3H), 7.51-7.55 (m, 2H); ^{13}C NMR (100 MHz, $CDCl_3$): δ 22.4, 29.3, 34.2, 37.1, 41.6, 67.2, 68.4, 116.1 (d, $^{2}J_{CF}$ = 21.3 Hz), 127.6, 128.1, 128.6, 128.8, 128.8, 129.6 (d, $^{3}J_{CF}$ = 7.9 Hz), 133.7 (d, $^{4}J_{CF}$ = 3.3 Hz), 135.1, 147.7, 150.9, 161.9, 162.1 (d, $^{1}J_{CF}$ = 245.8 Hz), 168.5, 199.1; $[\alpha]_D^{25}$ −186 (*c* 1.00, CH_2Cl_2); HRMS (ESI) calcd for $(C_{26}H_{24}FNO_4 + NH_4)^+$ 451.2033, found 449.2030; HPLC (DAICEL Chiralpak IA-H, *n*-hexane/ethanol = 7/3, flow 0.8 mL/min, detection at 240 nm) retention time = 14.3 min (major) and 17.9 min (minor), 91% ee.

(*S*)-5-((*R*)-2-(3-oxocyclohex-1-enyl)-1-phenylethyl)-1-tosyl-1,5-dihydro-2*H*-pyrrol-2-one (4t)

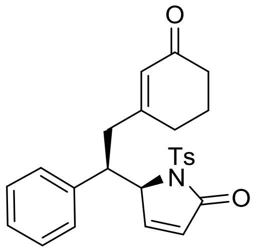

The reaction was carried out with 20 mol % of catalyst **3d** and 20 mol % of *p*-anisic acid for 4 days. The product was obtained by column chromatography (PE/AcOEt = 3/1) as a yellow solid in 67% yield. M.p.: 82-83 ºC; ^{1}H NMR (400 MHz, $CDCl_3$): δ 1.79-1.93 (m, 2H), 2.07-2.33 (m, 5H), 2.39 (dd, *J* = 12.0, 16.0 Hz, 1H), 2.45 (s, 3H), 4.25 (td, *J* = 3.6, 12.0 Hz, 1H), 4.94-4.96 (m, 1H), 5.67 (s, 1H), 6.11 (dd, *J* = 1.2, 6.0 Hz, 1H), 7.07 (dd, *J* = 2.0, 6.0

Hz, 1H), 7.30-7.36 (m, 5H), 7.40 (d, *J* = 7.6 Hz, 2H), 8.01 (d, *J* = 8.0 Hz, 2H); ^{13}C NMR (100 MHz, $CDCl_3$): δ 21.7, 22.5, 29.4, 33.4, 37.2, 43.6, 69.3, 127.2, 127.6, 128.0, 128.1, 128.2, 129.3, 129.8, 135.6, 137.6, 145.4, 148.9, 162.3, 169.0, 199.3; $[\alpha]_D^{25}$ −137 (*c* 1.00, CH_2Cl_2); HRMS (ESI) calcd for $(C_{25}H_{25}NO_4S + H)^+$ 436.1583, found 436.1582; HPLC (DAICEL Chiralpak IA-H, *n*-hexane/ethanol = 7/3, flow 0.8 mL/min, detection at 240 nm) retention time = 14.7 min (major) and 19.0 min (minor), 85% ee.

(*S*)-5-((*R*)-1-(4-fluorophenyl)-2-(3-oxocyclohex-1-enyl)ethyl)-1-tosyl-1,5-dihydro-2*H*-pyrrol-2-one (4u)

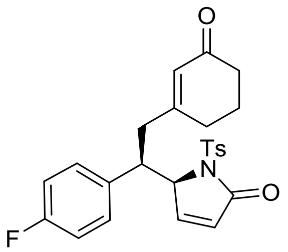

The reaction was carried out with 20 mol % of catalyst **3d** and 20 mol % of *p*-anisic acid for 4 days. The product was obtained by column chromatography (PE/AcOEt = 3/1) as a yellow oil in 45% yield. ^{1}H NMR (500 MHz, $CDCl_3$): δ 1.78-1.94 (m, 2H), 2.01-2.33 (m, 6H), 2.43 (s, 3H), 4.27 (td, *J* = 4.0, 12.0 Hz, 1H), 4.90 (“app” quint, *J* = 1.6 Hz, 1H), 5.63 (s, 1H), 6.10 (dd, *J* = 1.6, 6.4 Hz, 1H), 7.02 (dd, *J* = 2.0, 6.0 Hz, 1H), 7.06-7.12 (m, 2H), 7.27-7.30 (m, 2H), 7.33 (d, *J* = 8.0 Hz, 2H), 7.97 (d, *J* = 8.0 Hz, 2H); ^{13}C NMR (125 MHz, $CDCl_3$): δ 21.7, 22.4, 29.4, 33.6, 37.1, 43.0, 69.2, 116.3 (d, $^2J_{CF}$ = 21.4 Hz), 127.4, 127.7, 128.1, 129.7, 129.8 (d, $^3J_{CF}$ = 7.4 Hz), 133.3 (d, $^4J_{CF}$ = 3.3 Hz), 135.5, 145.5, 148.5, 161.9, 162.2 (d, $^1J_{CF}$ = 246.2 Hz), 168.9, 199.1; $[\alpha]_D^{25}$ −209 (*c* 1.00, CH_2Cl_2); HRMS (ESI) calcd for $(C_{25}H_{24}FNO_4S + NH_4)^+$ 471.1754, found 471.1752; HPLC (DAICEL Chiralpak IA-H, *n*-hexane/ethanol = 7/3, flow 0.8 mL/min, detection at 240 nm) retention time = 17.4 min (major) and 22.4 min (minor), 90% ee.

(*S*)-5-((*R*)-1-(3,4-dimethoxyphenyl)-2-(3-oxocyclohex-1-enyl)ethyl)-1-tosyl-1,5-dihydro-2*H*-pyrrol-2-one (4v)

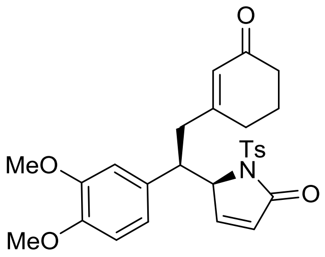

The reaction was carried out with 20 mol % of catalyst **3d** and 20 mol % of *p*-anisic acid for 4 days. The product was obtained by column chromatography (PE/AcOEt = 2/1) as a yellow oil in 67% yield. ^{1}H NMR (400 MHz, $CDCl_3$): δ 1.80-1.93 (m, 2H), 2.03-2.16 (m, 2H), 2.18- 2.35 (m, 4H), 2.44 (s, 3H), 3.88 (s, 3H), 3.92 (s, 3H), 4.16 (td, *J* = 3.2, 9.6 Hz, 1H), 4.95 (“app” quint, *J* = 1.6 Hz, 1H), 5.68 (s, 1H), 6.08 (dd, *J* = 1.6, 5.2 Hz, 1H), 6.76-6.82 (m, 2H), 6.85 (d, *J* = 6.4 Hz, 1H), 7.06 (dd, *J* = 1.6, 4.8 Hz, 1H), 7.33 (d, *J* = 8.4 Hz, 2H), 7.98 (d, *J* = 8.4 Hz, 2H); ^{13}C NMR (100 MHz, $CDCl_3$): δ 21.6, 22.4, 29.3, 33.7, 37.1, 43.2, 55.8, 56.1, 69.3, 111.5, 111.6, 119.8, 127.0, 127.5, 128.0, 129.7, 129.8, 135.5, 145.4, 148.6, 149.0, 149.4, 162.4, 168.8, 199.2; $[\alpha]_D^{25}$ −146 (*c* 1.00, CH_2Cl_2); HRMS (ESI) calcd for $(C_{27}H_{29}NO_6S + H)^+$ 496.1794, found 496.1799; HPLC (DAICEL Chiralpak IA-H, *n*-hexane/ethanol = 7/3, flow 0.8 mL/min, detection at 254 nm) retention time = 23.0 min (major) and 25.1 min (minor), 91% ee.

***tert*-Butyl (3a*R*,4*R*,5a*S*,8b*S*)-2,7-dioxo-4-phenyl-1,3a,4,5,5a,6,7,8b-octahydrocyclo penta[*e*]indole-3(2*H*)-carboxylate (6a)**

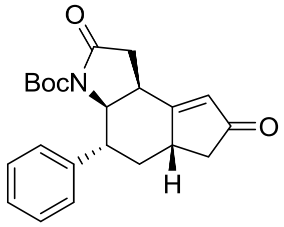

The reaction was carried out with 30 mol % of catalyst **3d** and 30 mol % of *p*-anisic acid for 4 days. The product was obtained by column chromatography (PE/AcOEt = 3/1) as a yellow oil in 51% yield. ^{1}H NMR (400 MHz, $CDCl_3$): δ 1.10 (s, 9H), 1.62-1.68 (m, 1H), 2.14 (dd, *J* = 2.0, 15.2 Hz, 1H), 2.20-2.25 (m, 1H), 2.67-2.73 (m, 2H), 2.84-2.93 (m, 2H), 3.06-3.11 (m, 1H), 3.74-3.80 (m, 1H), 4.45 (dd, *J* = 5.2, 8.4 Hz, 1H), 6.13 (d, *J* = 1.2 Hz, 1H), 7.18-7.21 (m, 2H), 7.28- 7.34 (m, 3H); ^{13}C NMR (100 MHz, $CDCl_3$): δ 27.3, 36.8, 37.6, 37.8, 38.3, 41.3, 47.1, 63.9, 83.1, 127.5, 128.1, 128.9, 130.8, 140.7, 148.6, 171.6, 178.1, 207.2; $[\alpha]_D^{25}$ −156 (*c* 1.00, CH_2Cl_2); HRMS (ESI) calcd for $(C_{22}H_{25}NO_4 + H)^+$ 368.1862, found 368.1872; HPLC (DAICEL Chiralpak IA-H, *n*-hexane/ethanol = 7/3, flow 0.8 mL/min, detection at 240 nm) retention time = 7.5 min (major) and 8.4 min (minor), 92% ee.

***tert*-Butyl (3a*R*,4*R*,5a*S*,8b*S*)-2,7-dioxo-4-(*p*-tolyl)-1,3a,4,5,5a,6,7,8b-octahydro cyclopenta[*e*]indole-3(2*H*)-carboxylate (6b)**

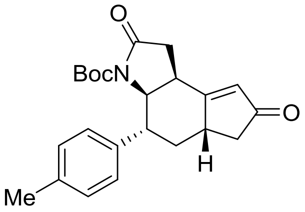

The reaction was carried out with 30 mol % of catalyst **3d** and 30 mol % of *p*-anisic acid for 4 days. The product was obtained by column chromatography (PE/AcOEt = 2/1) as a yellow oil in 45% yield. ^{1}H NMR (400 MHz, $CDCl_3$): δ 1.09 (s, 9H), 1.58-1.65 (m, 1H), 2.12 (dd, *J* = 2.4, 18.8 Hz, 1H), 2.17-2.23 (m, 1H), 2.31 (s, 3H), 2.64-2.72 (m, 2H), 2.78-2.91 (m, 2H), 3.04-3.09 (m, 1H), 3.69-3.79 (m, 1H), 4.39 (dd, *J* = 6.8, 10.4 Hz, 1H), 6.12 (d, *J* = 1.2 Hz, 1H), 7.05-7.13 (m, 4H); ^{13}C NMR (100 MHz, $CDCl_3$): δ 21.0, 27.3, 36.8, 37.5, 37.8, 38.3, 41.3, 46.7, 64.1, 83.1, 127.9, 129.6, 130.8, 137.1, 137.6, 148.6, 171.7, 178.2, 207.3; $[\alpha]_D^{25}$ −169 (*c* 1.00, CH_2Cl_2); HRMS (ESI) calcd for $(C_{23}H_{27}NO_4 + H)^+$ 382.2018, found 382.2019; HPLC (DAICEL Chiralpak AS-H, *n*-hexane/ethanol = 7/3, flow 0.7 mL/min, detection at 240 nm) retention time = 11.6 min (major) and 27.3 min (minor), 99% ee.

***tert*-Butyl (3a*R*,4*R*,5a*S*,8b*S*)-4-(4-(*tert*-butyl)phenyl)-2,7-dioxo-1,3a,4,5,5a,6,7,8b-octahydrocyclopenta[*e*]indole-3(2*H*)-carboxylate (6c)**

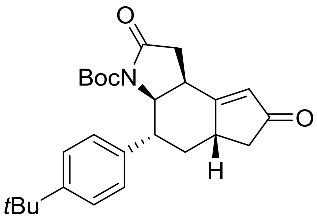

The reaction was carried out with 30 mol % of catalyst **3d** and 30 mol % of *p*-anisic acid for 4 days. The product was obtained by column chromatography (PE/AcOEt = 2/1) as a yellow oil in 67% yield. ^{1}H NMR (400 MHz, $CDCl_3$): δ 1.08 (s, 9H), 1.28 (s, 9H), 1.58-1.65 (m, 1H), 2.12 (dd, *J* = 2.4, 18.8 Hz, 1H), 2.17-2.23 (m, 1H), 2.64-2.72 (m, 2H), 2.80-2.92 (m, 2H), 3.03-3.09 (m, 1H), 3.69-3.80 (m, 1H), 4.43 (dd, *J* = 6.8, 10.4 Hz, 1H), 6.12 (d, *J* = 1.2 Hz, 1H), 7.10 (d, *J* = 8.4 Hz, 2H), 7.32 (d, *J* = 8.4 Hz, 2H); ^{13}C NMR (100 MHz, $CDCl_3$): δ 27.4, 31.3, 34.5, 36.8, 37.7, 37.8, 38.3, 41.3, 46.6, 63.9, 83.0, 125.9, 127.7, 130.7, 137.6, 148.7, 150.3, 171.7, 178.3, 207.3; $[\alpha]_D^{25}$ −43.6 (*c* 1.00, CH_2Cl_2); HRMS (ESI) calcd for $(C_{26}H_{33}NO_4 + H)^+$ 424.2488, found 424.2485; HPLC

(DAICEL Chiralpak IA-H, *n*-hexane/ethanol = 7/3, flow 0.8 mL/min, detection at 240 nm) retention time = 11.9 min (major) and 16.7 min (minor), 99% ee.

***tert*-Butyl (3a*R*,4*R*,5a*S*,8b*S*)-4-(naphthalen-2-yl)-2,7-dioxo-1,3a,4,5,5a,6,7,8b-octahydrocyclopenta[*e*]indole-3(2*H*)-carboxylate (6d)**

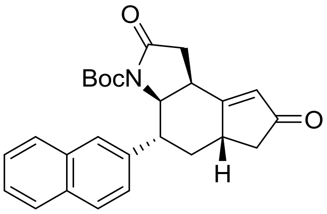

The reaction was carried out with 30 mol % of catalyst **3d** and 30 mol % of *p*-anisic acid for 4 days. The product was obtained by column chromatography (PE/AcOEt = 2/1) as a white solid in 65% yield. M.p.: 166-167 °C; ^{1}H NMR (400 MHz, $CDCl_3$): δ 0.80 (s, 9H), 1.78 (dd, *J* = 12.8, 26.0 Hz, 1H), 2.16 (dd, *J* = 2.0, 19.2 Hz, 1H), 2.25-2.31 (m, 1H), 2.68-2.75 (m, 2H), 2.93-3.14 (m, 3H), 3.75-3.83 (m, 1H), 4.52 (dd, *J* = 6.8, 10.4 Hz, 1H), 6.15 (d, *J* = 1.2 Hz, 1H), 7.33 (dd, *J* = 1.6, 8.4 Hz, 1H), 7.43-7.47 (m, 2H), 7.63 (s, 1H), 7.74-7.83 (m, 3H); ^{13}C NMR (100 MHz, $CDCl_3$): δ 27.0, 36.8, 37.4, 37.8, 38.2, 41.3, 47.3, 63.8, 83.1, 125.4, 126.0, 126.4, 127.1, 127.5, 127.6, 128.7, 130.9, 132.8, 133.5, 137.9, 148.6, 171.7, 178.1, 207.2; $[\alpha]_D^{25}$ −154 (*c* 1.00, CH_2Cl_2); HRMS (ESI) calcd for $(C_{26}H_{27}NO_4 + Na)^+$ 440.1838, found 440.1835; HPLC (DAICEL Chiralpak AD-H, *n*-hexane/ethanol = 7/3, flow 0.8 mL/min, detection at 240 nm) retention time = 15.4 min (minor) and 17.0 min (major), 97% ee.

***tert*-Butyl (3a*S*,4*R*,5a*S*,8b*S*)-2,7-dioxo-4-(thiophen-3-yl)-1,3a,4,5,5a,6,7,8b-octahydrocyclopenta[*e*]indole-3(2*H*)-carboxylate (6e)**

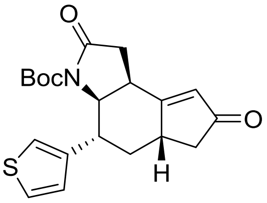

The reaction was carried out with 30 mol % of catalyst **3d** and 30 mol % of *p*-anisic acid for 4 days. The product was obtained by column chromatography (PE/AcOEt = 3/1) as a yellow oil in 40% yield. ^{1}H NMR (400 MHz, $CDCl_3$): δ 1.23 (s, 9H), 1.54-1.59 (m, 1H), 2.13 (dd, *J* = 2.4, 18.8 Hz, 1H), 2.22-2.28 (m, 1H), 2.65-2.73 (m, 2H), 2.86 (dd, *J* = 13.2, 17.2 Hz, 1H), 3.00-3.08 (m, 2H), 3.71-3.79 (m, 1H), 4.40 (dd, *J* = 6.4, 10.4 Hz, 1H), 6.13 (d, *J* = 1.6 Hz, 1H), 6.96-6.98 (m, 1H), 7.03-7.05 (m, 1H), 7.29-7.32 (m, 1H); ^{13}C NMR (100 MHz, $CDCl_3$): δ 27.6, 36.8, 37.6, 37.7, 38.2, 41.2, 42.4, 63.5, 83.2, 121.8, 126.4, 126.5, 130.8, 141.4, 148.8, 171.5, 177.9, 207.1; $[\alpha]_D^{25}$ −122 (*c* 1.00, CH_2Cl_2); HRMS (ESI) calcd for $(C_{20}H_{23}NO_4S + NH_4)^+$ 391.1692, found 391.1696; HPLC (DAICEL Chiralpak IA-H, *n*-hexane/ethanol = 9/1, flow 1.0 mL/min, detection at 240 nm) retention time = 22.3 min (major) and 27.4 min (minor), 98% ee.

Benzyl (3a*R*,4*R*,5a*S*,8b*S*)-2,7-dioxo-4-phenyl-1,3a,4,5,5a,6,7,8b-octahydrocyclopenta[*e*]indole-3(2*H*)-carboxylate (6f)

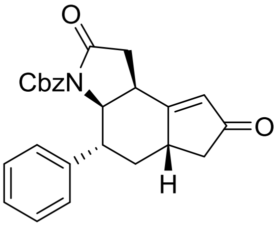

The reaction was carried out with 30 mol % of catalyst **3d** and 30 mol % of *p*-anisic acid for 4 days. The product was obtained by column chromatography (PE/AcOEt = 2/1) as a yellow oil in 37% yield. ^{1}H NMR (400 MHz, $CDCl_3$): δ 1.62-1.73 (m, 1H), 2.14 (dd, *J* = 2.4, 18.8 Hz, 1H), 2.22-2.28 (m, 1H), 2.67-2.75 (m, 2H), 2.86-2.95 (m, 2H), 3.07-3.11 (m, 1H), 3.73-3.80 (m, 1H), 4.08 (d, *J* = 12.4 Hz, 1H), 4.48 (dd, *J* = 6.8, 10.4 Hz, 1H), 4.93 (d, *J* = 12.4 Hz,

1H), 6.12 (s, 1H), 7.12-7.18 (m, 4H), 7.22-7.25 (m, 2H), 7.27-7.31 (m, 4H); ^{13}C NMR (100 MHz, $CDCl_3$): δ 36.5, 37.1, 37.7, 38.0, 41.3, 47.2, 64.2, 67.8, 127.6, 127.8, 128.4, 128.4, 128.4, 128.8, 131.0, 134.8, 140.5, 150.1, 171.4, 177.5, 207.1; $[\alpha]_D^{25}$ −116 (*c* 1.00, CH_2Cl_2); HRMS (ESI) calcd for $(C_{25}H_{23}NO_4 + NH_4)^+$ 419.1971, found 419.1969; HPLC (DAICEL Chiralpak IA-H, *n*-hexane/ethanol = 7/3, flow 0.8 mL/min, detection at 240 nm) retention time = 11.2 (major en B), 13.1 min (minor en B), 93% ee.

***tert*-Butyl 2-oxo-4-(4-oxo-2-phenylcyclopent-2-enyl)pyrrolidine-1-carboxylate (8a)**

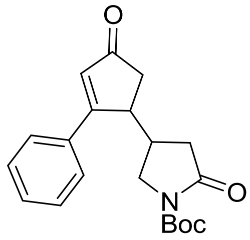

Catalyst **3d** (0.06 mmol, 0.3 equiv), *p*-anisic acid (0.12 mmol, 0.6 equiv) and *N*-Boc α,β-unsaturated γ-butyrolactam **2a** (0.2 mmol, 1 equiv) were added in sequence to a solution of enone **7a** (0.6 mmol, 3 equiv) in DCM (0.4 mL). The reaction mixture was stirred at 40 ℃ for 3 days, then the solvent was removed *in vacuo*. The residue was purified by flash silica gel chromatography (PE/AcOEt = 2/1) to obtain the product as a yellow oil in 43% yield and 1:1 dr (ee was not measured). Diastereoisomer 1 (less polar): ^{1}H NMR (400 MHz, $CDCl_3$): δ 1.48 (s, 9H), 2.08 (d, *J* = 10.0 Hz, 2H), 2.34 (d, *J* = 18.8 Hz, 1H), 2.70 (dd, *J* = 6.8, 18.8 Hz, 2H), 3.41 (dd, *J* = 9.2, 10.8 Hz, 1H), 3.71-3.74 (m, 1H), 3.82-3.88 (m, 1H), 6.43 (s, 1H), 7.47-7.50 (m, 5H); ^{13}C NMR (100 MHz, $CDCl_3$): δ 28.0, 32.4, 33.5, 37.4, 41.5, 50.2, 83.2, 128.8, 129.3, 130.4, 131.2, 133.3, 149.7, 172.1, 176.1, 206.1; HRMS (ESI) calcd for $(C_{20}H_{23}NO_4 + Na)^+$ 364.1525, found 364.1527; Diastereoisomer 2: ^{1}H NMR 1.47 (s, 9H), 2.34-2.41 (m, 2H), 2.54-2.61 (m, 1H), 2.68-2.76 (m, 2H), 3.15 (dd, *J* = 8.8, 11.2 Hz, 1H), 3.41 (m, *J* = 8.4, 11.2 Hz, 1H), 3.73-3.77 (m, 1H), 6.47 (d, *J* = 0.8 Hz, 1H), 7.48-7.54 (m, 5H); ^{13}C NMR (100 MHz, $CDCl_3$): δ 28.0, 31.9, 37.6, 37.8, 42.0, 46.6, 83.4, 126.8, 129.3, 130.7, 131.3, 133.3, 172.2, 149.8, 172.2, 175.6, 206.1; HRMS (ESI) calcd for $(C_{20}H_{23}NO_4 + Na)^+$ 364.1525, found 364.1528.

D. Determination of Absolute Configuration

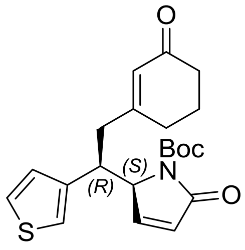

The absolute configuration of compound **4p** was unambiguously assigned by single crystal X-ray analysis. Crystals of **4p** were obtained by slow evaporation of a mixture of *n*-pentane/ethyl acetate at room temperature.

Crystal data for **4p**: **CCDC** 1020721

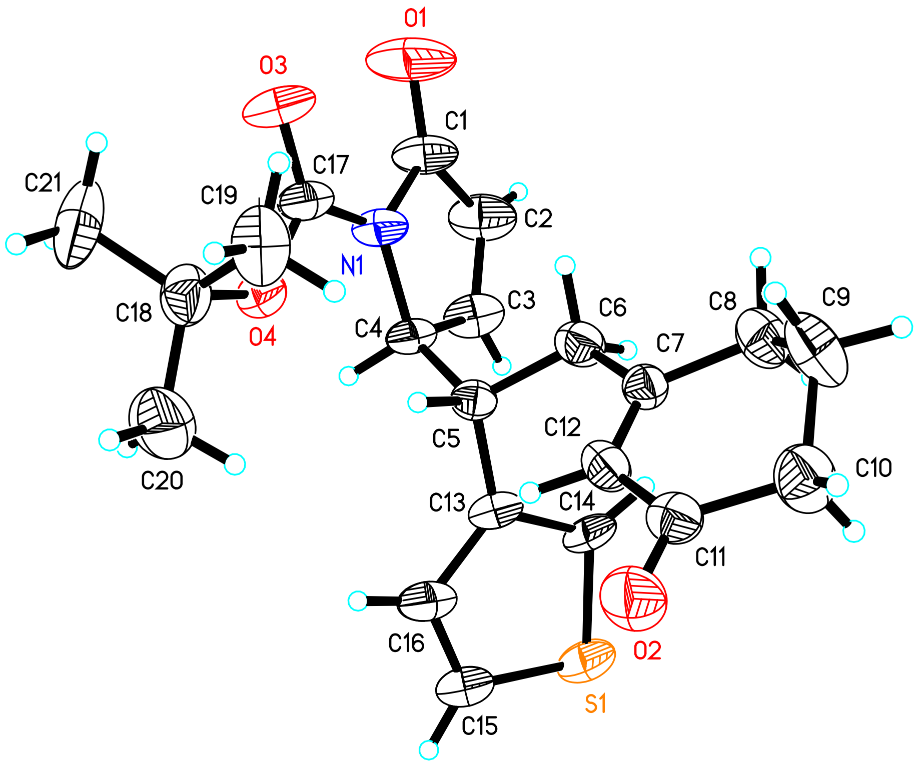

Table S7. Crystal data and structure refinement for 1020721.

Identification code	1020721	
Empirical formula	C21 H25 N O4 S	
Formula weight	387.48	
Temperature	293(2) K	
Wavelength	0.71073 Å	
Crystal system	Monoclinic	
Space group	P 21	
Unit cell dimensions	a = 8.7073(17) Å	α=90°.
	b = 10.752(2) Å	β=96.227(5)°.
	c = 10.999(2) Å	γ =90°.
Volume	1023.7(4) $Å^3$	
Z	2	
Density (calculated)	1.257 Mg/m^3	
Absorption coefficient	0.183 mm^{-1}	
F(000)	412	
Crystal size	0.211 x 0.165 x 0.123 mm	
Theta range for data collection	1.862 to 25.496°.	
Index ranges	-10<=h<=7, -12<=k<=13, -13<=l<=13	
Reflections collected	5701	
Independent reflections	3612 [R(int) = 0.0411]	
Completeness to theta = 25.242°	99.5 %	
Absorption correction	Semi-empirical from equivalents	
Max. and min. transmission	0.7457 and 0.5943	
Refinement method	Full-matrix least-squares on F^2	
Data / restraints / parameters	3612 / 74 / 297	
Goodness-of-fit on F^2	1.189	
Final R indices [I>2sigma(I)]	R_1 = 0.0761, wR_2 = 0.2206	
R indices (all data)	R_1 = 0.1038, wR_2 = 0.3025	
Absolute structure parameter	0.06(15)	
Extinction coefficient	0.016(16)	
Largest diff. peak and hole	0.340 and -0.317 e.$Å^{-3}$	

Crystals of compound **6d** were obtained by slow evaporation of a mixture of *n*-hexane/dichloromethane at room temperature.

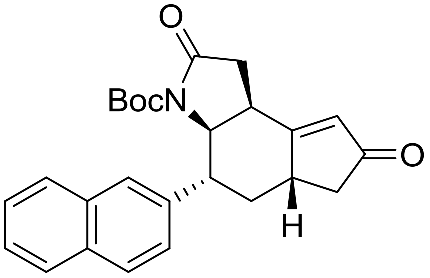

Crystal data for **6d**: **CCDC** 1020722

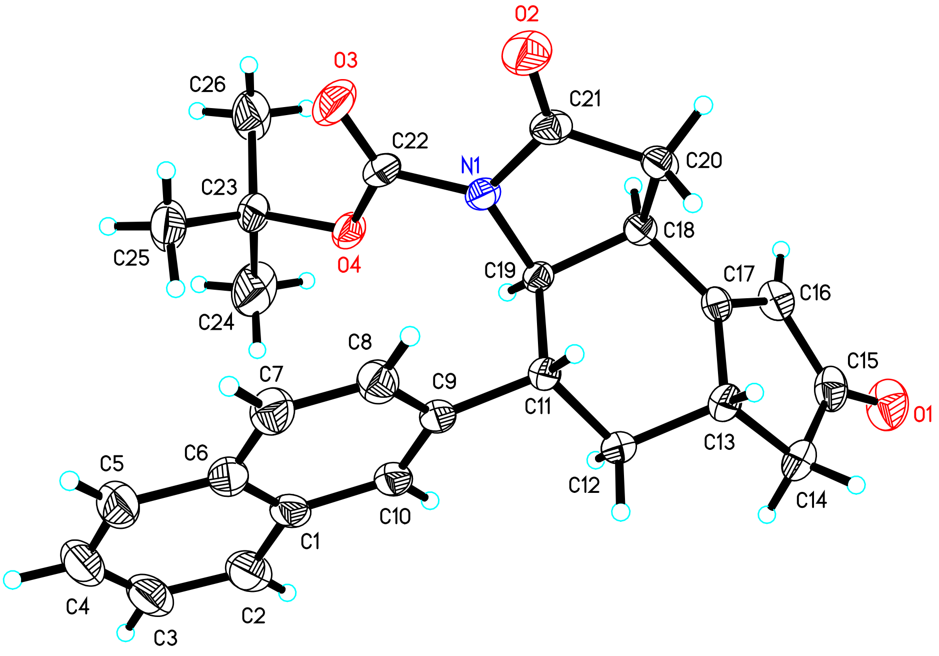

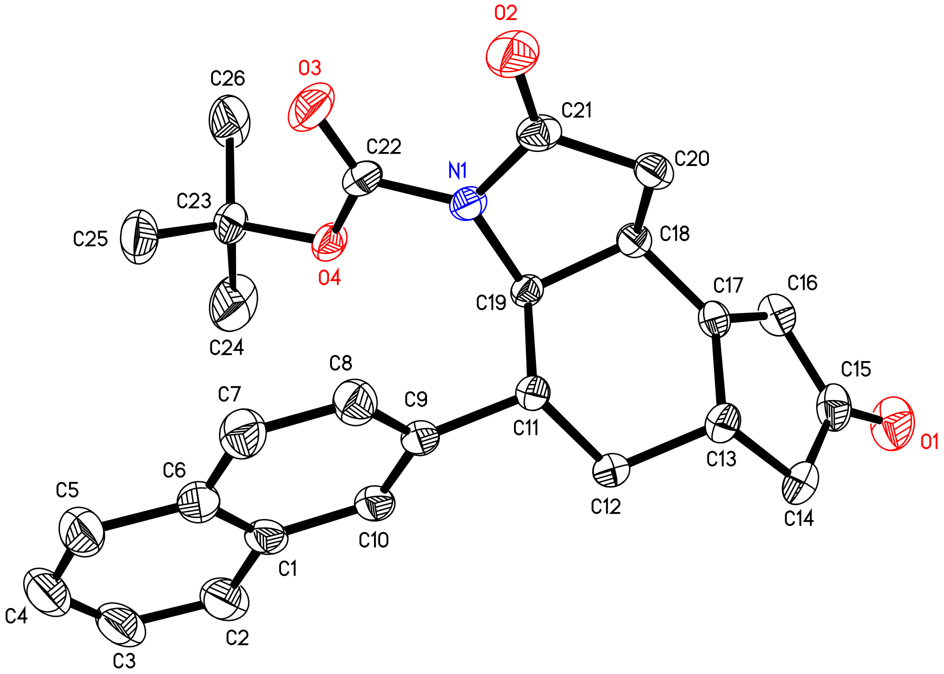
O2
O3
C26
C21
C22
C20
N1
C23
C25
C18
O4
C17
C16
C19
C24
C15
C8
C7
C9
C11
O1
C13
C5
C6
C12
C10
C14
C1
C4
C2
C3

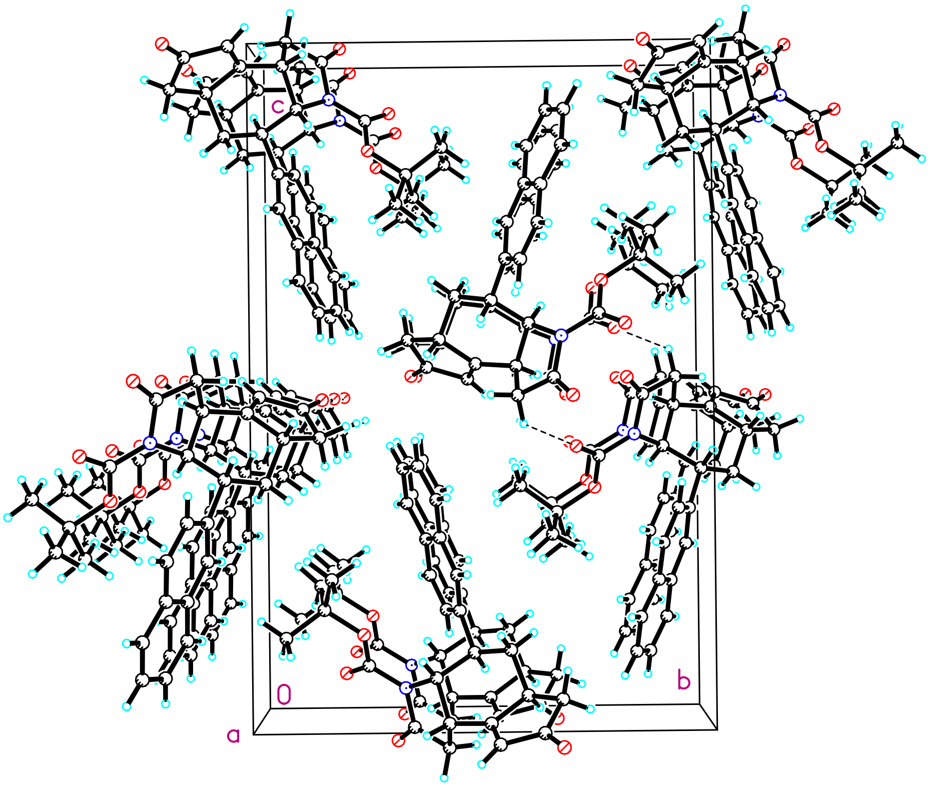
c
0
b
a

Table S9. Crystal data and structure refinement for 1020722.

Identification code	1020722	
Empirical formula	C26 H27 N O4	
Formula weight	417.48	
Temperature	293(2) K	
Wavelength	0.71073 Å	
Crystal system	Orthorhombic	
Space group	P 21 21 21	
Unit cell dimensions	a = 6.560(3) Å	α= 90 °.
	b = 15.009(7) Å	β= 90 °.
	c = 22.380(10) Å	γ= 90 °.
Volume	2203.5(18) $Å^3$	
Z	4	
Density (calculated)	1.258 Mg/m^3	
Absorption coefficient	0.084 mm^{-1}	
F(000)	888	
Crystal size	0.175 x 0.148 x 0.111 mm	
Theta range for data collection	1.634 to 25.989 °.	
Index ranges	-8<=h<=8, -16<=k<=18, -27<=l<=20	
Reflections collected	13213	
Independent reflections	4312 [R(int) = 0.0457]	
Completeness to theta = 25.242 °	100.0 %	
Absorption correction	Semi-empirical from equivalents	
Max. and min. transmission	0.7457 and 0.6614	
Refinement method	Full-matrix least-squares on F^2	
Data / restraints / parameters	4312 / 0 / 283	
Goodness-of-fit on F^2	1.038	
Final R indices [I>2sigma(I)]	R_1 = 0.0503, wR_2 = 0.1268	
R indices (all data)	R_1 = 0.0602, wR_2 = 0.1319	
Absolute structure parameter	0.8(7)	
Extinction coefficient	n/a	
Largest diff. peak and hole	0.274 and -0.141 e.$Å^{-3}$	

E: NMR Spectra of Doubly Vinylogous Michael Adducts and Derivatives

***tert*-Butyl (*S*)-2-oxo-5-((*R*)-2-(3-oxocyclohex-1-enyl)-1-phenylethyl)-2,5-dihydro-1*H*-pyrrole-1-carboxylate (4a)**

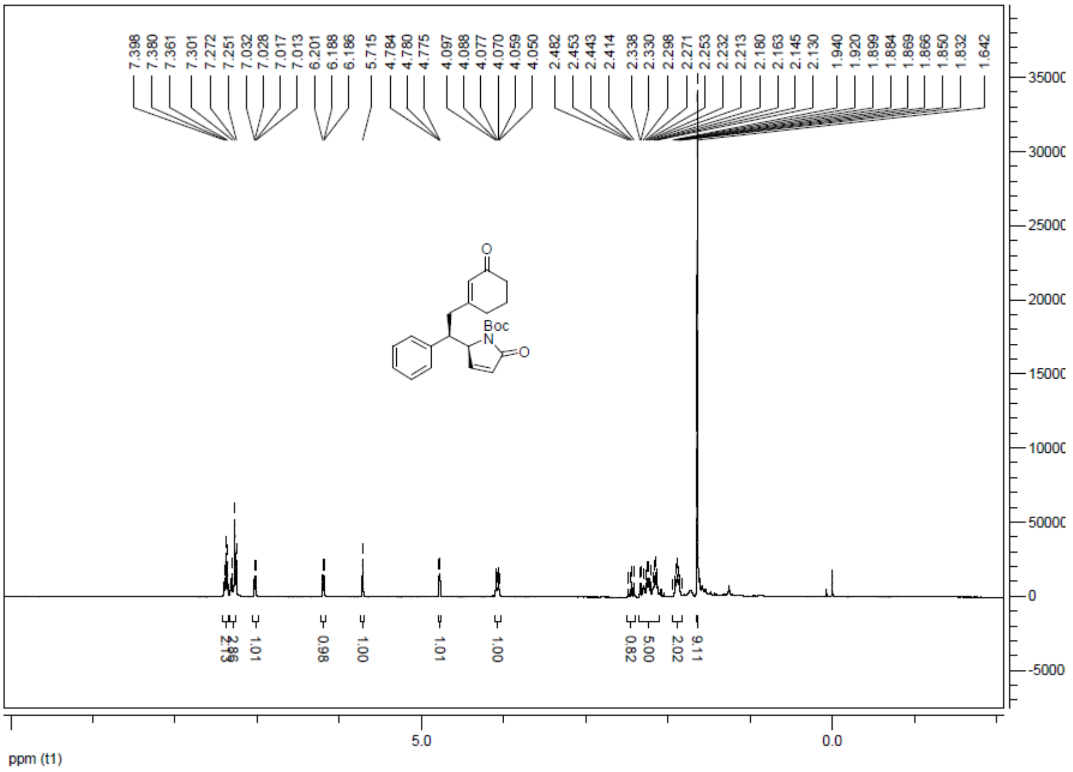

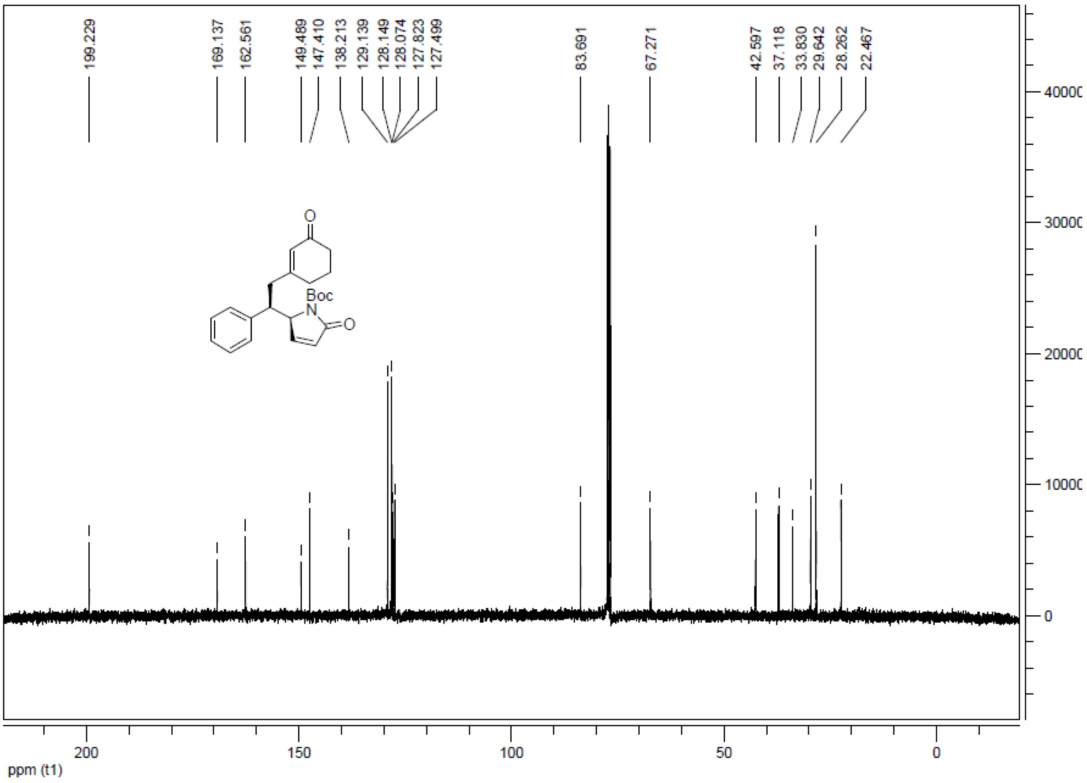

***tert*-Butyl (*S*)-2-oxo-5-((*R*)-2-(3-oxocyclohex-1-enyl)-1-*p*-tolylethyl)-2,5-dihydro-1*H*-pyrrole-1-carboxylate (4b)**

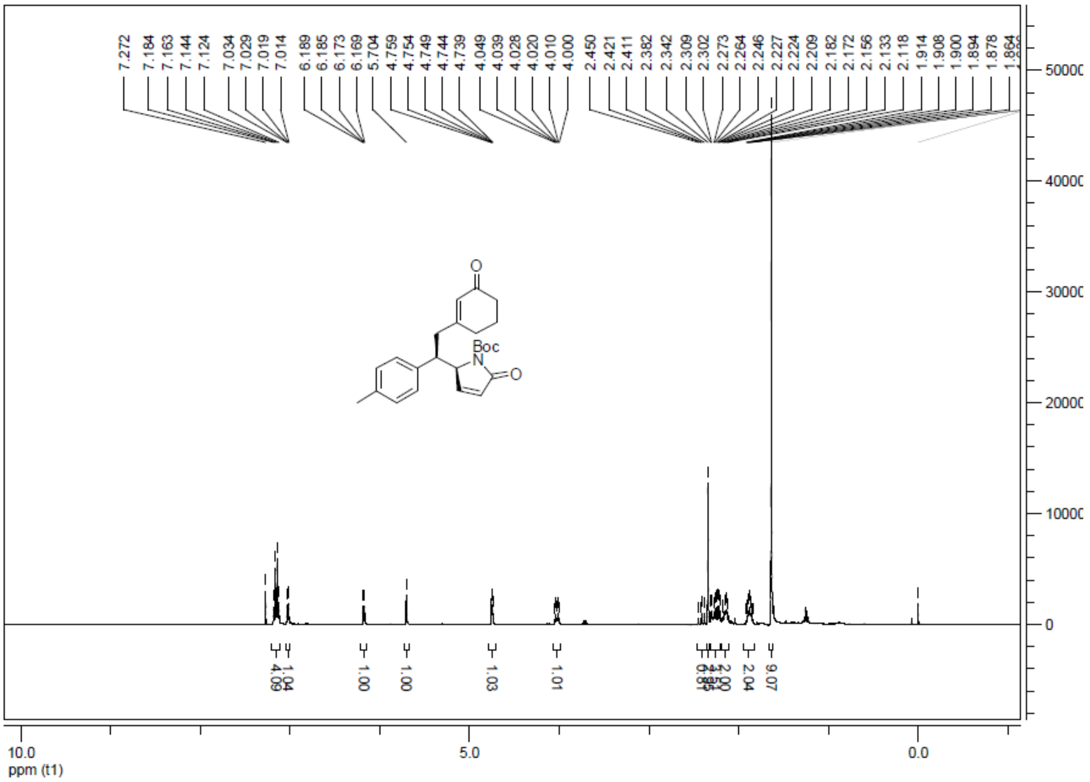

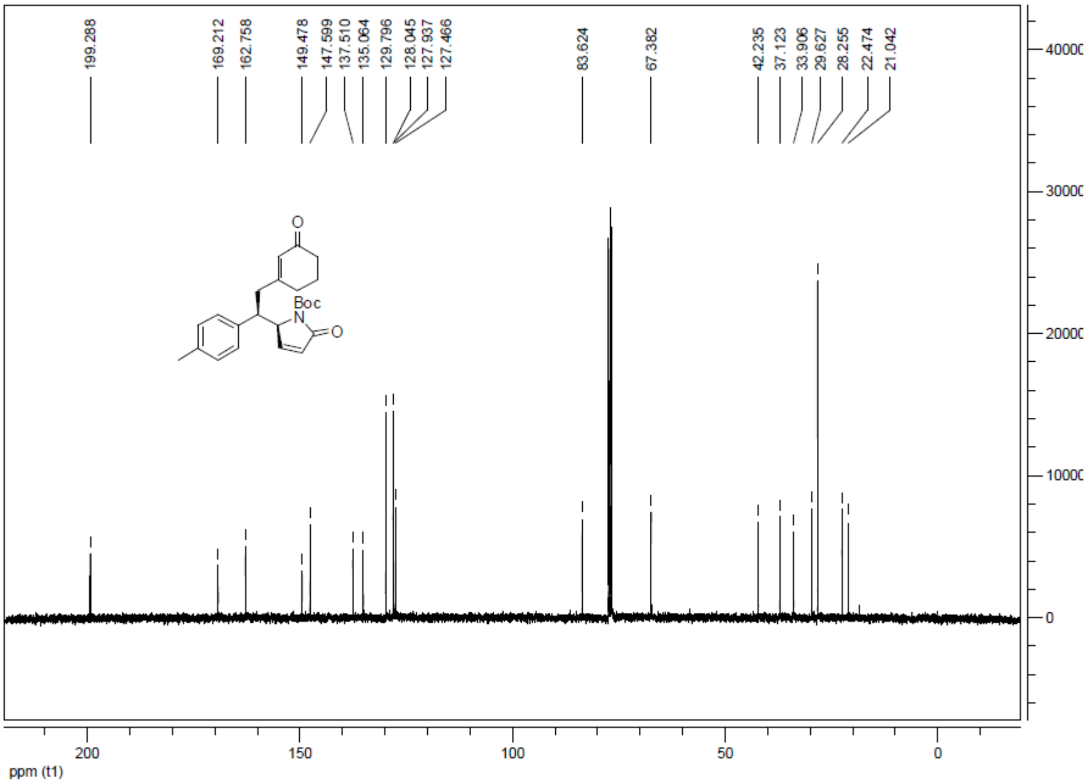

tert-Butyl (S)-2-oxo-5-((R)-2-(3-oxocyclohex-1-enyl)-4-tert-butylphenyl)-2,5-dihydro- 1H-pyrrole-1-carboxylate (4c)

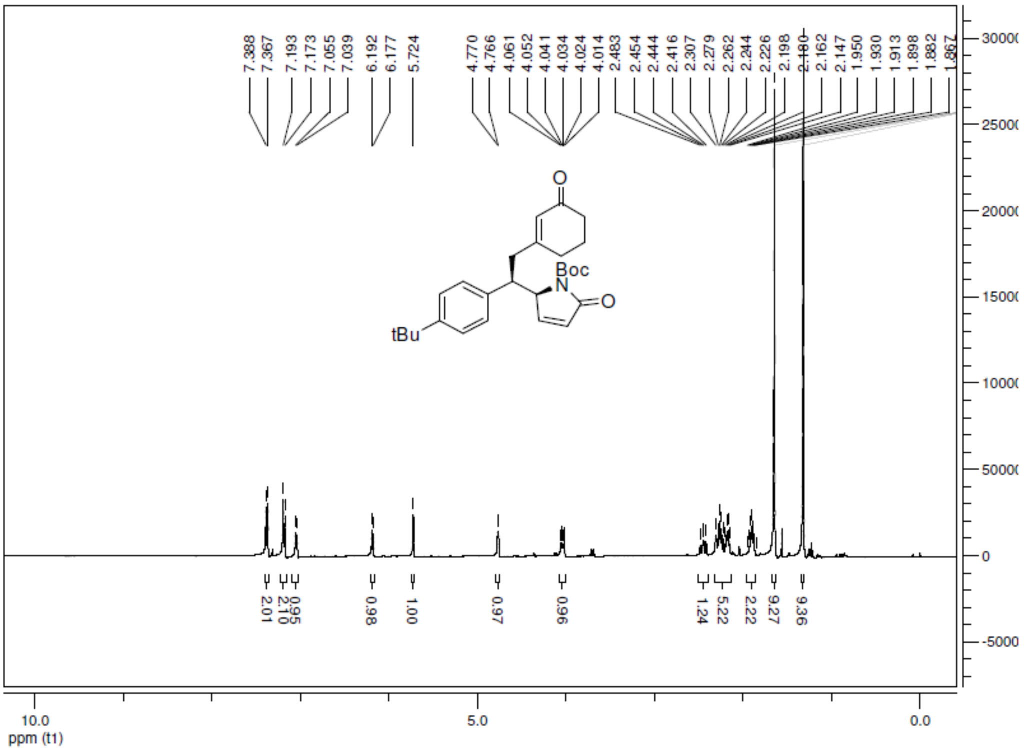

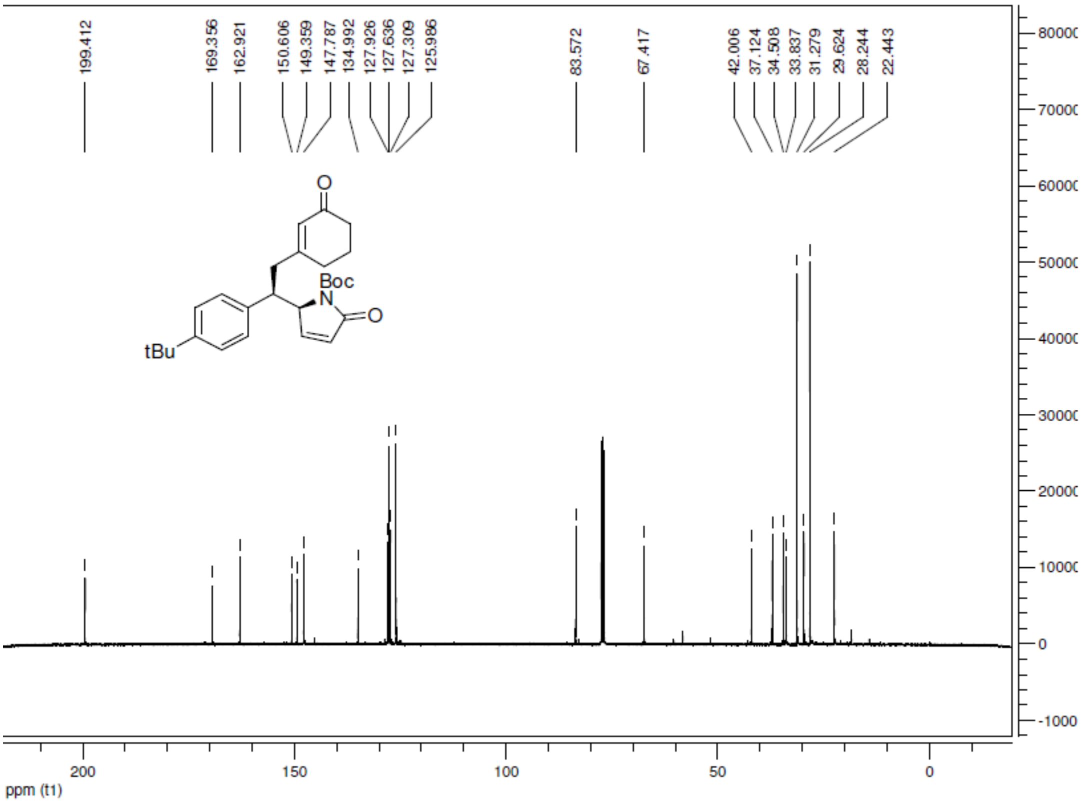

tert-Butyl (S)-2-oxo-5-((R)-2-(3-oxocyclohex-1-enyl)-1-o-tolylethyl)-2,5-dihydro-

1*H*-pyrrole-1-carboxylate (4d)

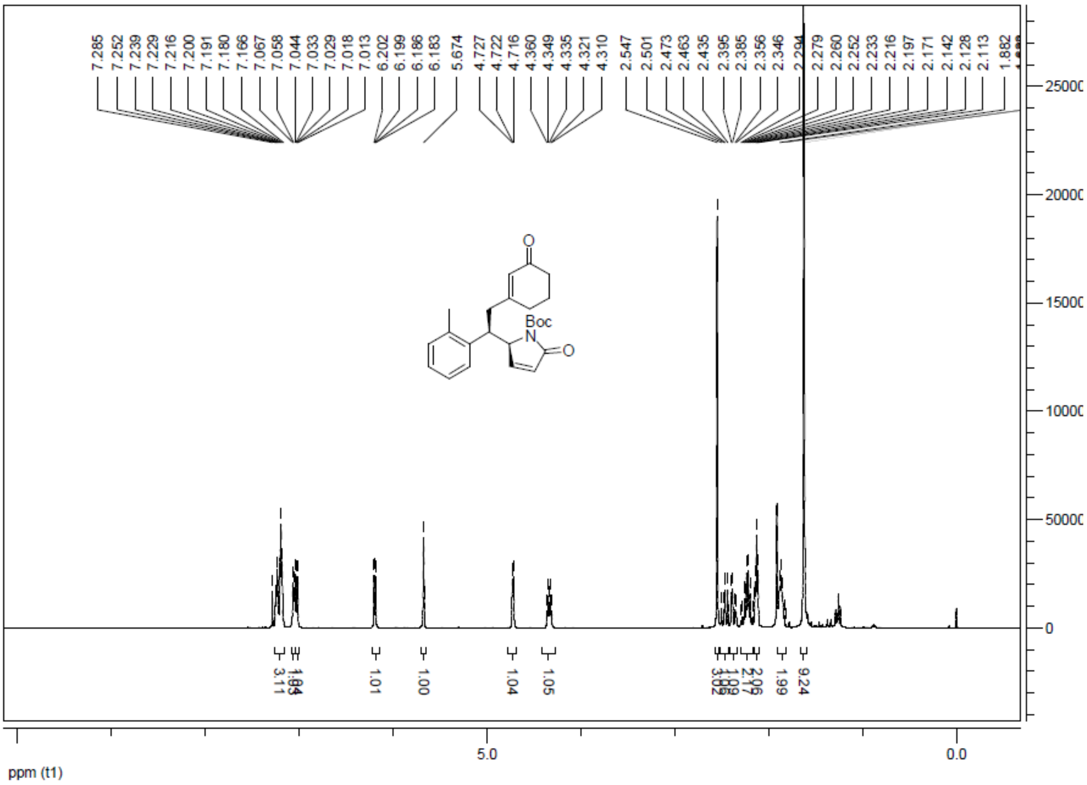

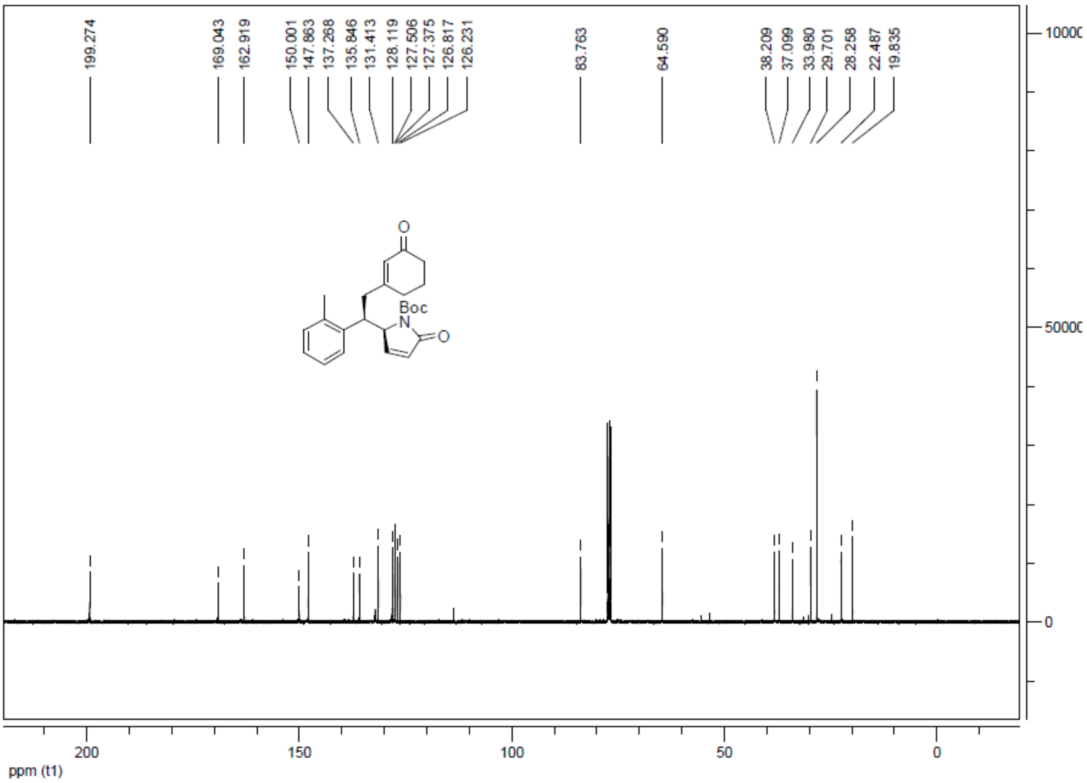

***tert*-Butyl (*S*)-2-oxo-5-((*R*)-2-(3-oxocyclohex-1-en-1-yl)-1-(2-(pivaloyloxy)phenyl) ethyl)-2,5-dihydro-1*H*-pyrrole-1-carboxylate (4e)**

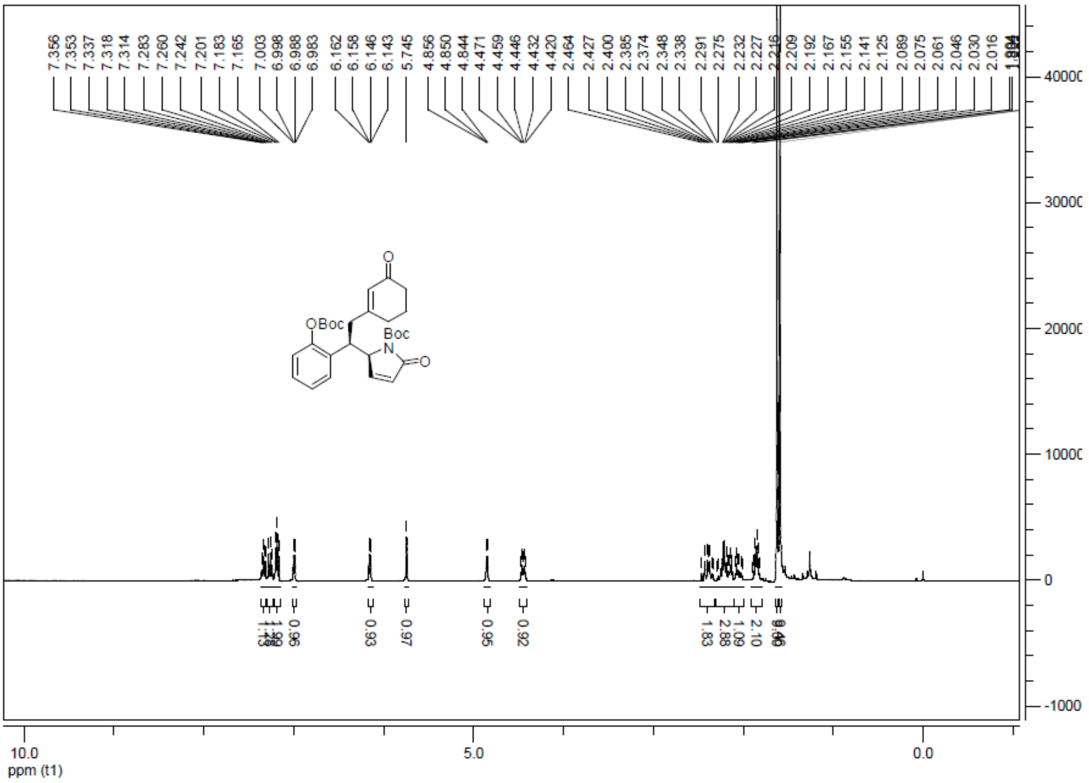

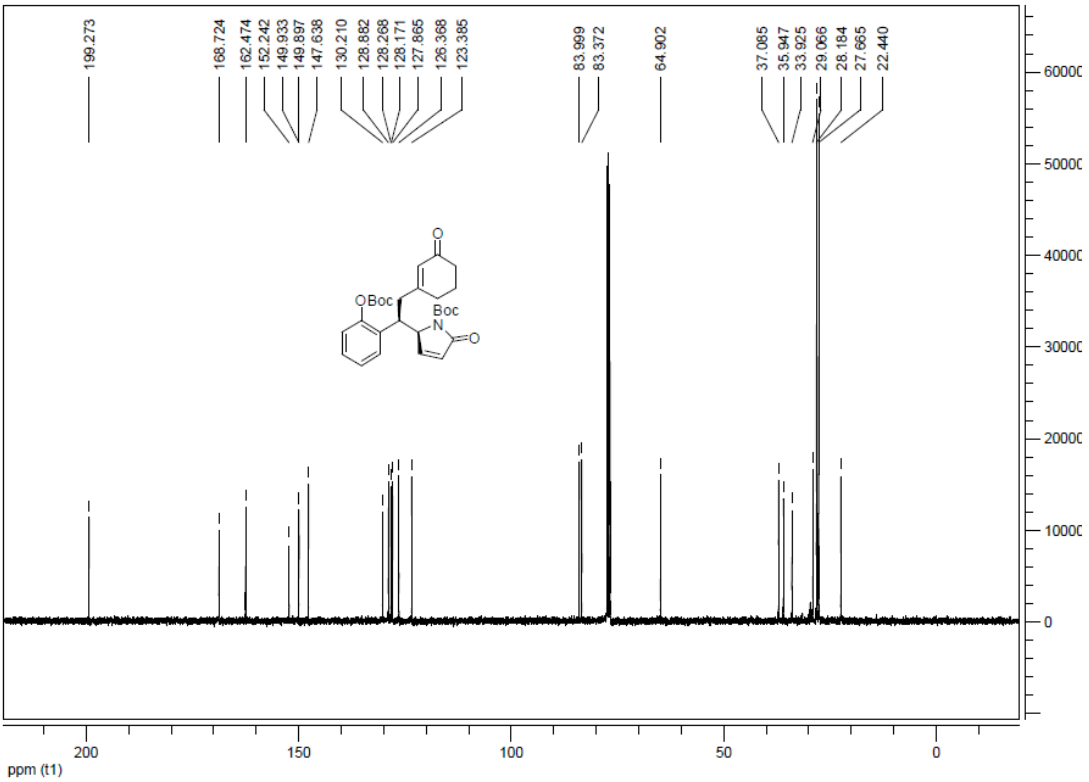

***tert*-Butyl (*S*)-2-((*R*)-1-(3,4-dimethoxyphenyl)-2-(3-oxocyclohex-1-en-1-yl)ethyl)-5-oxo-2,5-dihydro-1*H*-pyrrole-1-carboxylate (4f)**

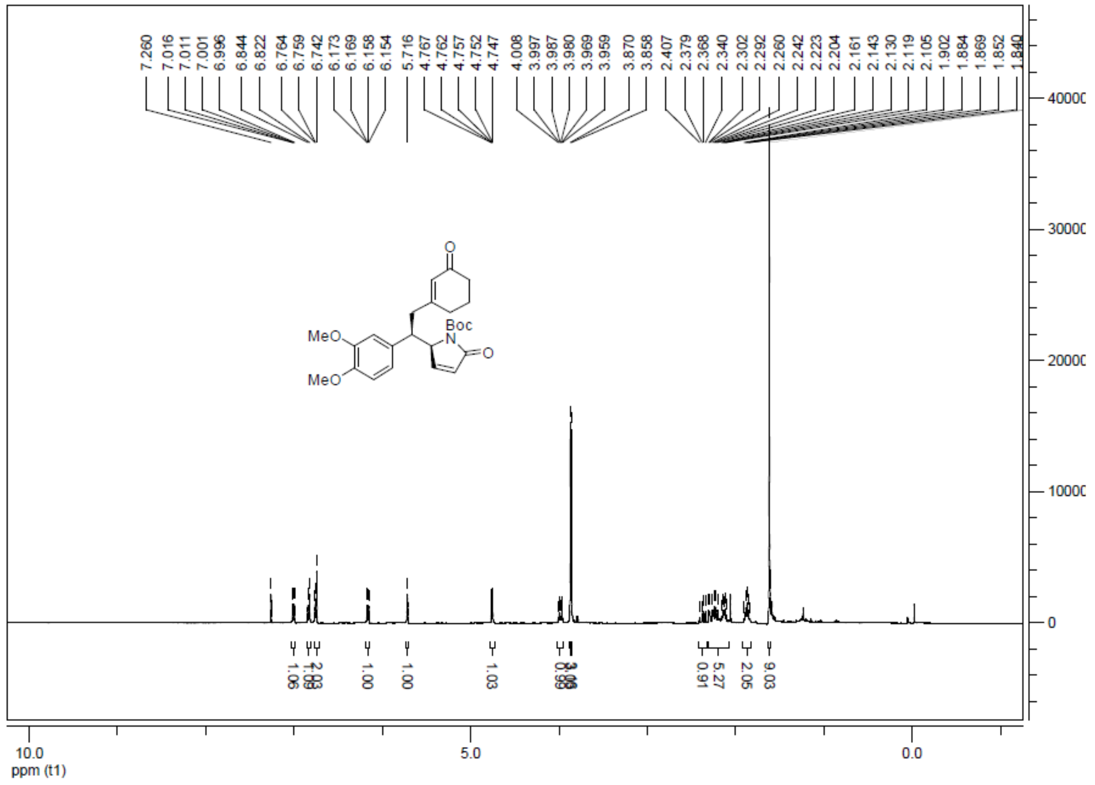

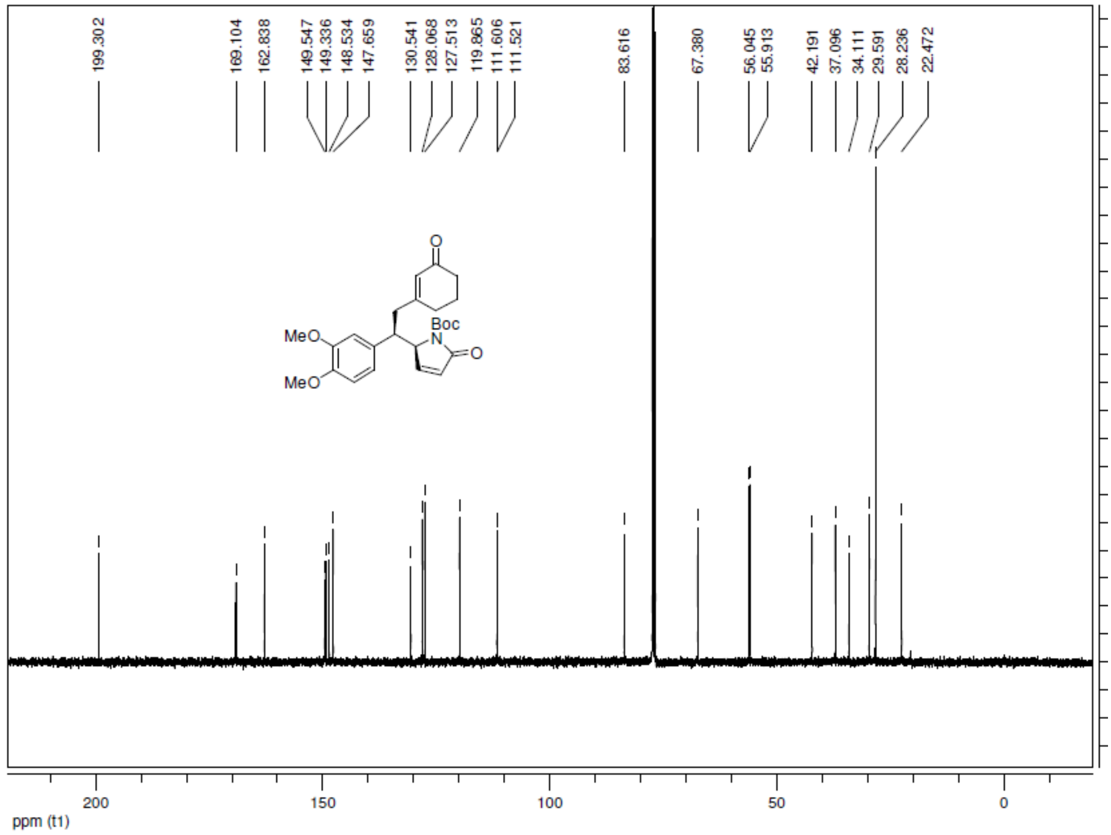

***tert*-Butyl (*S*)-2-((*R*)-1-(4-nitrophenyl)-2-(3-oxocyclohex-1-enyl)ethyl)-5-oxo- 2,5-dihydro-1*H*-pyrrole-1-carboxylate (4g)**

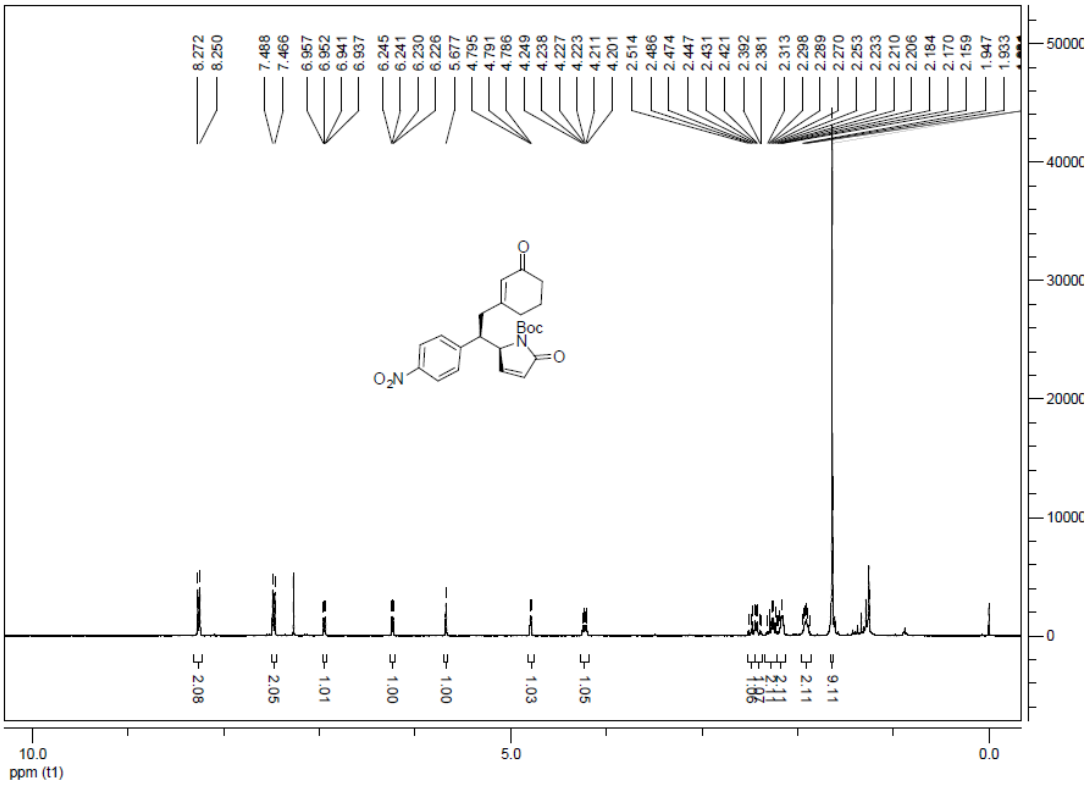

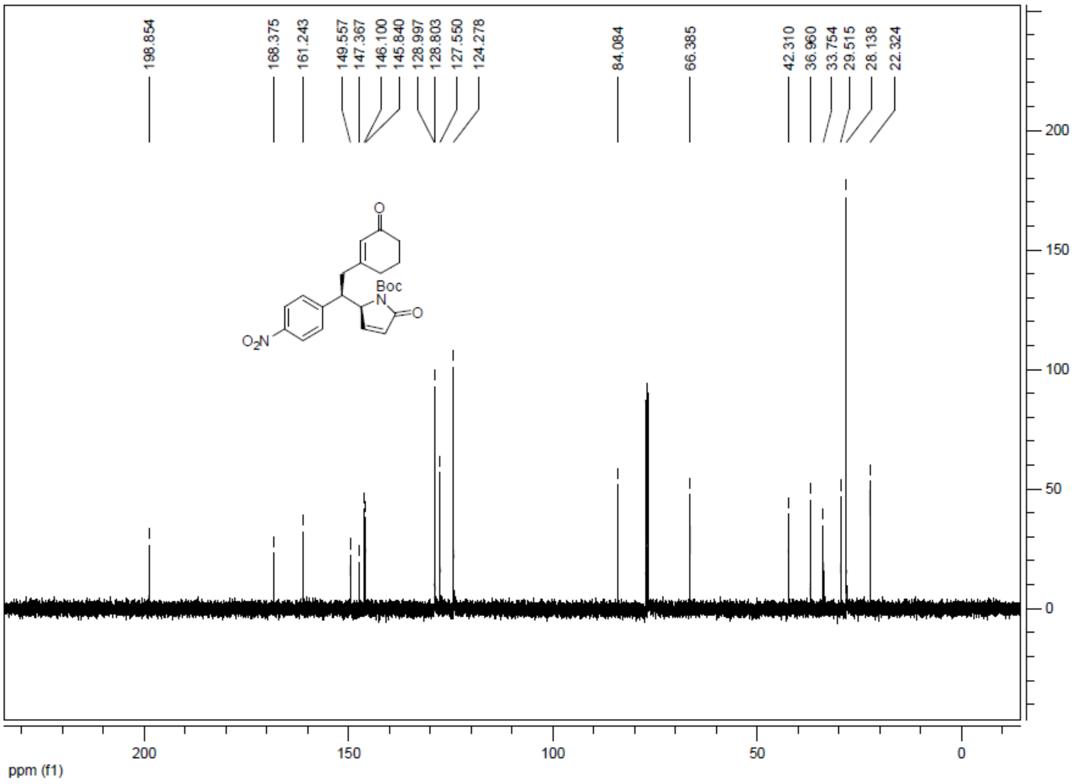

***tert*-Butyl (*S*)-2-((*R*)-1-(3-bromophenyl)-2-(3-oxocyclohex-1-enyl)ethyl)-5-oxo-2,5-dihydro-1*H*-pyrrole-1-carboxylate (4h)**

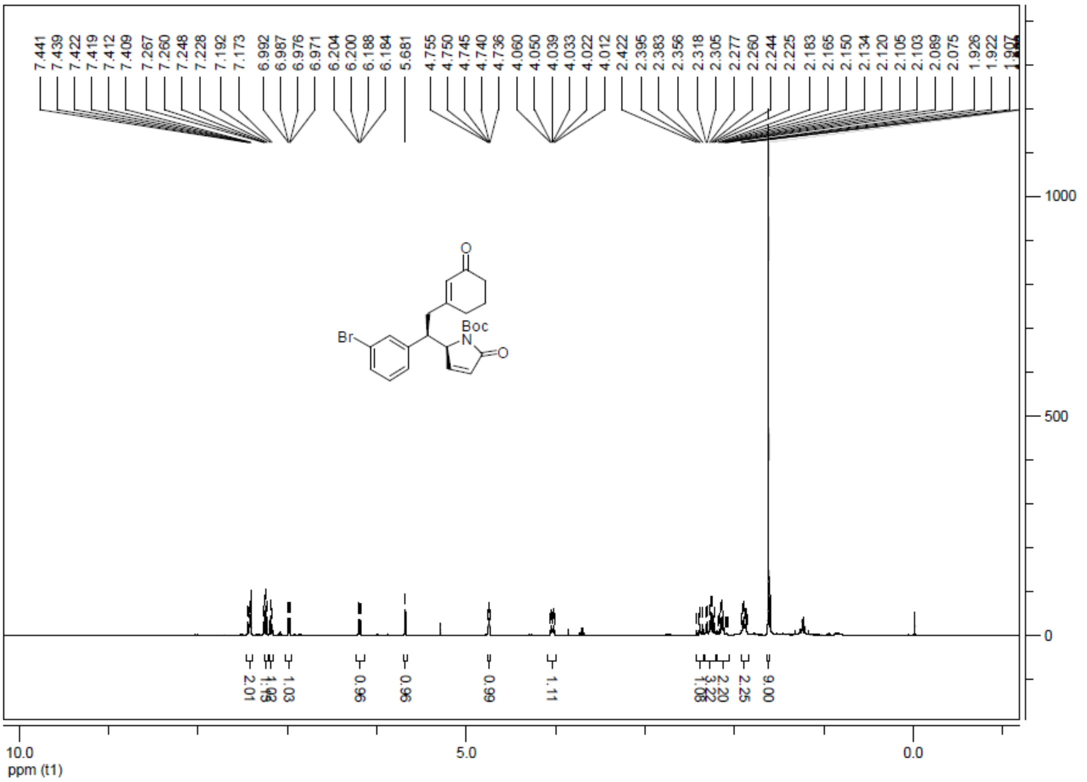

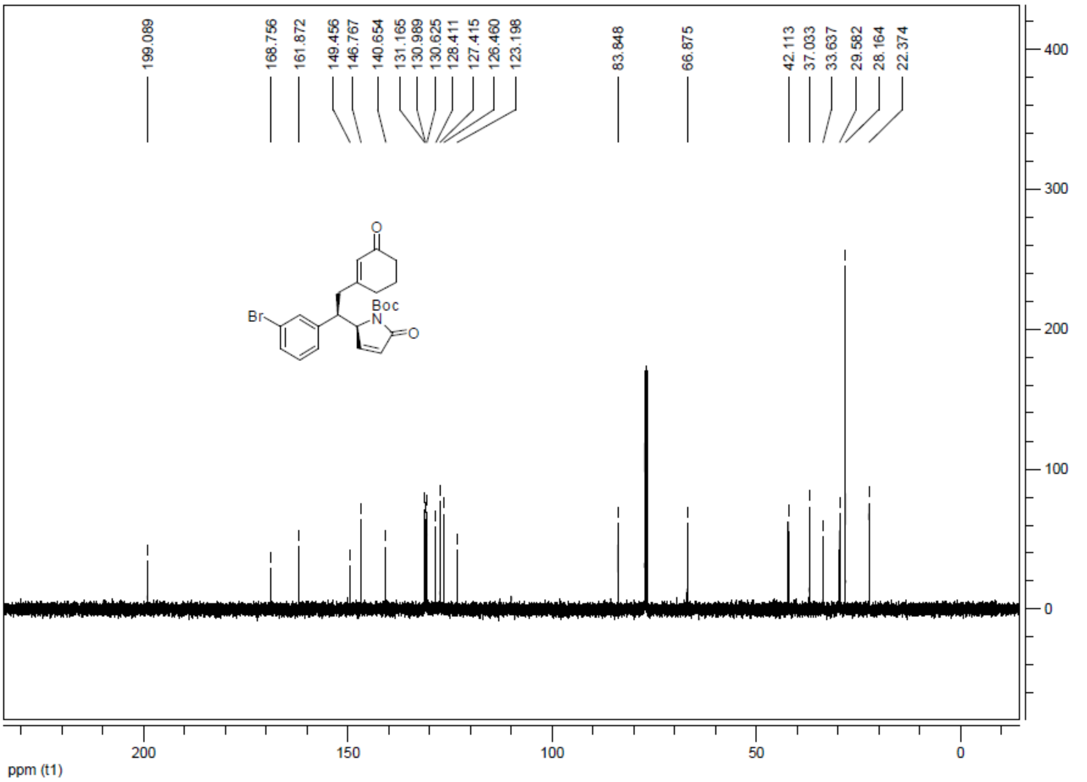

***tert*-Butyl (*S*)-2-((*R*)-1-(4-fluorophenyl)-2-(3-oxocyclohex-1-enyl)ethyl)-5-oxo- 2,5-dihydro-1*H*-pyrrole-1-carboxylate (4i)**

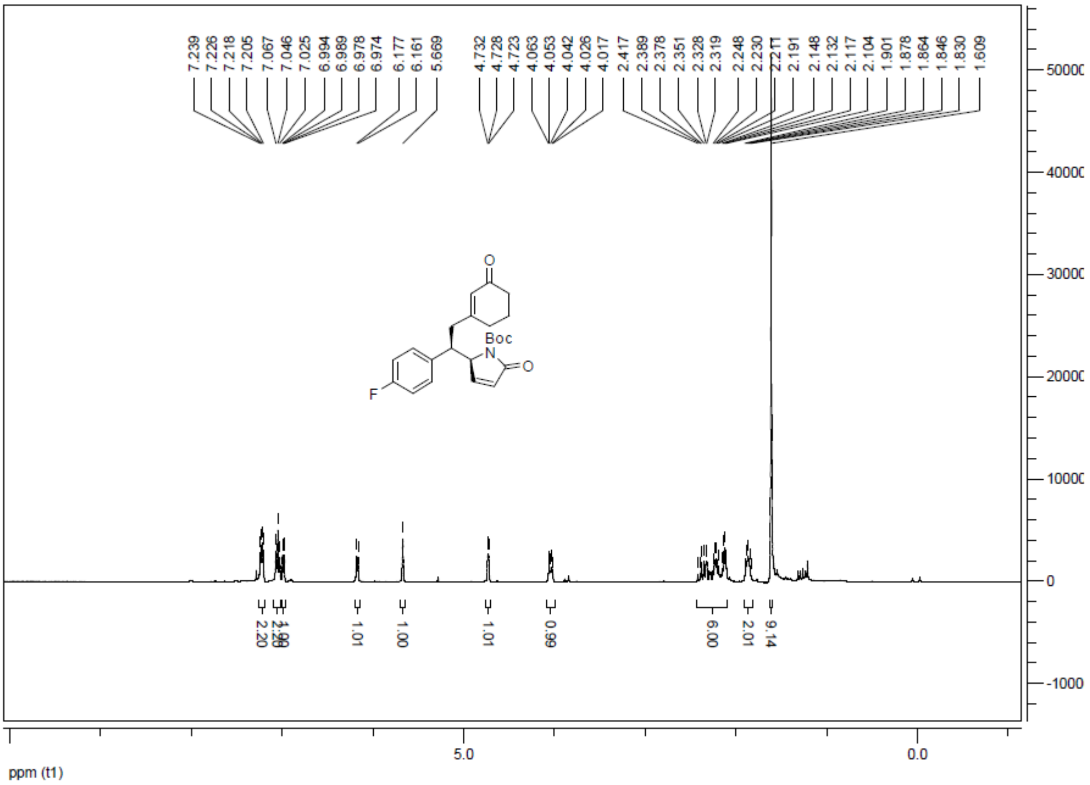

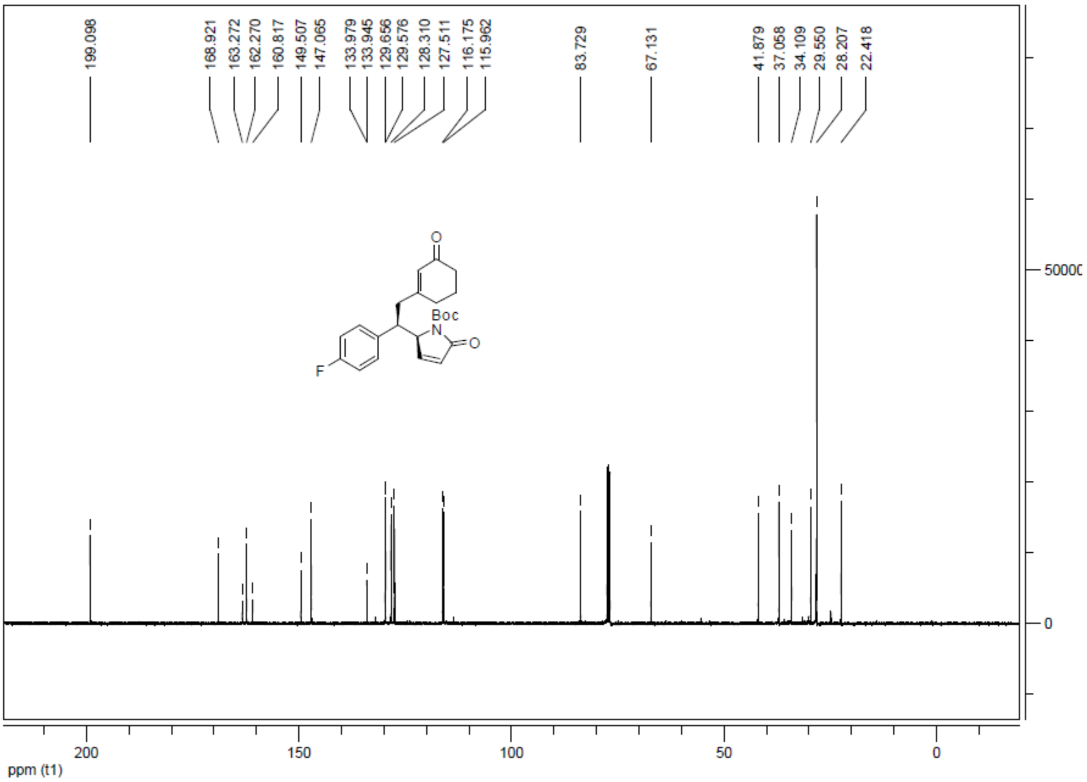

***tert*-Butyl (*S*)-2-((*R*)-1-(4-chlorophenyl)-2-(3-oxocyclohex-1-enyl)ethyl)-5-oxo- 2,5-dihydro-1*H*-pyrrole-1-carboxylate (4j)**

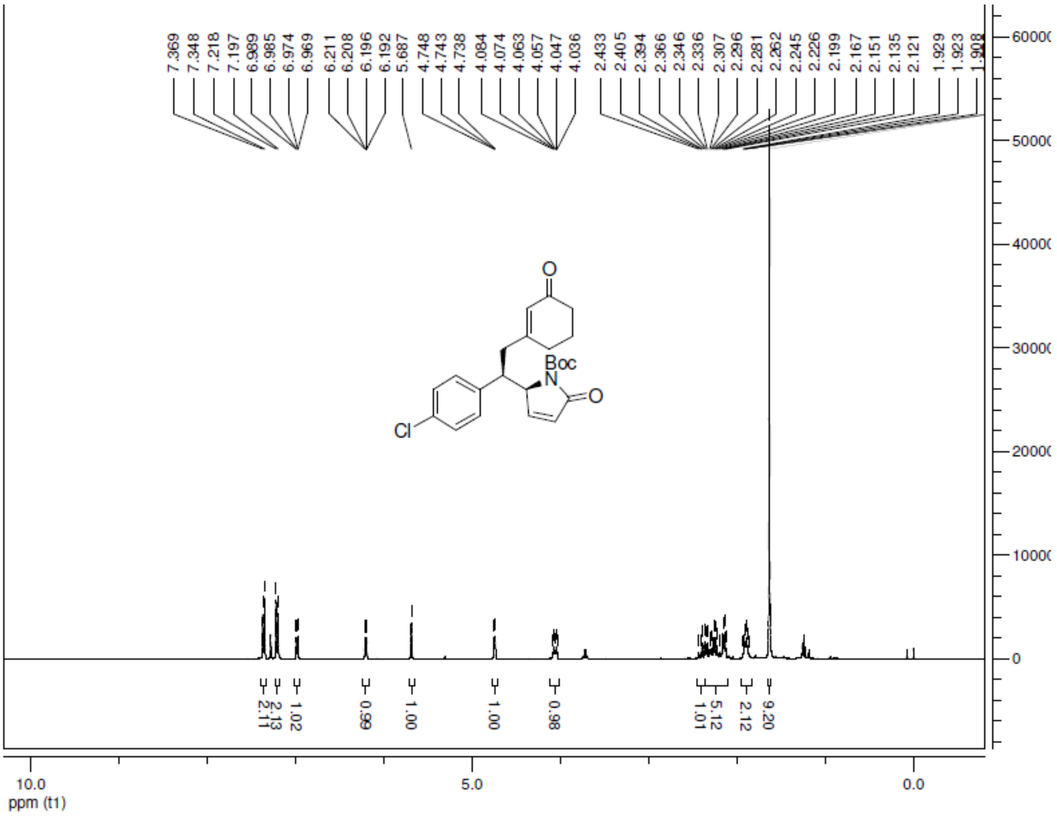

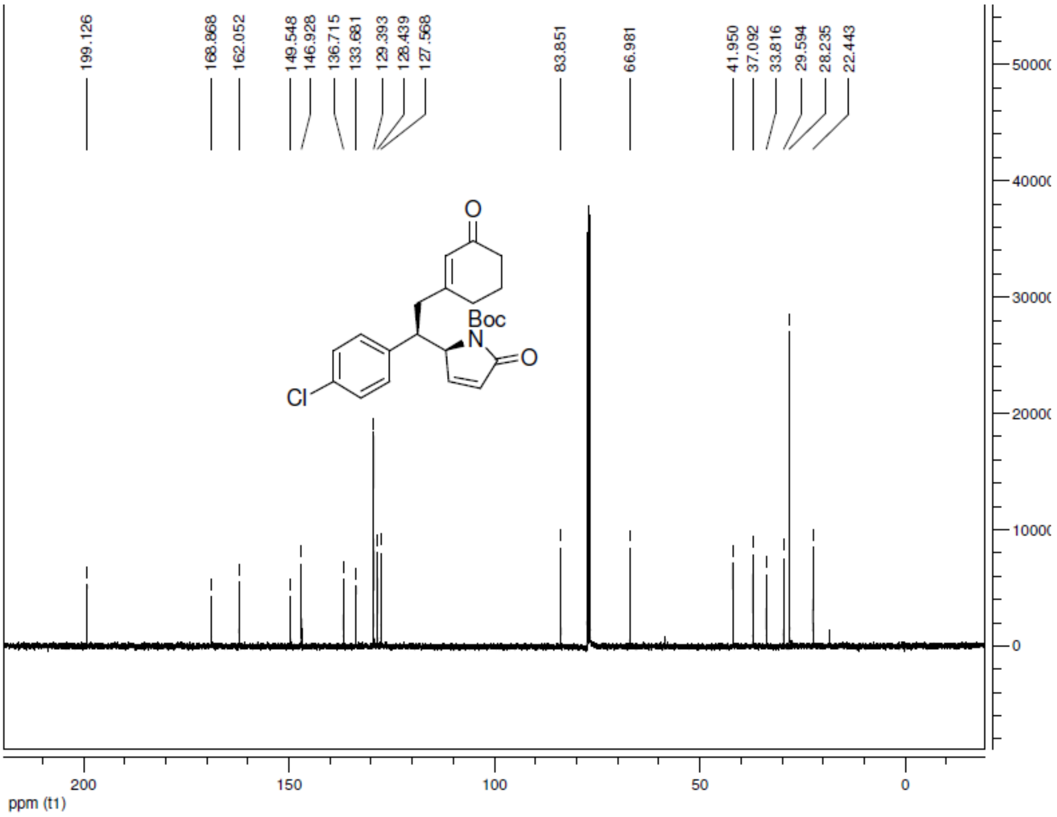

***tert*-Butyl (*R*)-2-oxo-5-((*S*)-1-(3-oxocyclohex-1-enyl)propan-2-yl)-2,5-dihydro- 1*H*-pyrrole-1-carboxylate (4k)**

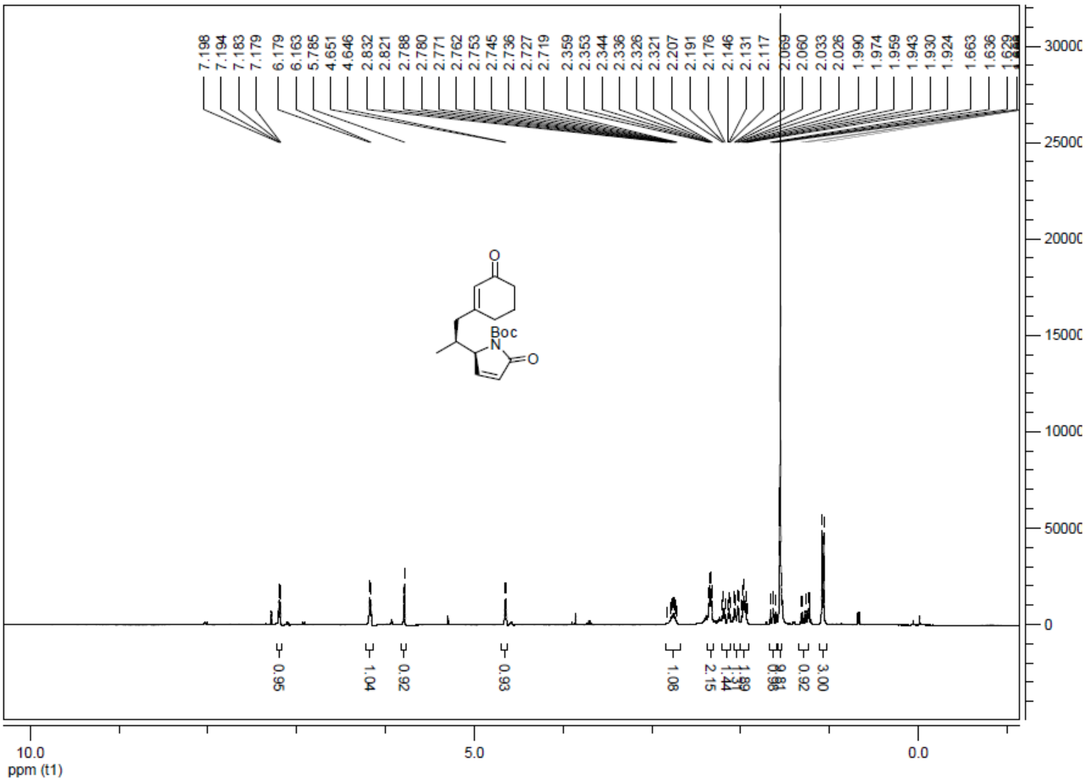

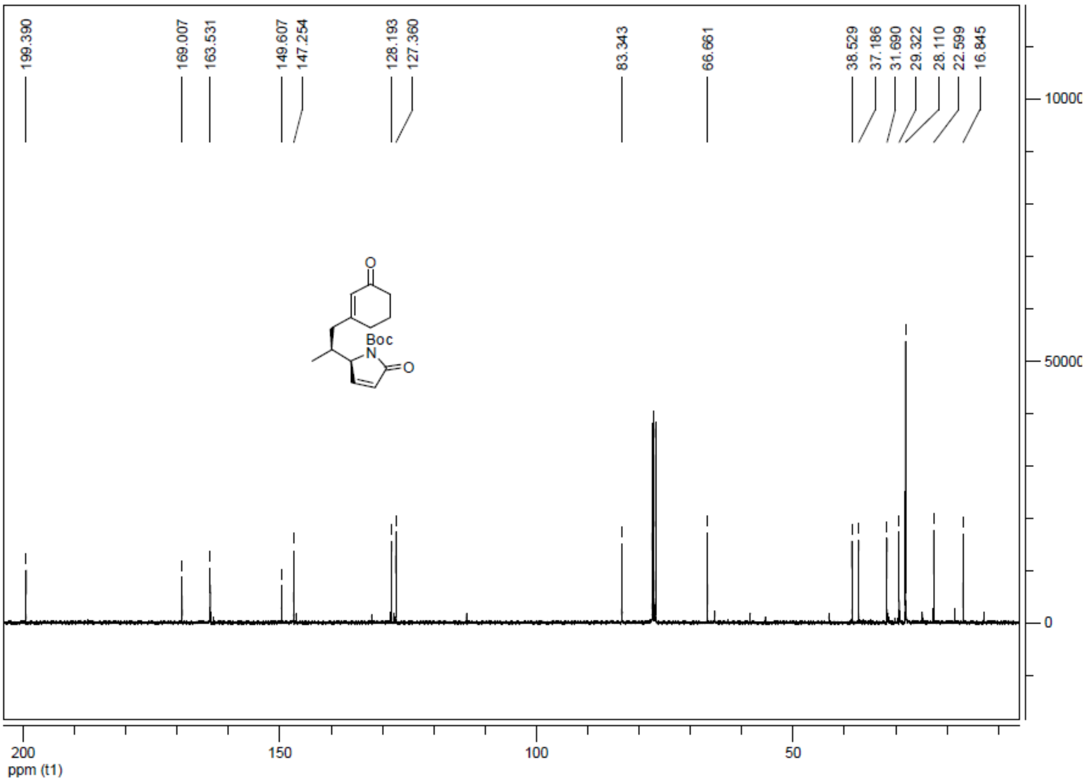

***tert*-Butyl (*R*)-2-oxo-5-((*S*)-1-(3-oxocyclohex-1-enyl)pentan-2-yl)-2,5-dihydro- 1*H*-pyrrole-1-carboxylate (4l)**

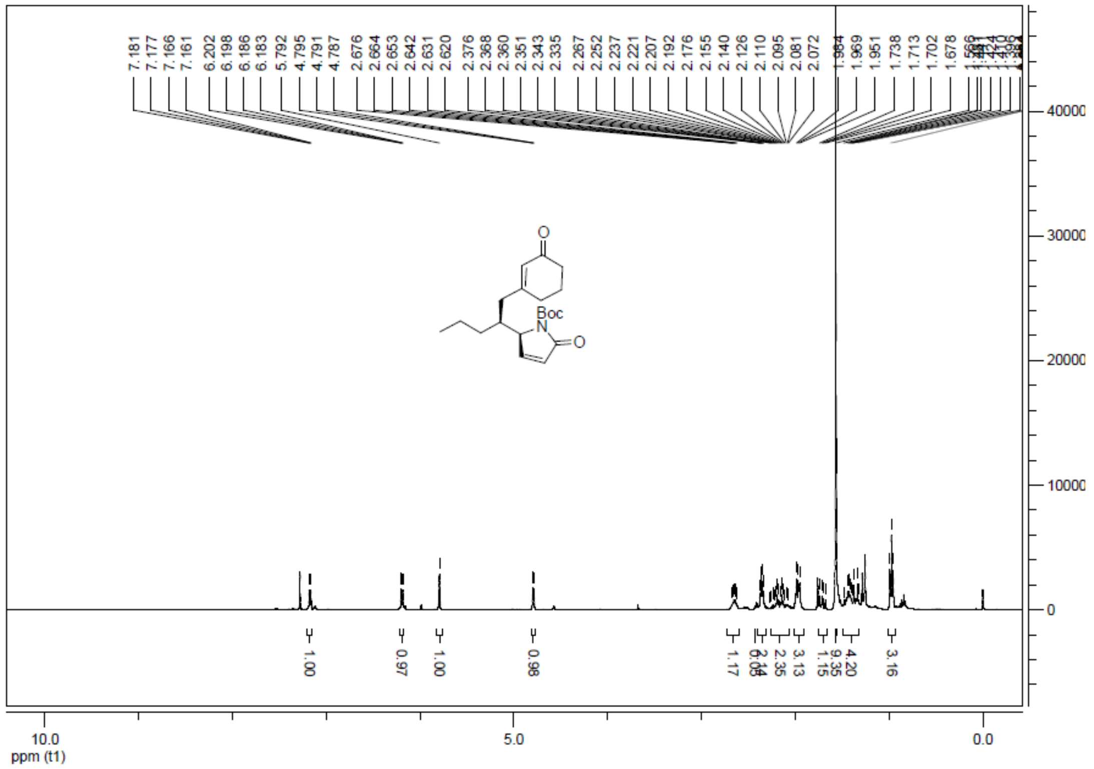

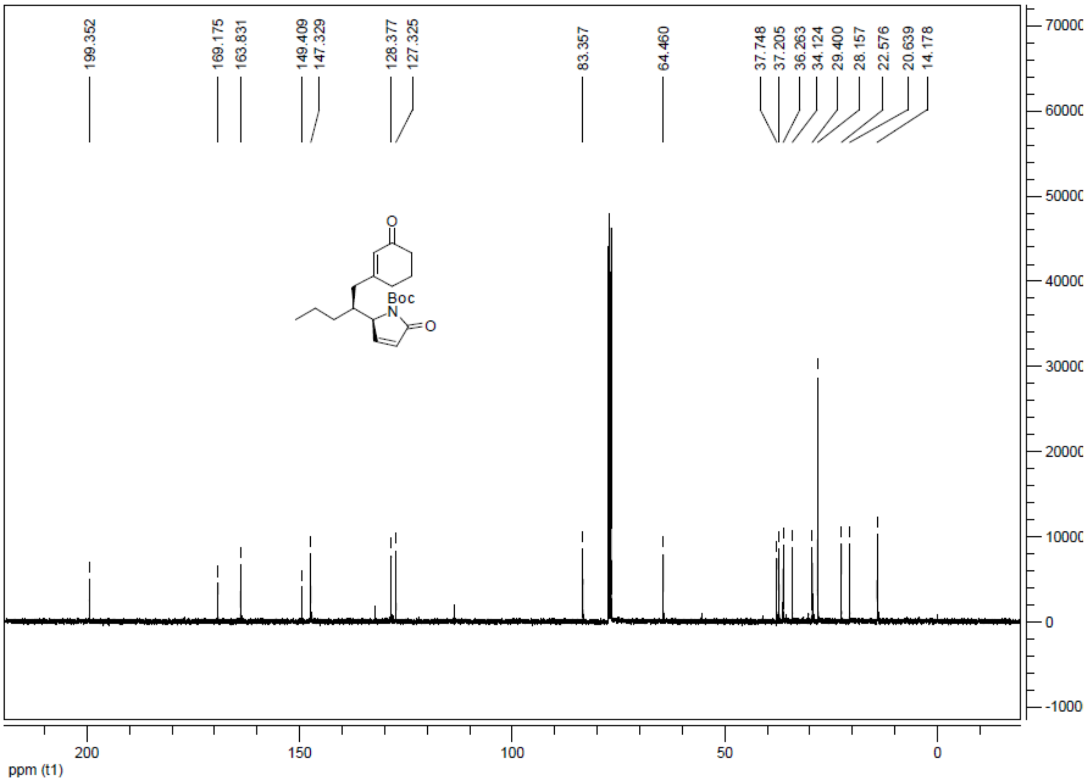

tert-Butyl (_S_)-2-((_R_)-2-(5,5-dimethyl-3-oxocyclohex-1-enyl)-1-phenylethyl)-5-oxo-2,5-dihydro-1_H_-pyrrole-1-carboxylate (4m)

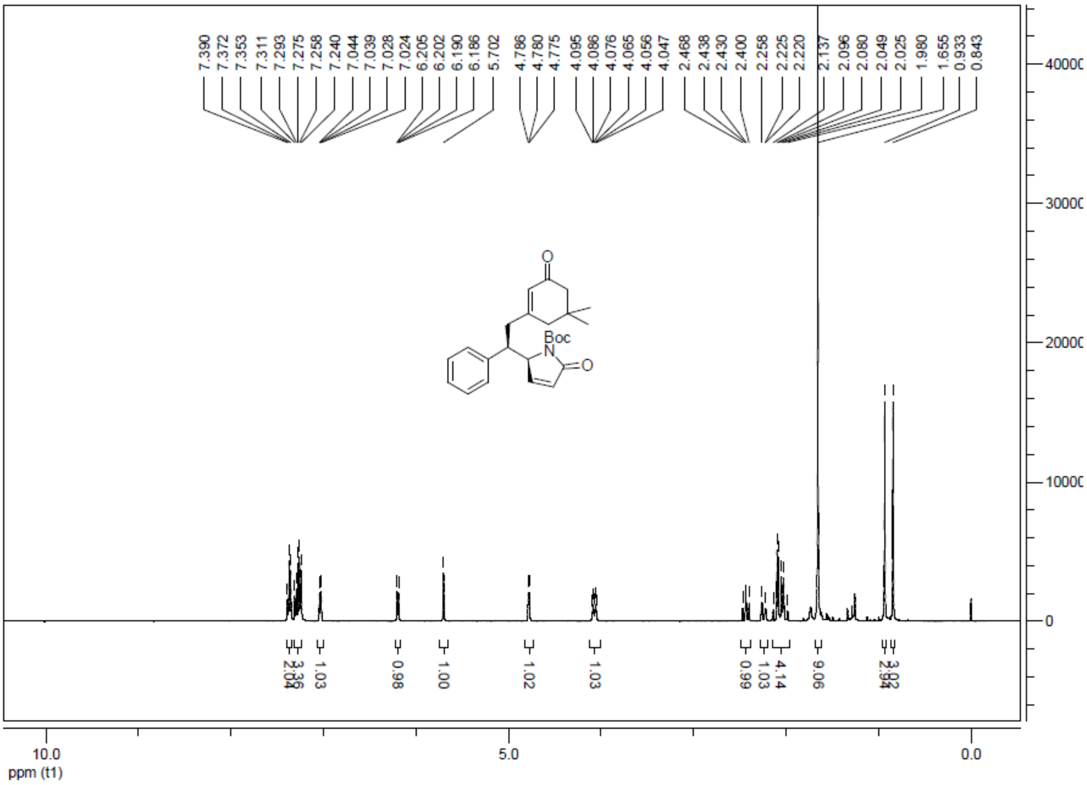

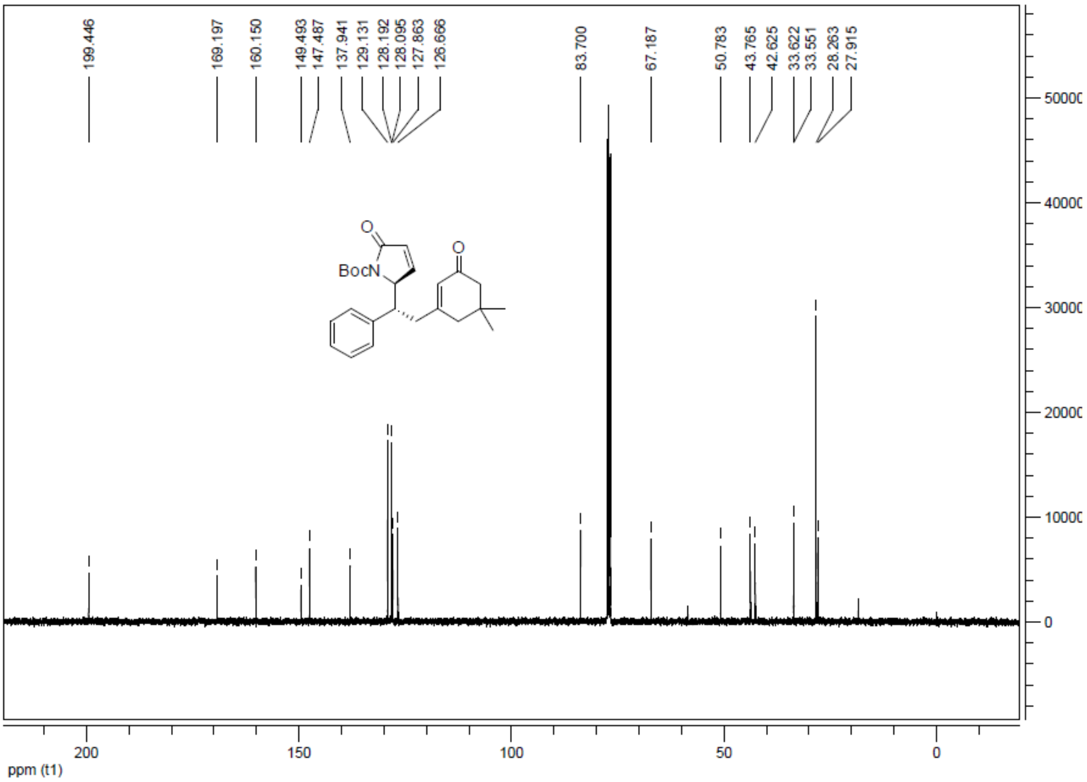

***tert*-Butyl (*S*)-2-((*R*)-2-(6,6-dimethyl-3-oxocyclohex-1-enyl)-1-phenylethyl)-5-oxo-2,5-dihydro-1*H*-pyrrole-1-carboxylate (4n)**

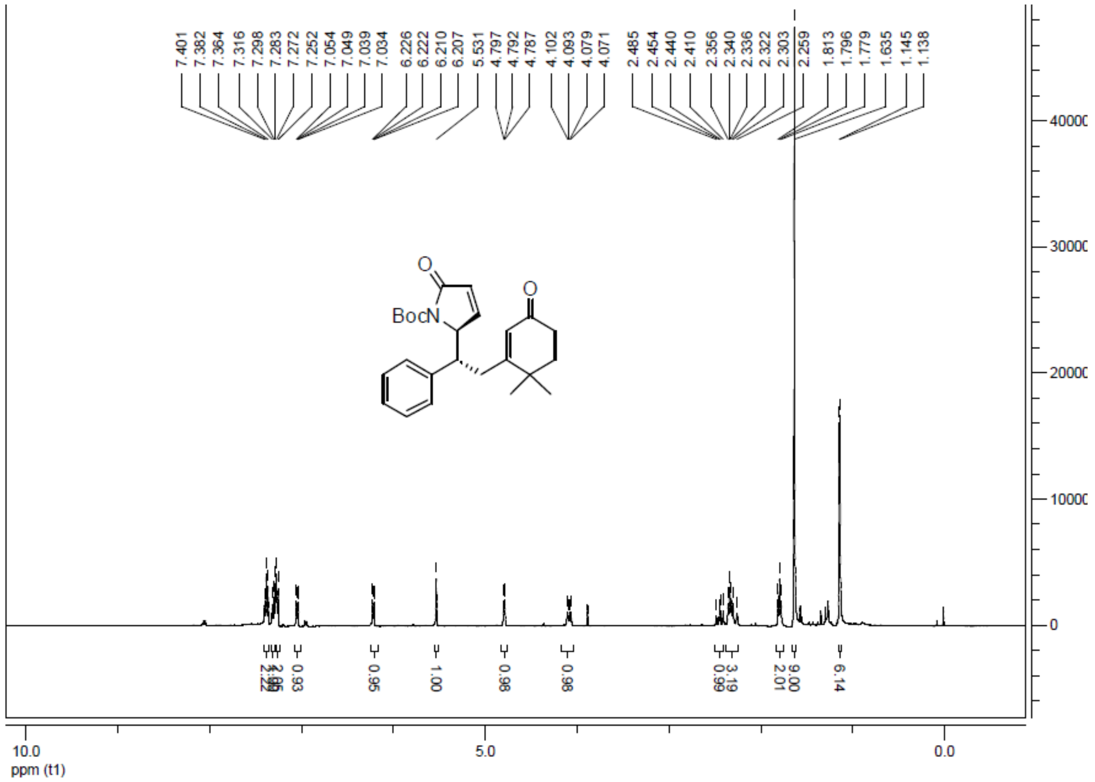

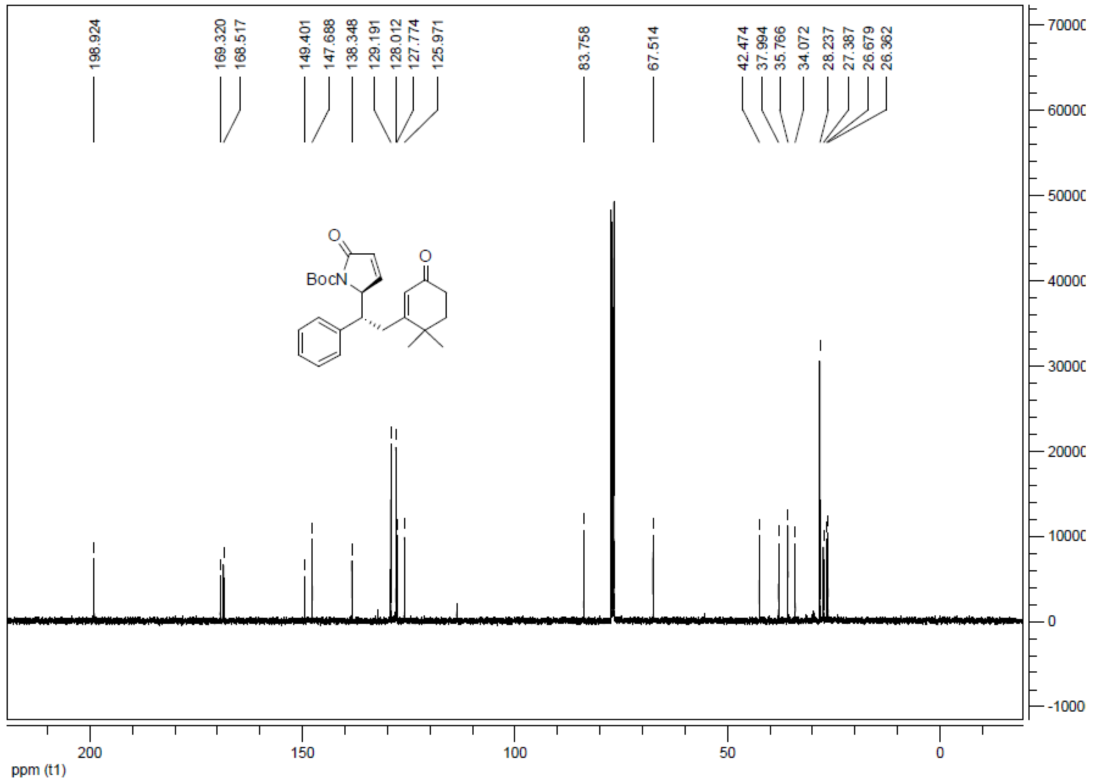

***tert*-Butyl (*S*)-2-((*R*)-1-(naphthalen-2-yl)-2-(3-oxocyclohex-1-enyl)ethyl)-5-oxo-2,5-dihydro-1*H*-pyrrole-1-carboxylate (4o)**

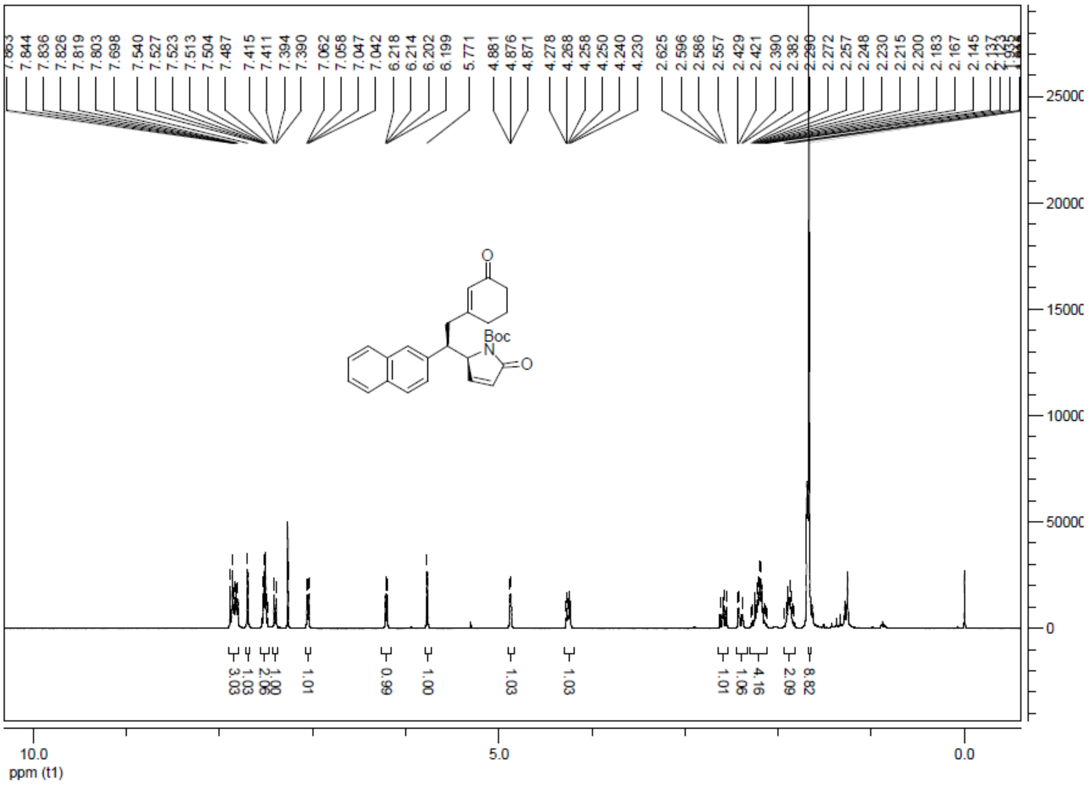

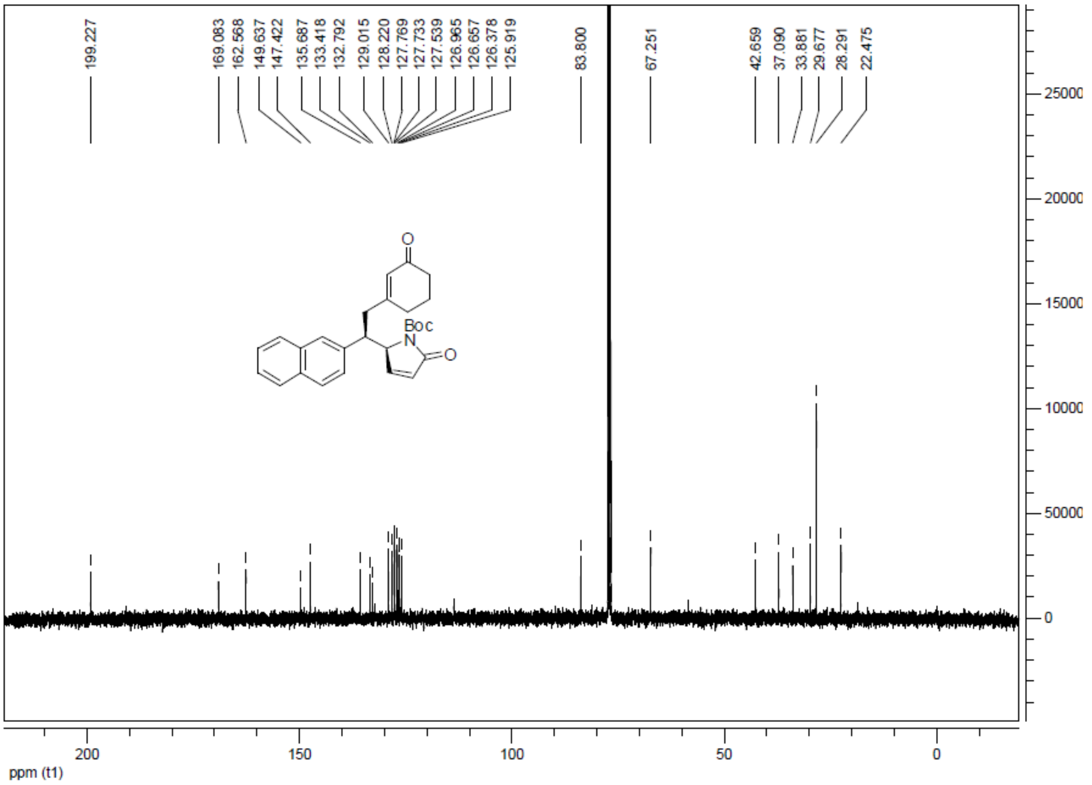

***tert*-Butyl (*S*)-2-oxo-5-((*R*)-2-(3-oxocyclohex-1-enyl)-1-(thiophen-3-yl)ethyl)-2,5-dihydro-1*H*-pyrrole-1-carboxylate (4p)**

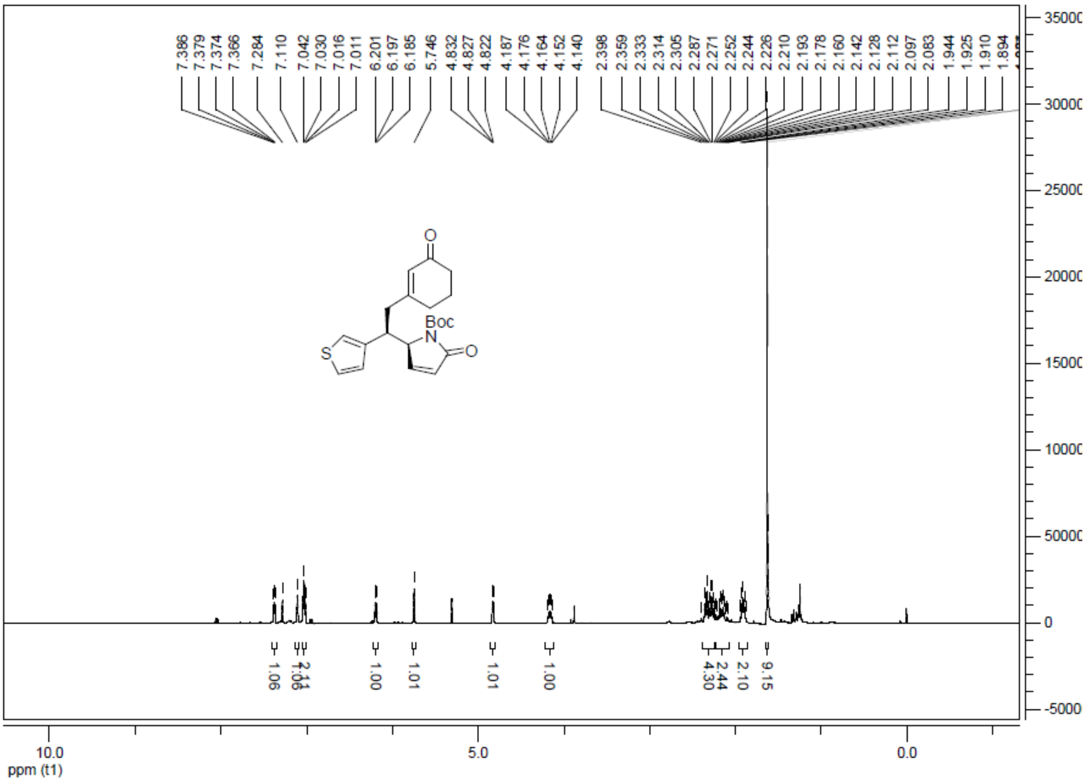

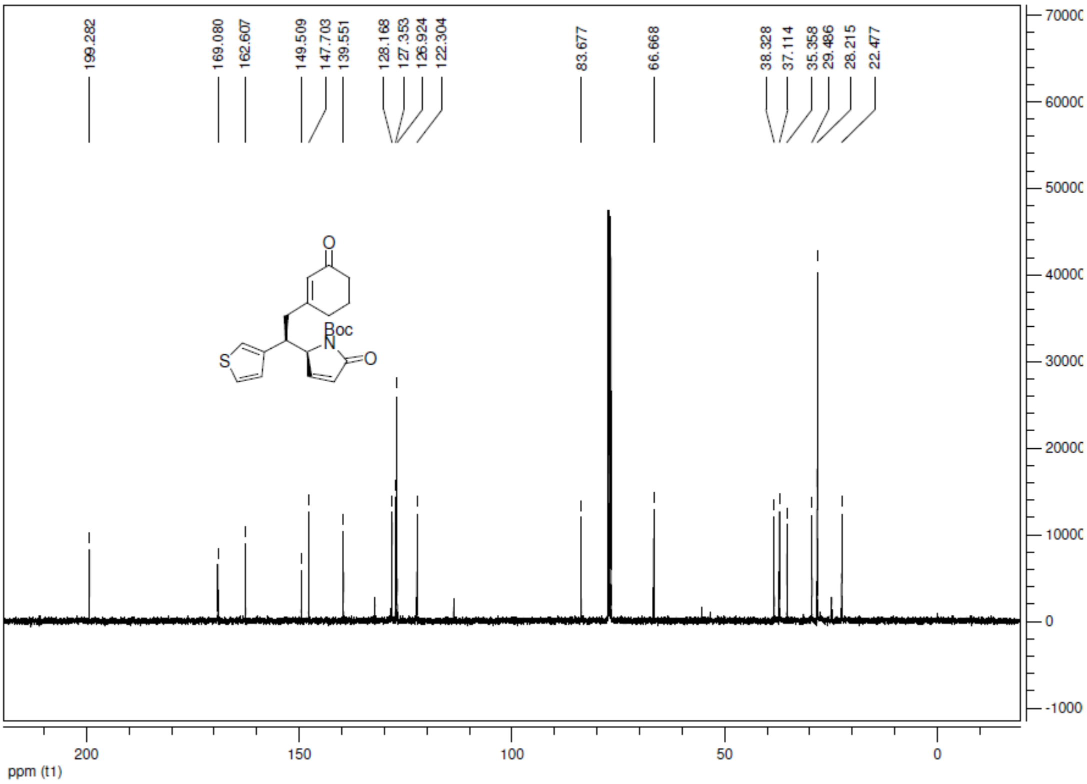

Benzyl (*S*)-2-oxo-5-((*R*)-2-(3-oxocyclohex-1-enyl)-1-phenylethyl)-2,5-dihydro- 1*H*-pyrrole-1-carboxylate (4q)

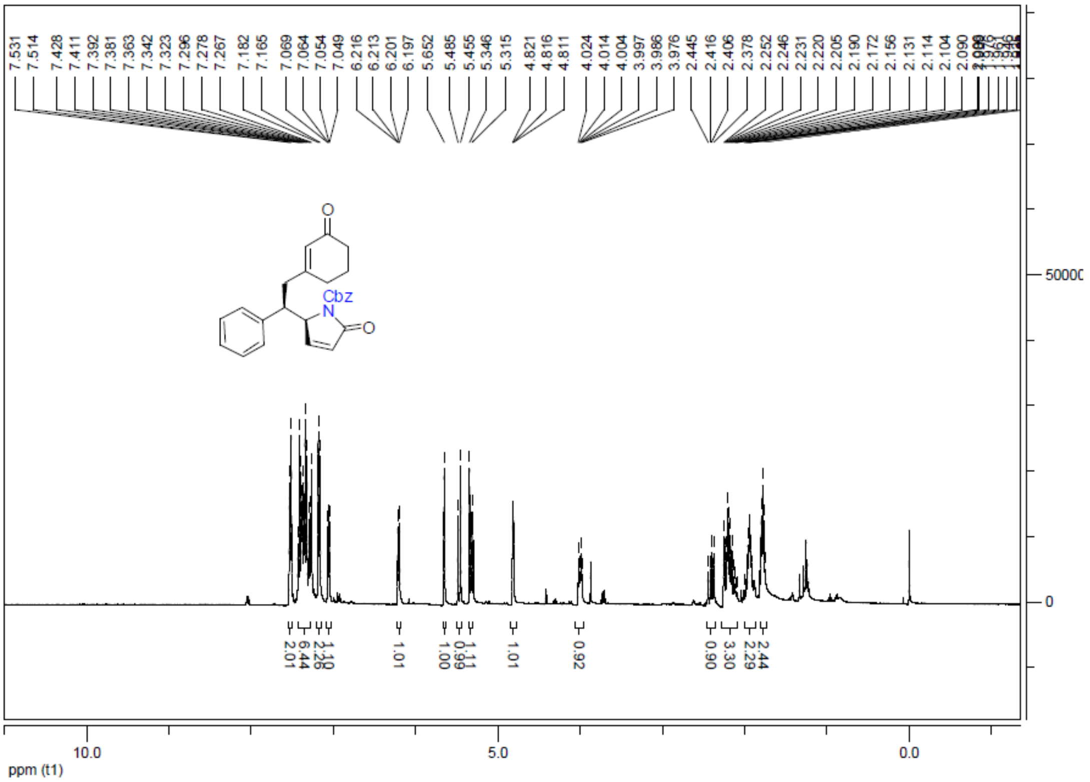

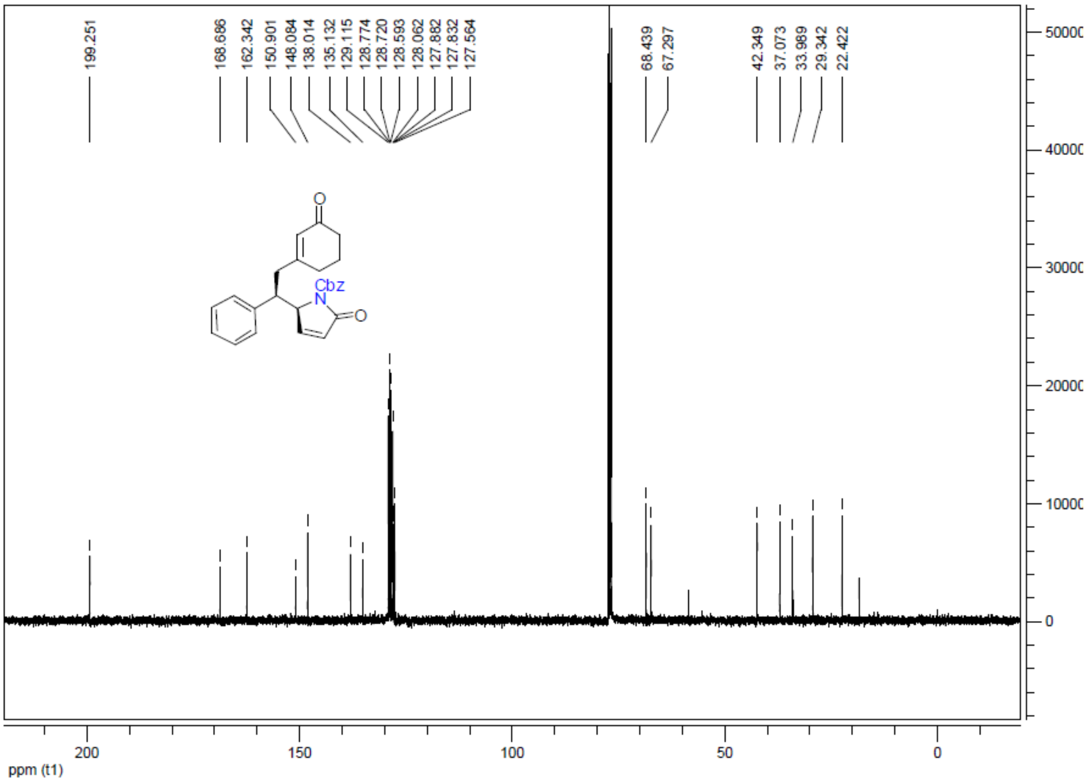

Benzyl (*S*)-2-((*R*)-2-(5,5-dimethyl-3-oxocyclohex-1-enyl)-1-phenylethyl)-5-oxo-2,5-dihydro-1*H*-pyrrole-1-carboxylate (4r)

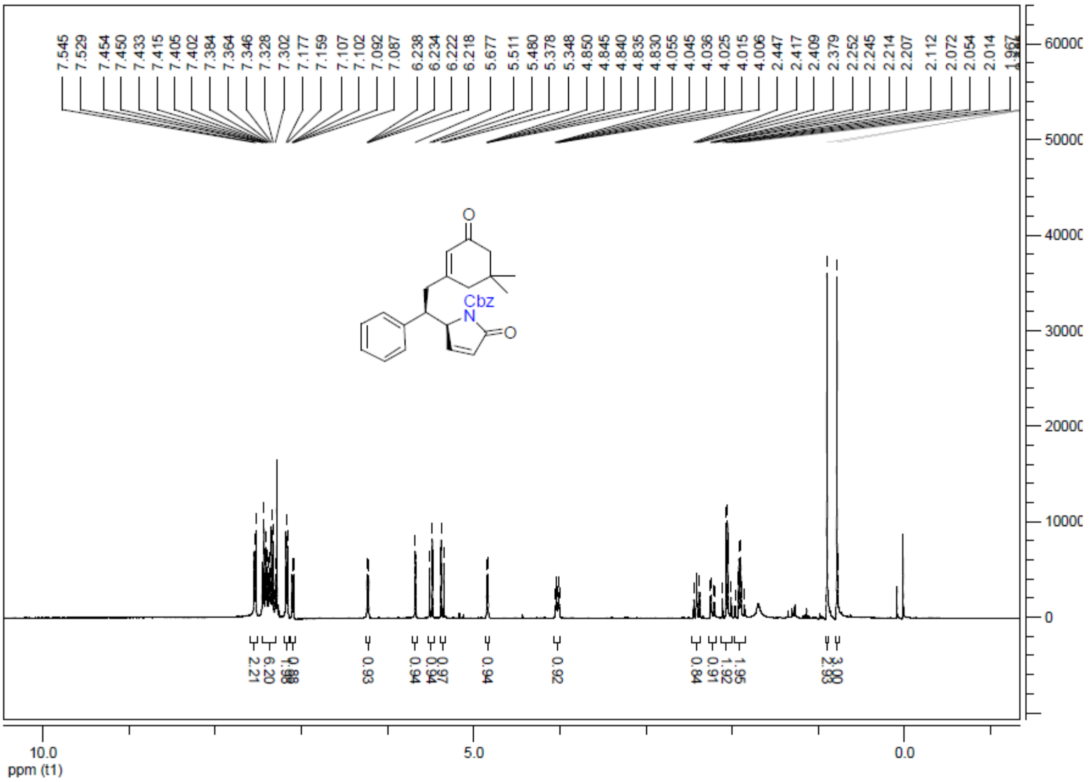

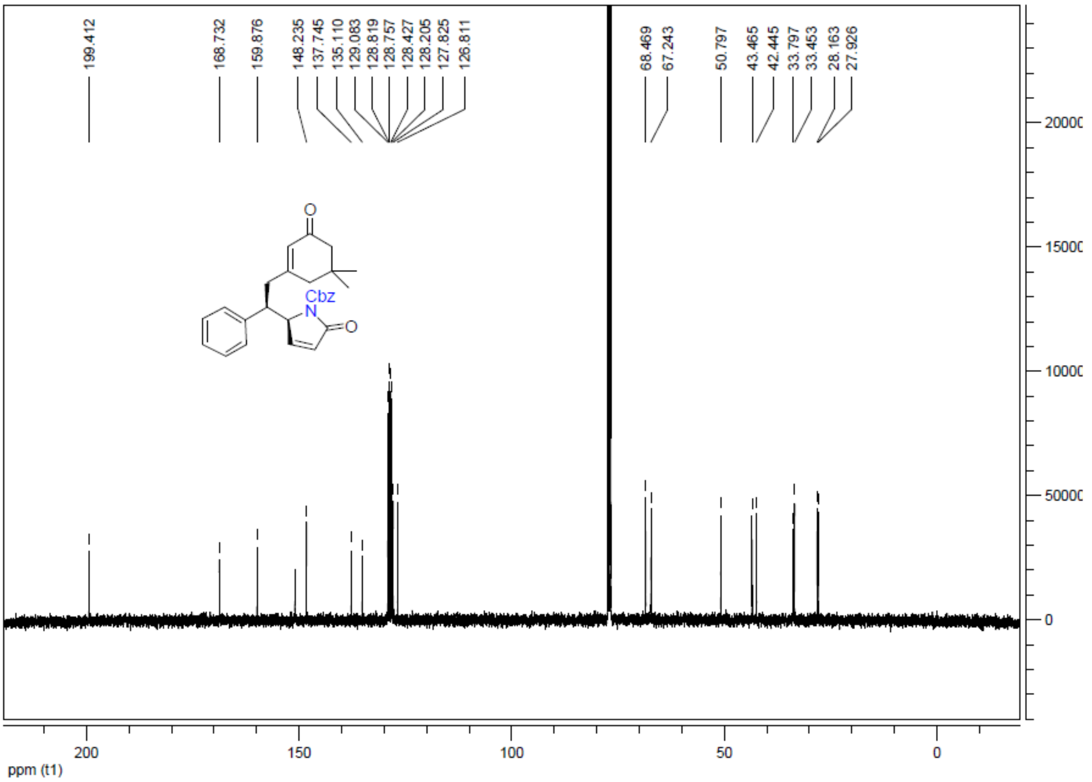

Benzyl (*S*)-2-((*R*)-1-(4-fluorophenyl)-2-(3-oxocyclohex-1-enyl)ethyl)-5-oxo-2,5-dihydro-1*H*-pyrrole-1-carboxylate (4s)

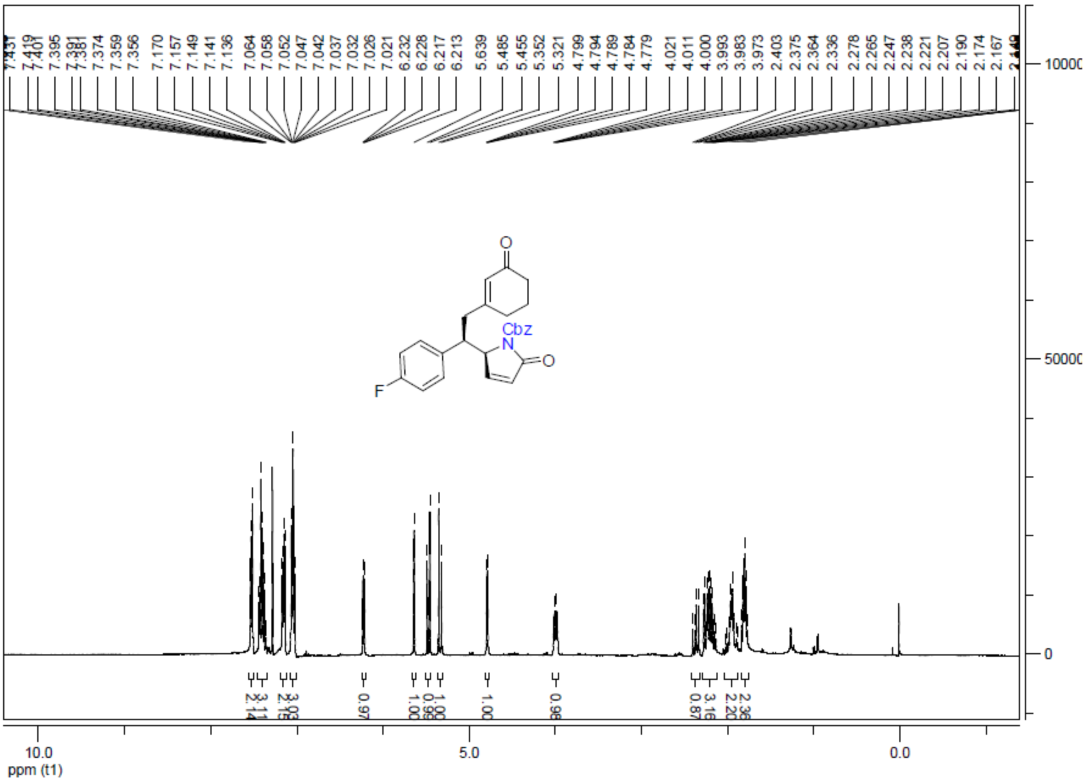

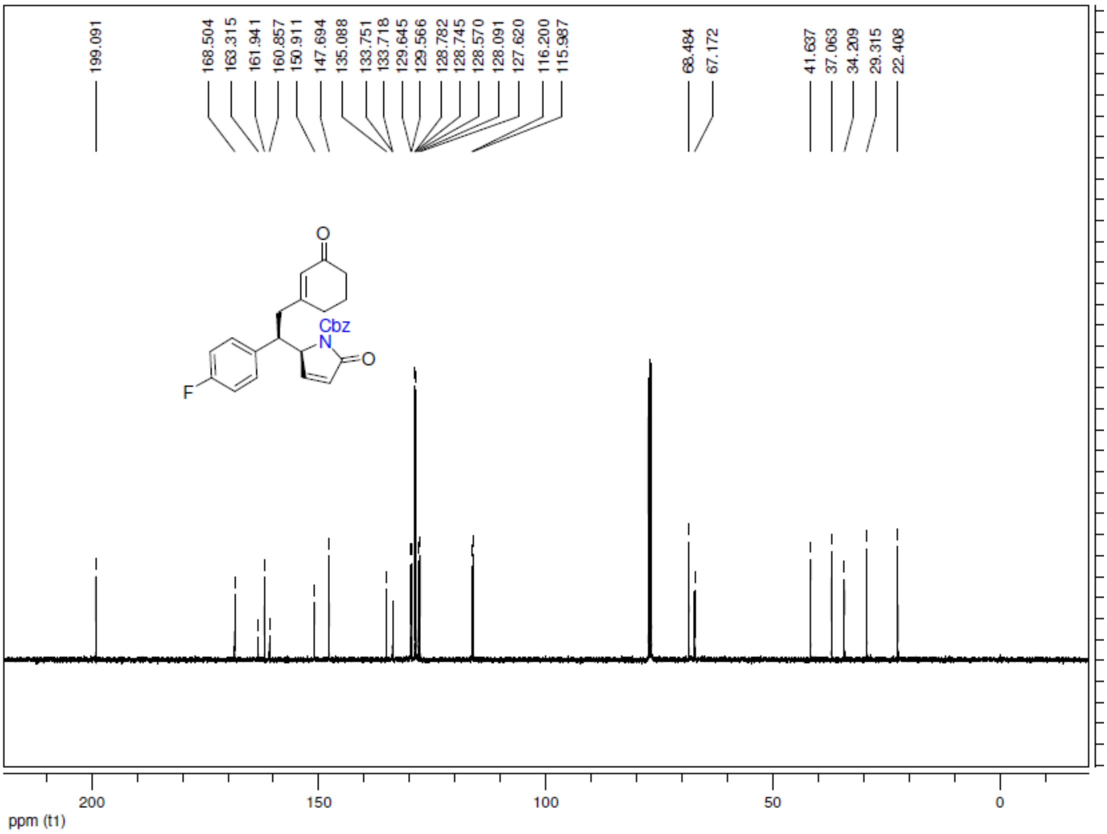

(*S*)-5-((*R*)-2-(3-oxocyclohex-1-enyl)-1-phenylethyl)-1-tosyl-1,5-dihydro-2*H*-pyrrol-2-one (4t)

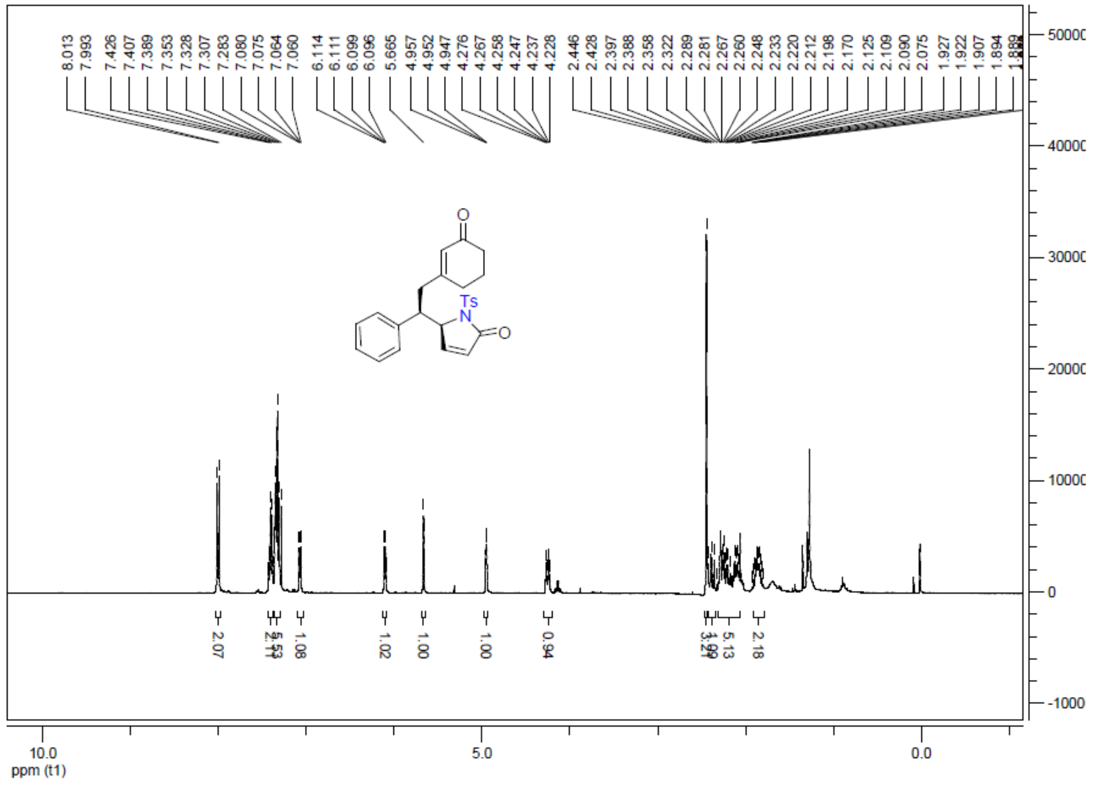

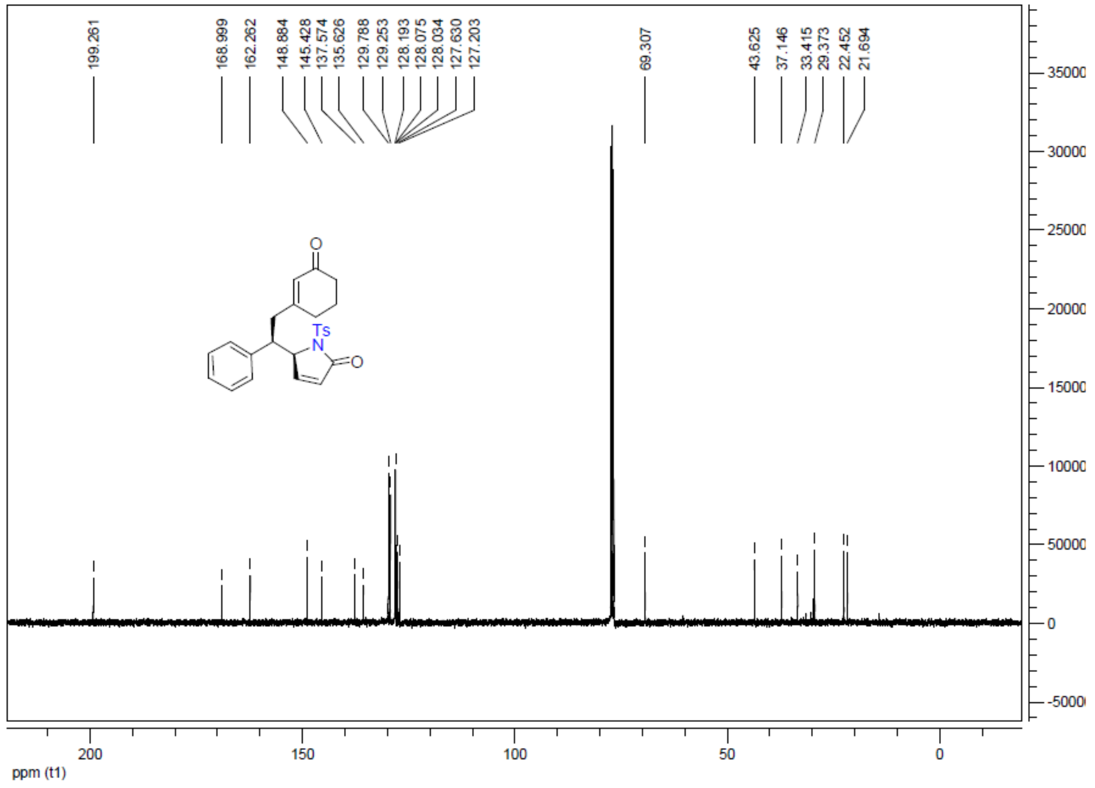

(*S*)-5-((*R*)-1-(4-fluorophenyl)-2-(3-oxocyclohex-1-enyl)ethyl)-1-tosyl-1,5-dihydro-2*H*-pyrrol-2-one (4u)

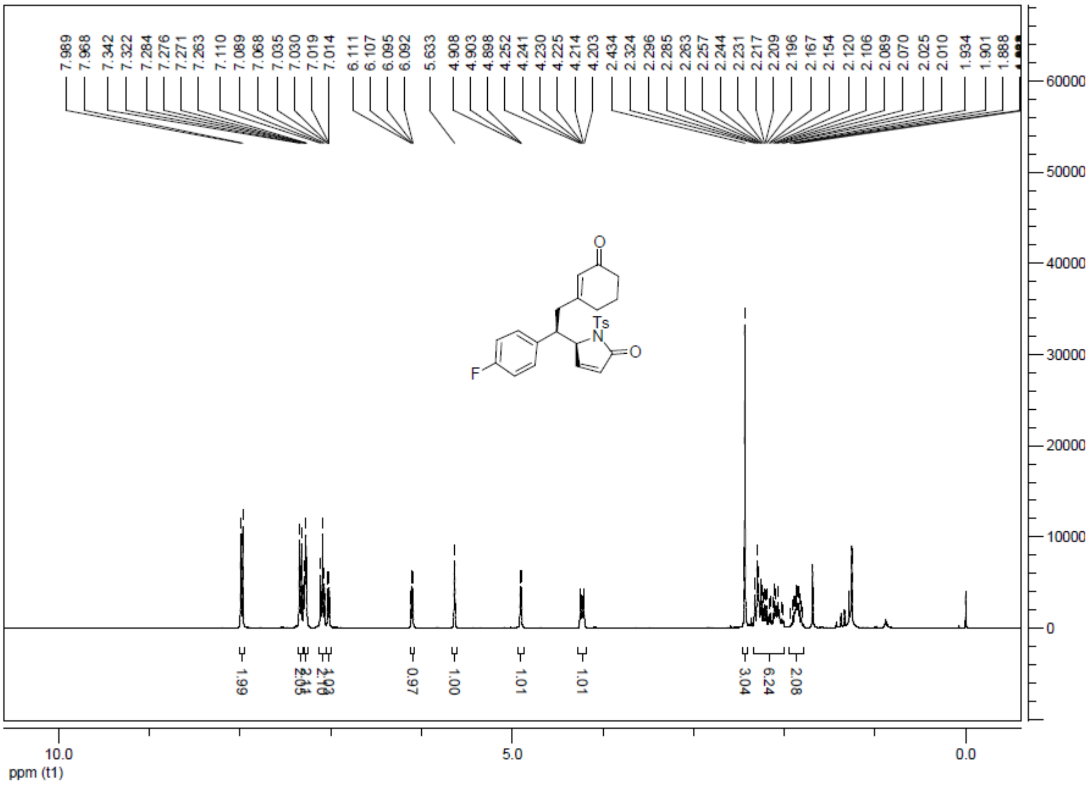

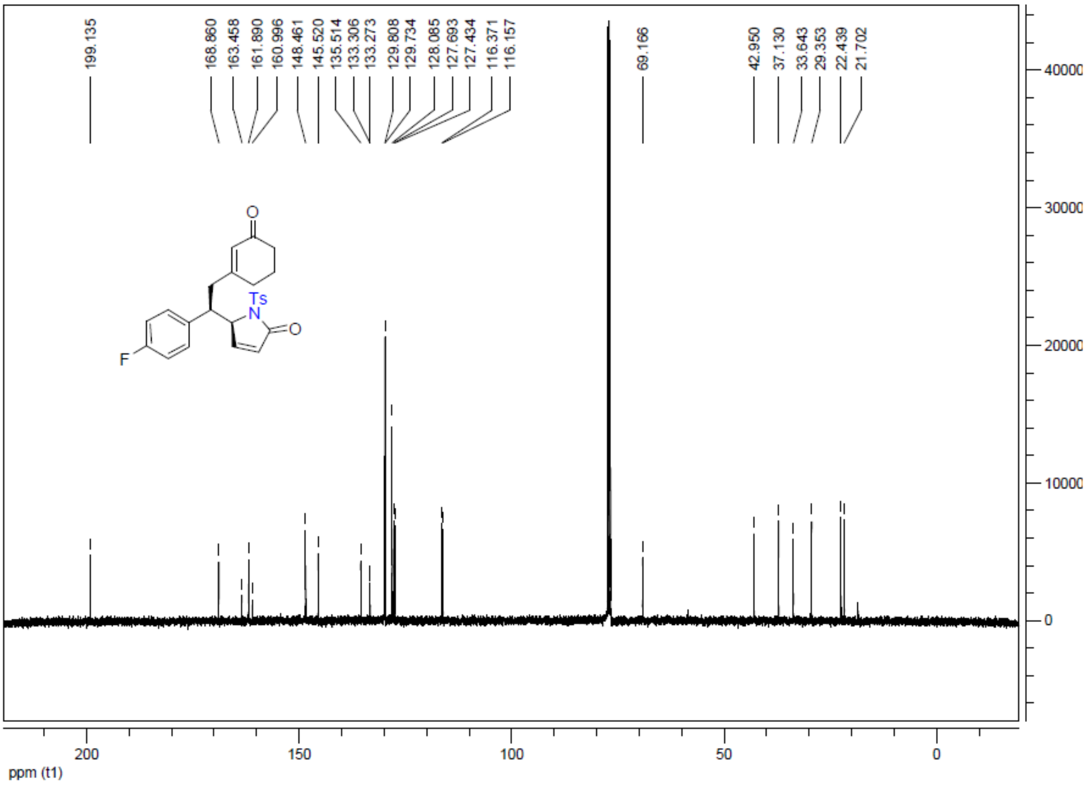

(*S*)-5-((*R*)-1-(3,4-dimethoxyphenyl)-2-(3-oxocyclohex-1-enyl)ethyl)-1-tosyl-1,5-dihydro-2*H*-pyrrol-2-one (4v)

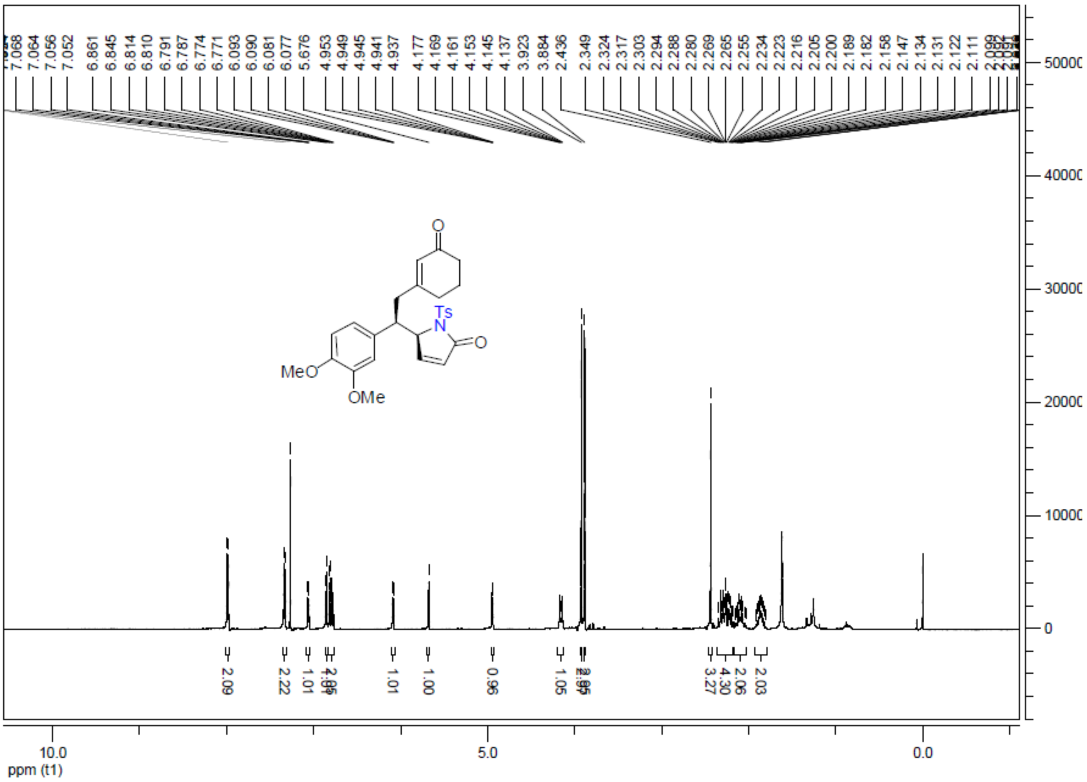

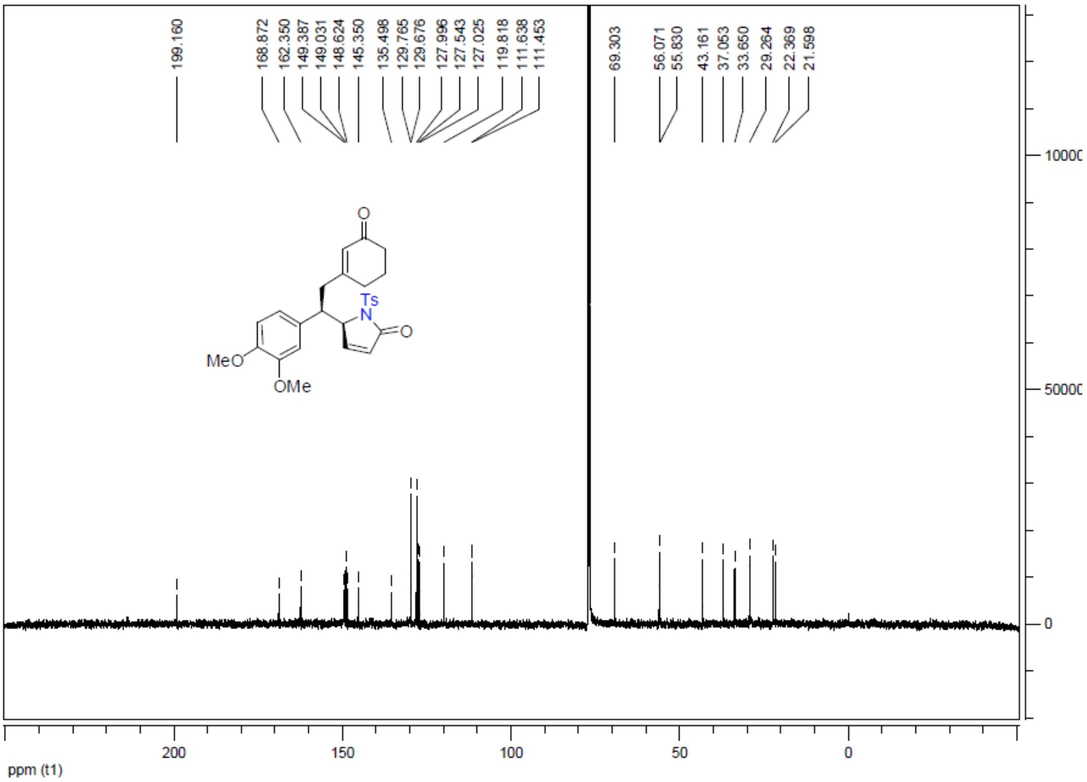

***tert*-Butyl (3a*R*,4*R*,5a*S*,8b*S*)-2,7-dioxo-4-phenyl-1,3a,4,5,5a,6,7,8b-octahydrocyclopenta[*e*]indole-3(2*H*)-carboxylate (6a)**

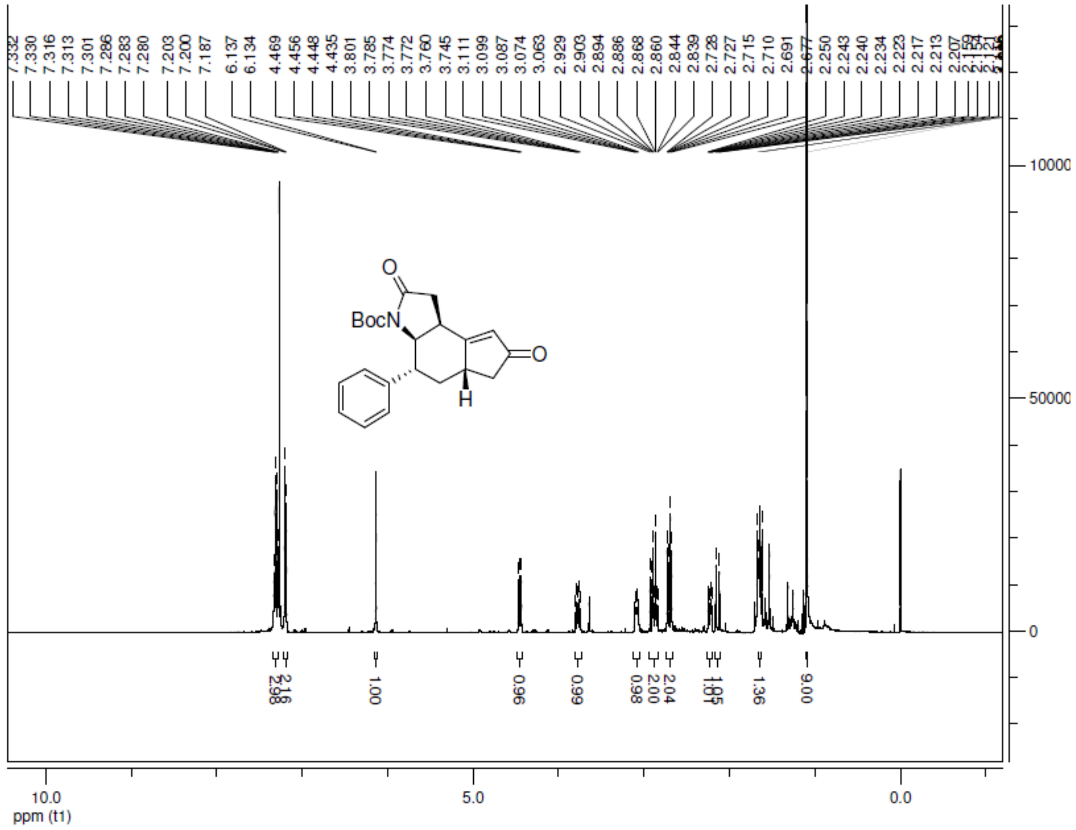

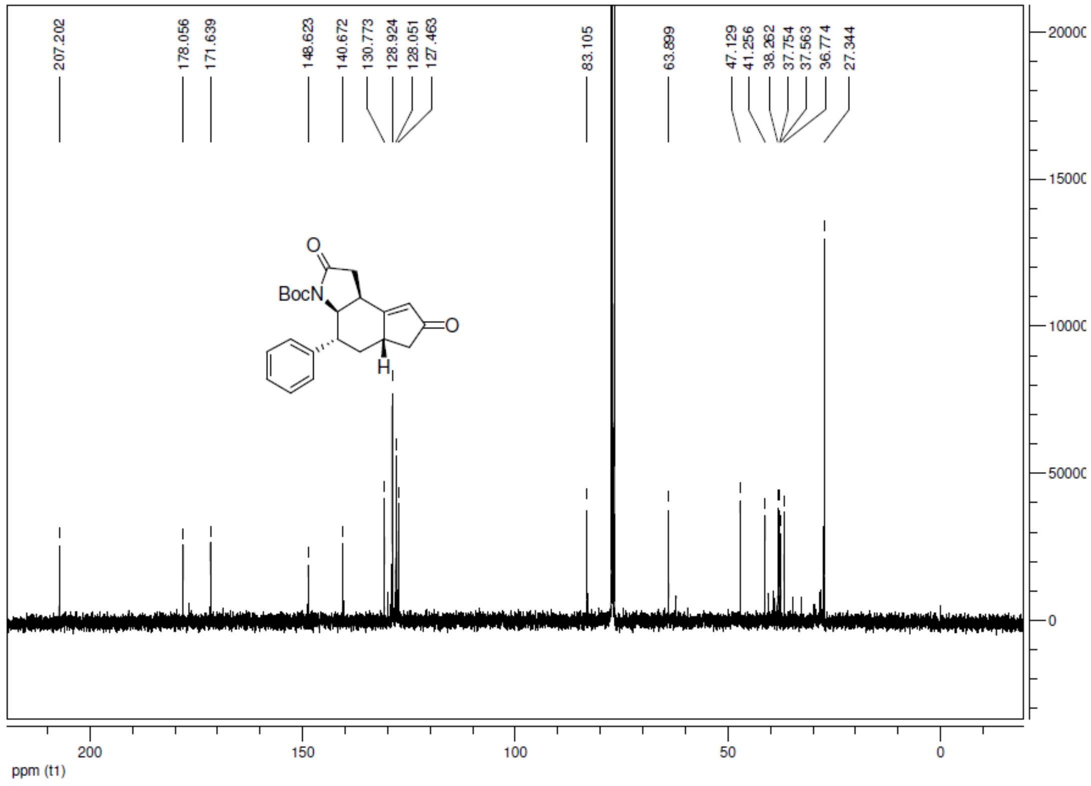

***tert*-Butyl (3a*R*,4*R*,5a*S*,8b*S*)-2,7-dioxo-4-(*p*-tolyl)-1,3a,4,5,5a,6,7,8b-octahydrocyclopenta[*e*]indole-3(2*H*)-carboxylate (6b)**

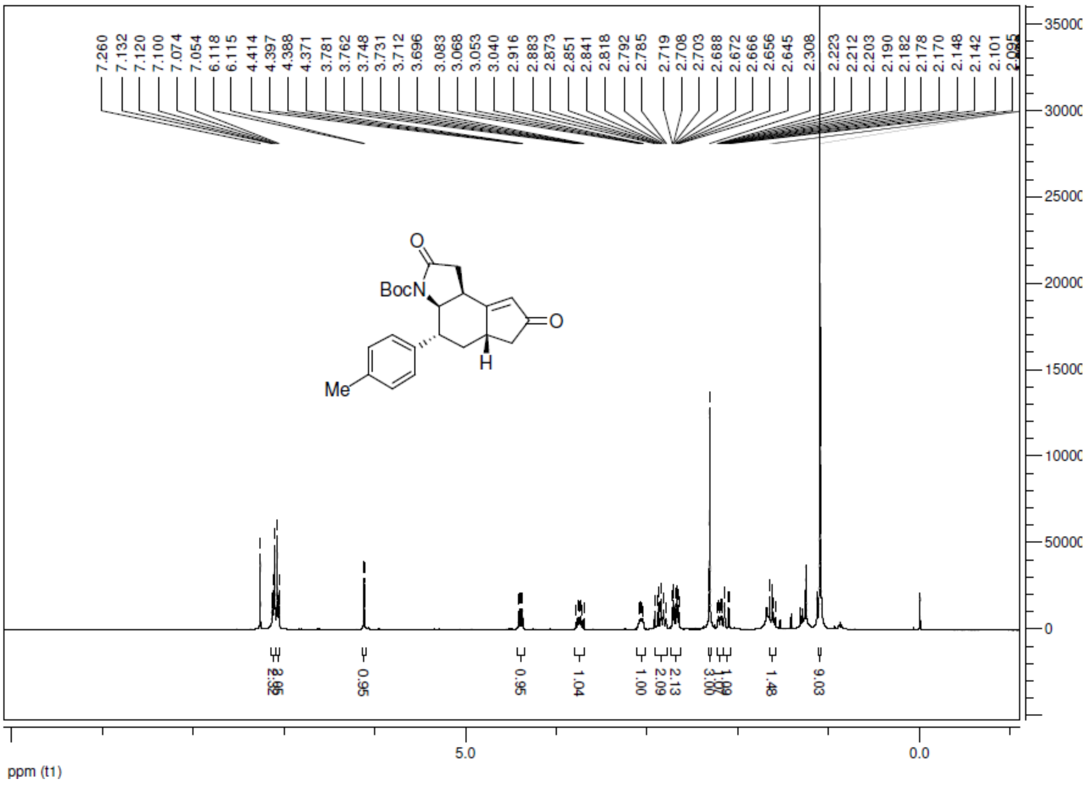

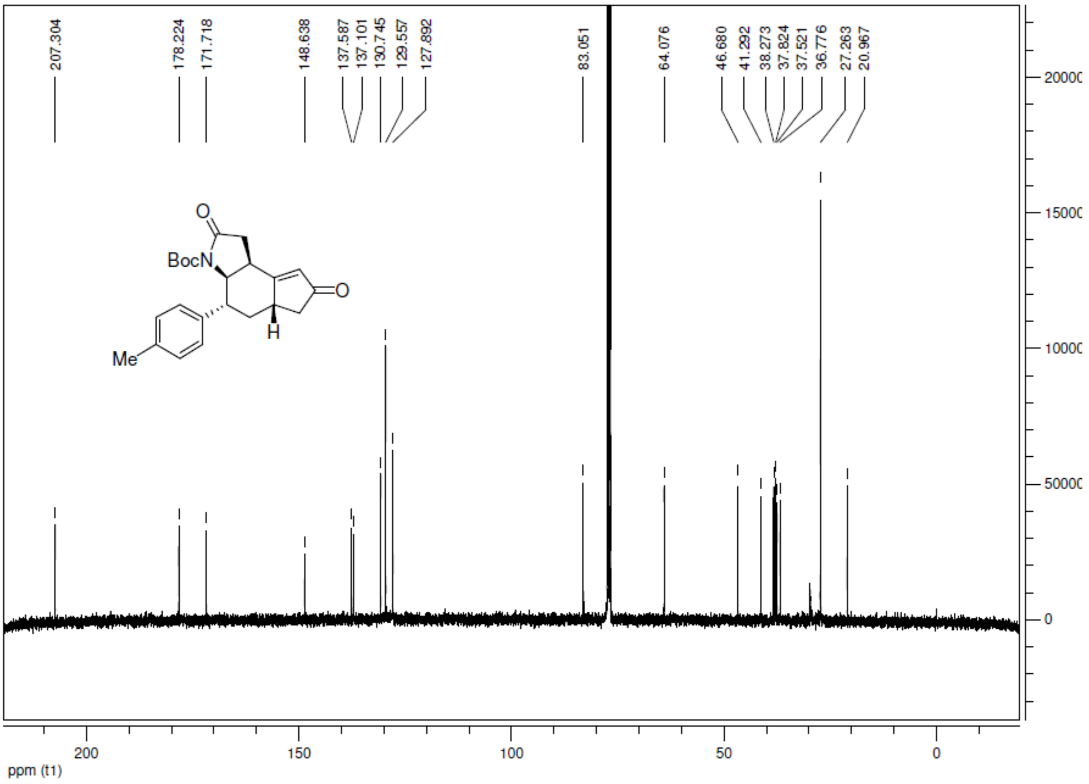

***tert*-Butyl (3a*R*,4*R*,5a*S*,8b*S*)-4-(4-(*tert*-butyl)phenyl)-2,7-dioxo-1,3a,4,5,5a,6,7,8b-octahydrocyclopenta[*e*]indole-3(2*H*)-carboxylate (6c)**

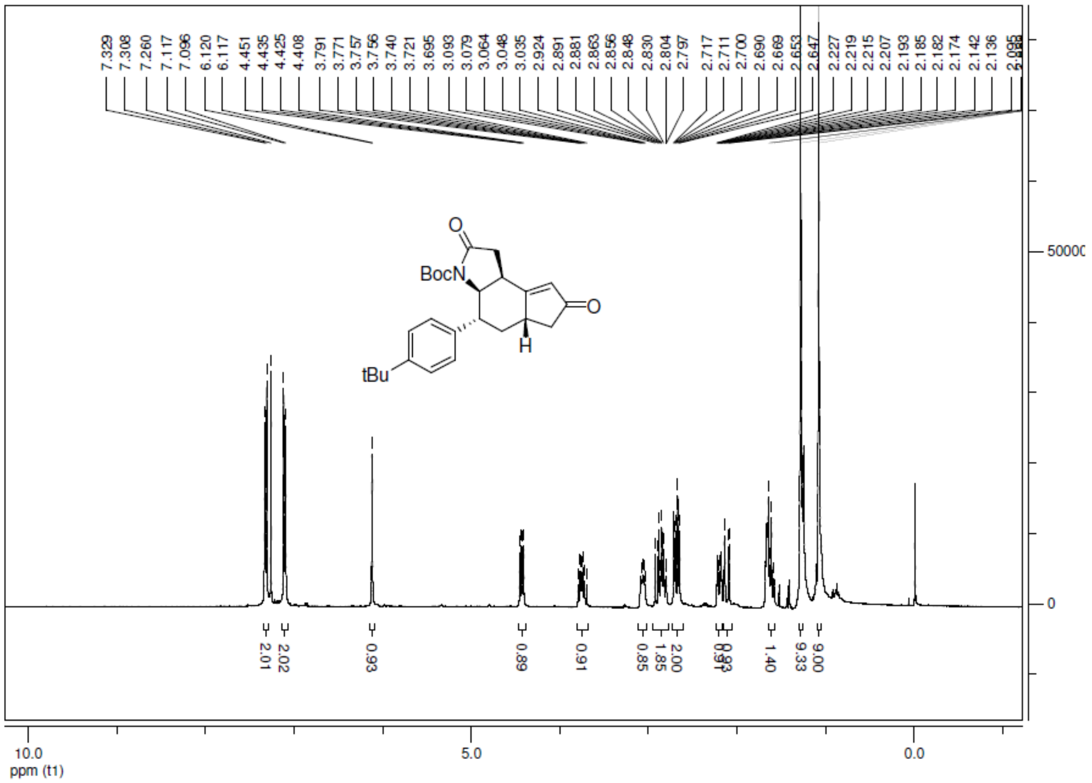

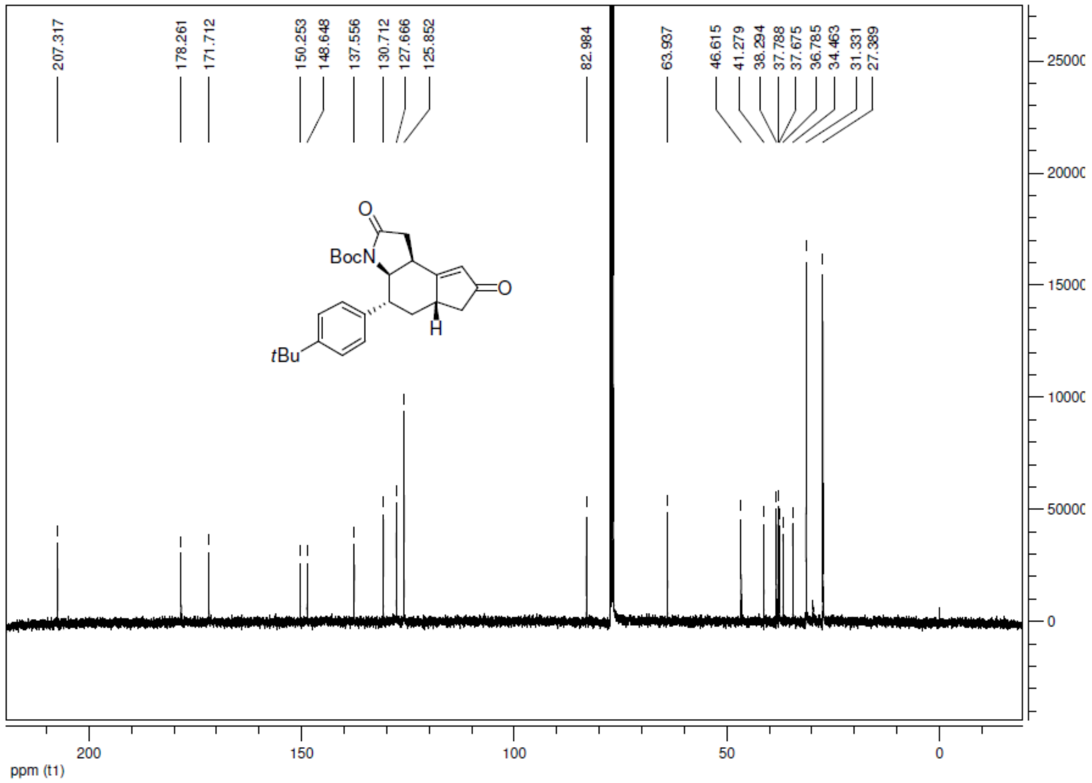

***tert*-Butyl (3a*R*,4*R*,5a*S*,8b*S*)-4-(naphthalen-2-yl)-2,7-dioxo-1,3a,4,5,5a,6,7,8b-octahydrocyclopenta[*e*]indole-3(2*H*)-carboxylate (6d)**

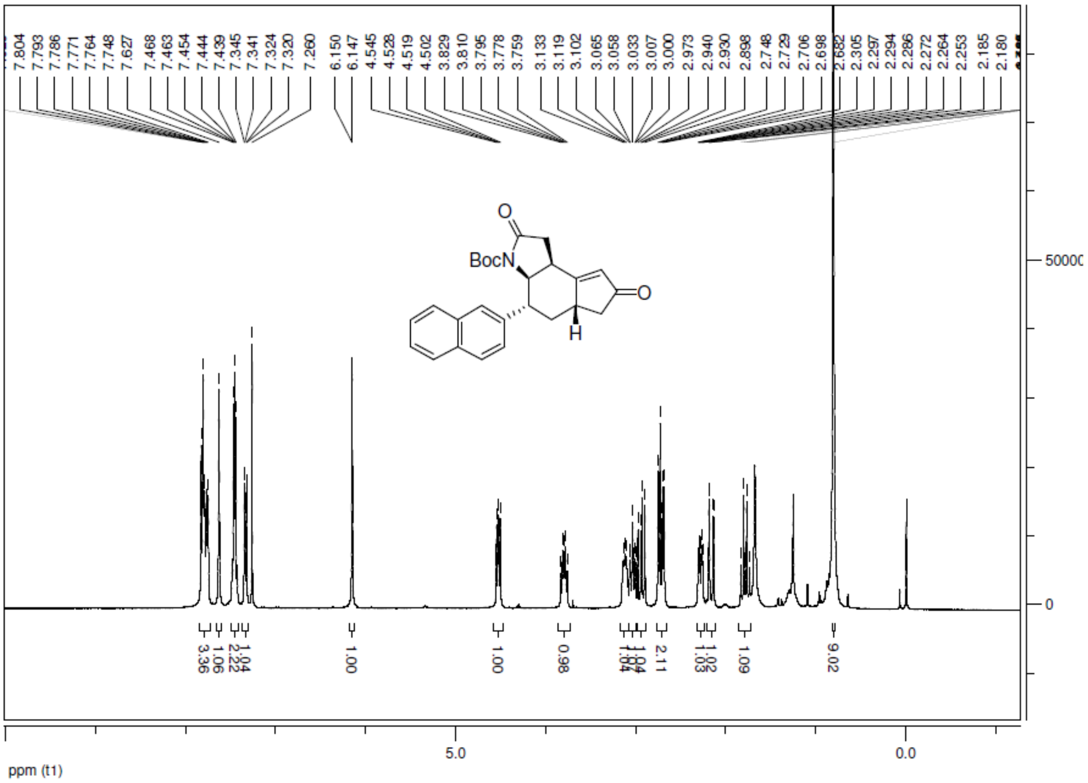

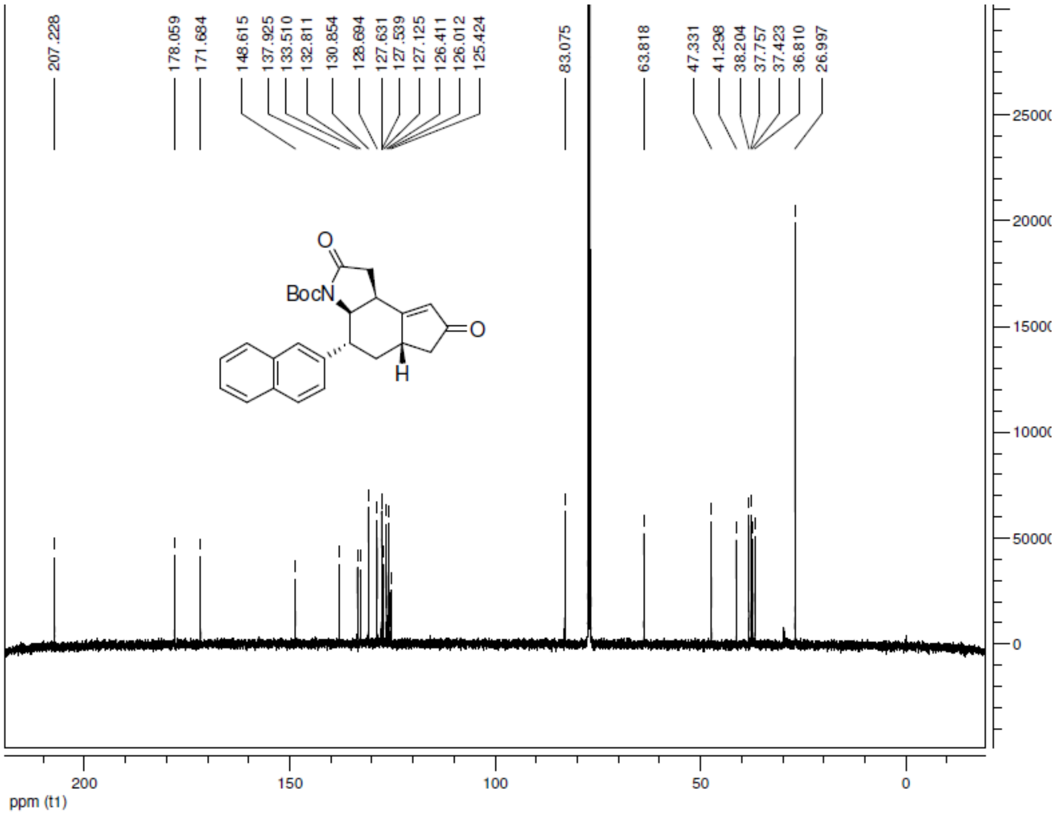

***tert*-Butyl (3a*S*,4*R*,5a*S*,8b*S*)-2,7-dioxo-4-(thiophen-3-yl)-1,3a,4,5,5a,6,7,8b-octahydrocyclopenta[*e*]indole-3(2*H*)-carboxylate (6e)**

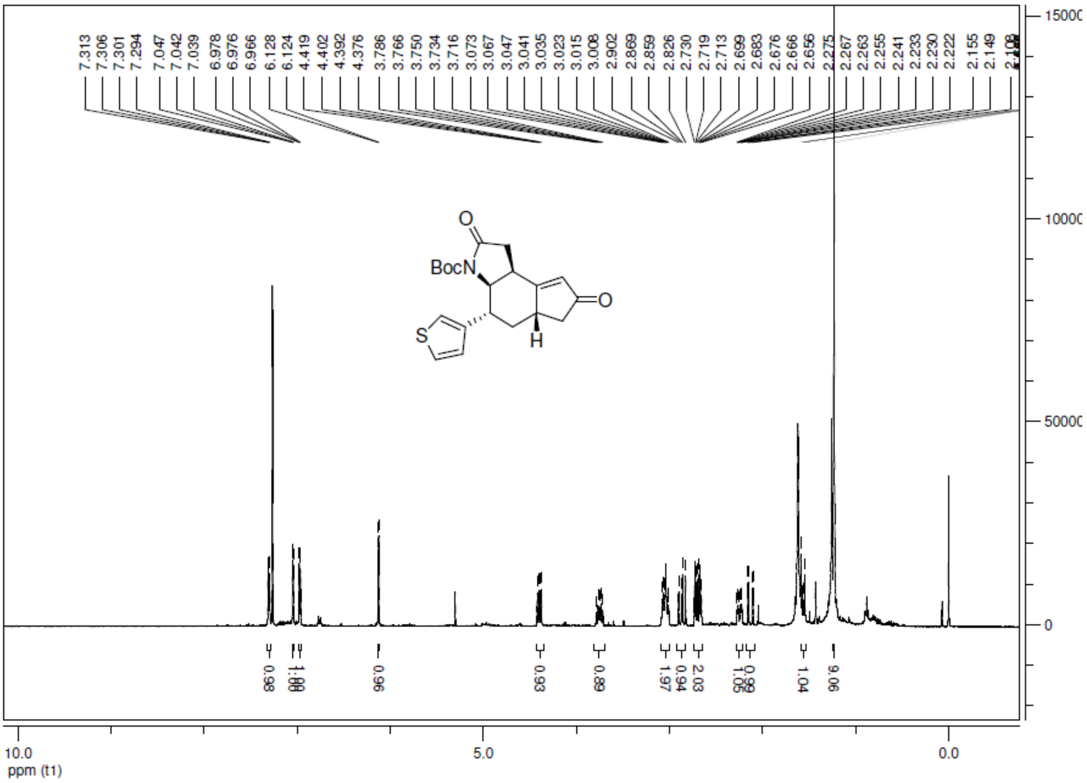

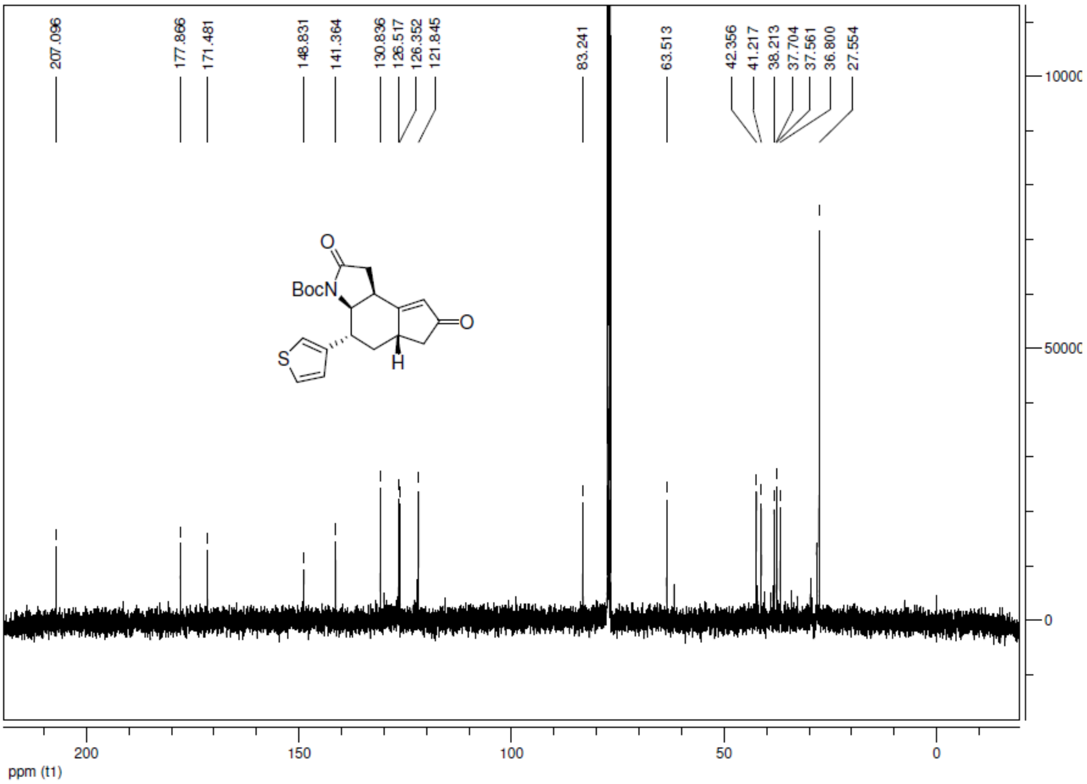

Benzyl (3a*R*,4*R*,5a*S*,8b*S*)-2,7-dioxo-4-phenyl-1,3a,4,5,5a,6,7,8b-octahydrocyclopenta[*e*]indole-3(2*H*)-carboxylate (6f)

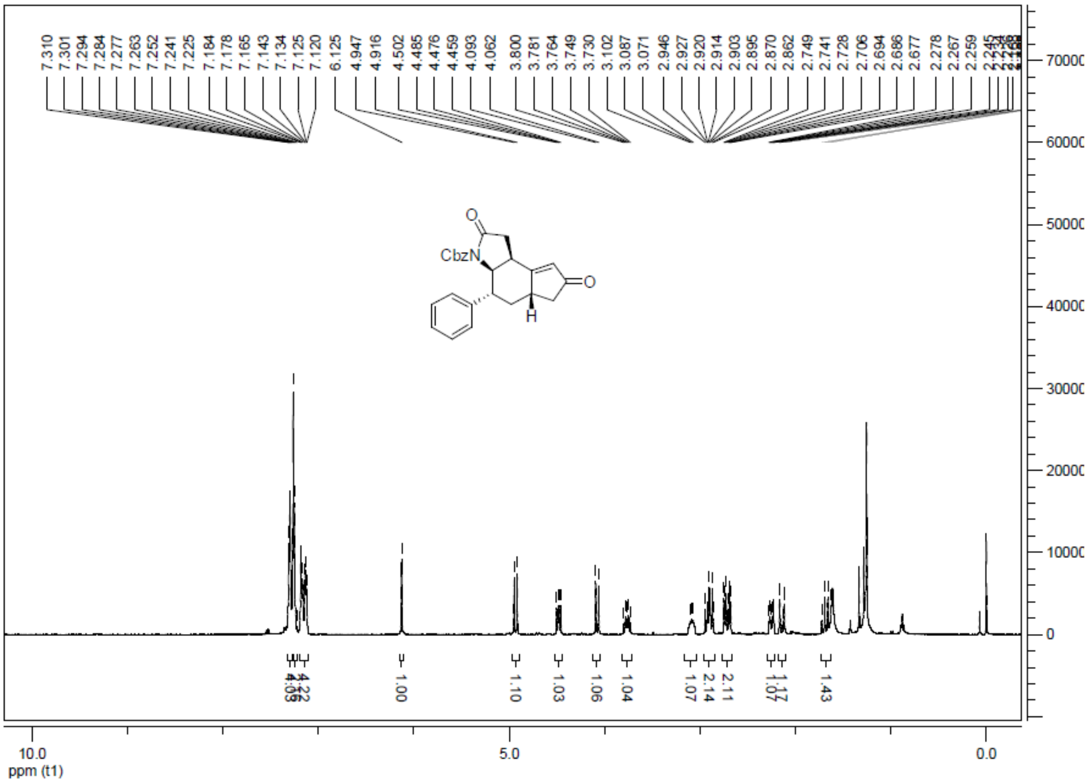

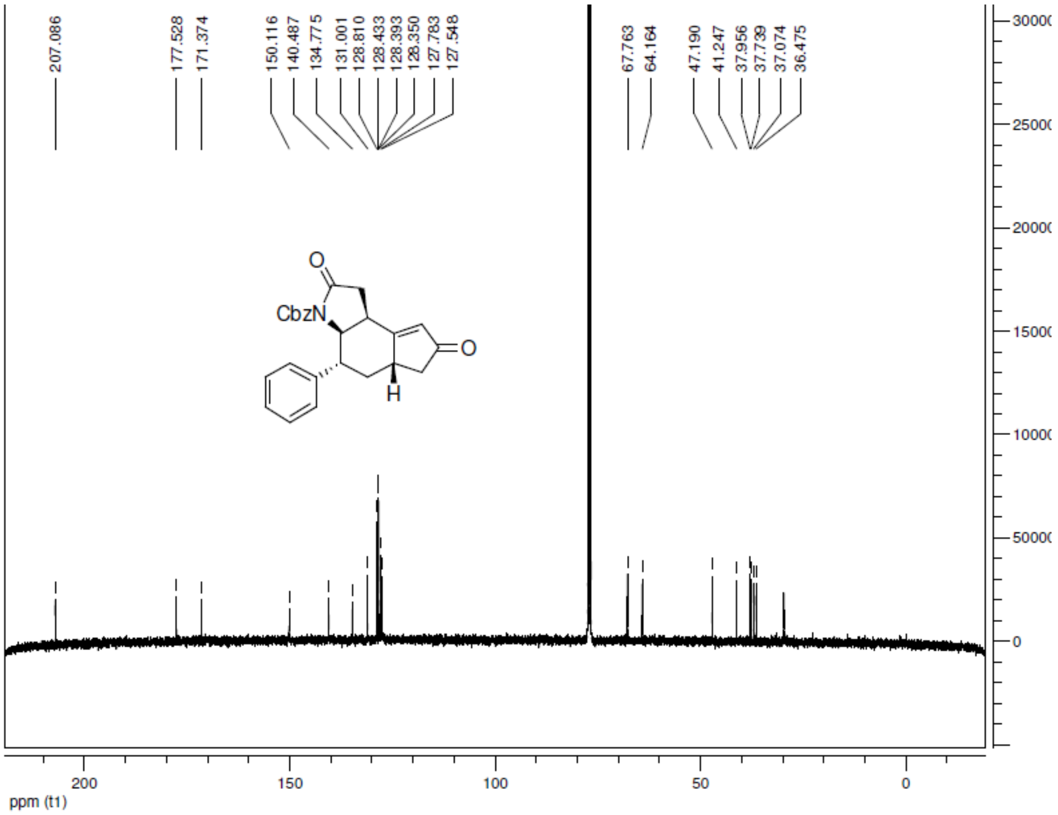

***tert*-Butyl 2-oxo-4-(4-oxo-2-phenylcyclopent-2-enyl)pyrrolidine-1-carboxylate (8a, diastereoisomer 1)**

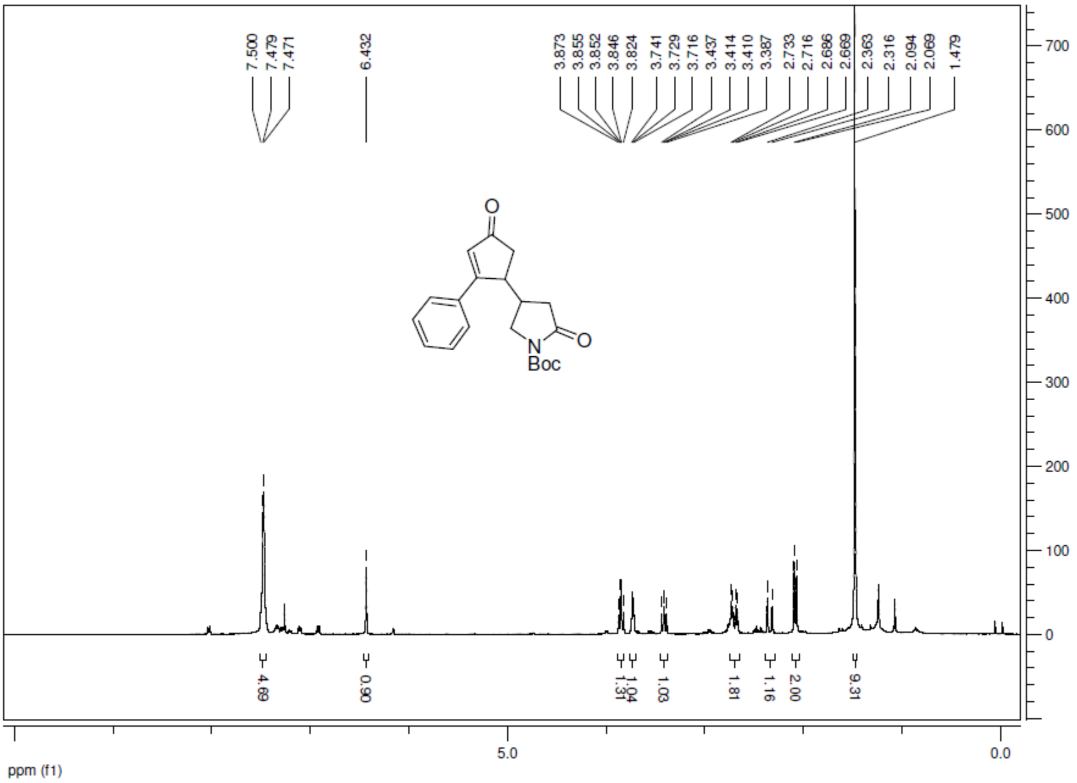

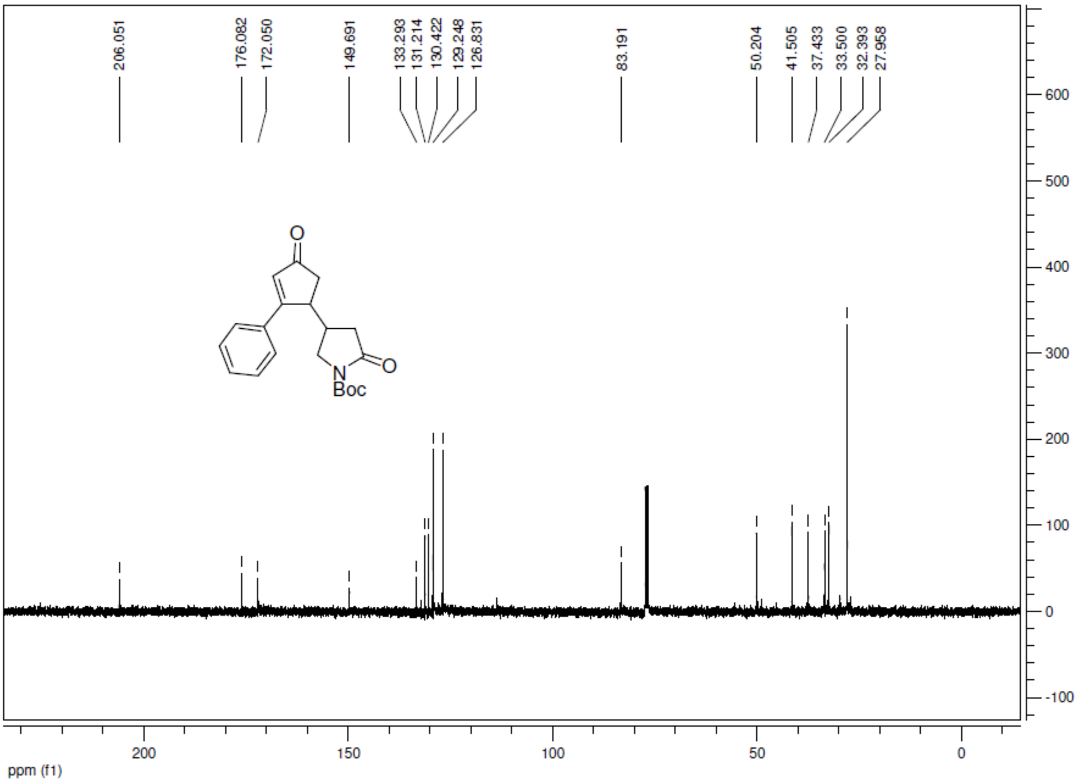

***tert*-Butyl 2-oxo-4-(4-oxo-2-phenylcyclopent-2-enyl)pyrrolidine-1-carboxylate (8a, diastereoisomer 2)**

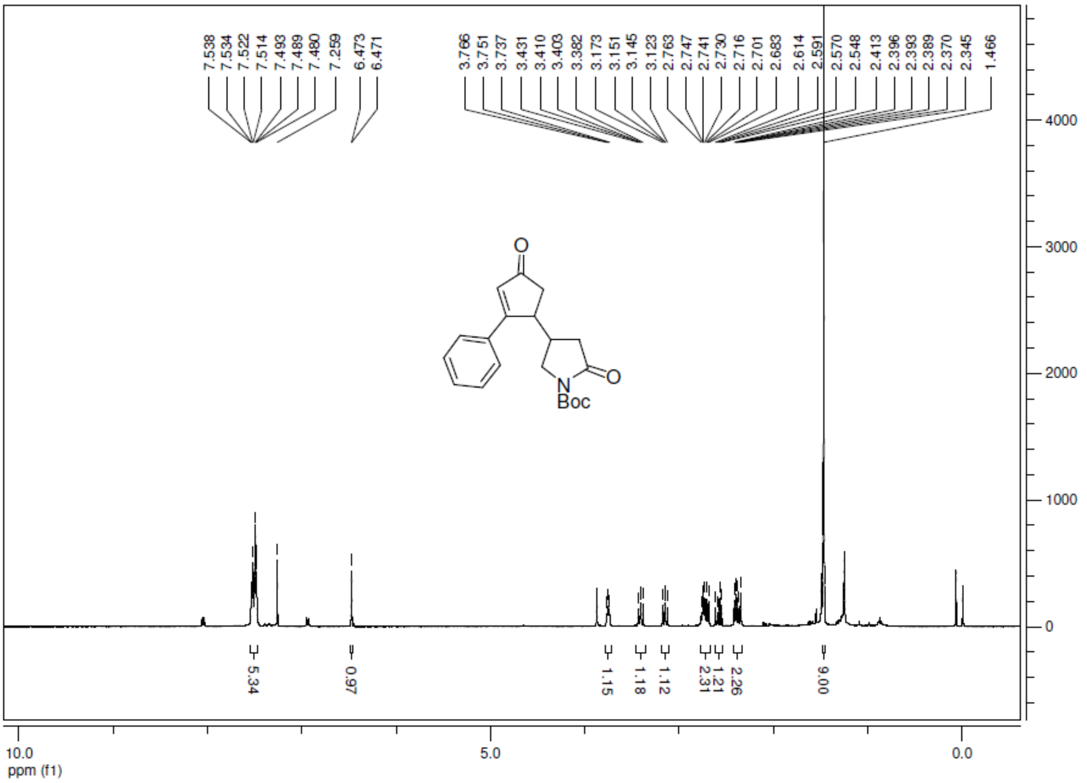

7.538
7.534
7.522
7.514
7.493
7.489
7.480
7.259
6.473
6.471
3.766
3.751
3.737
3.431
3.410
3.403
3.382
3.173
3.151
3.145
3.123
2.763
2.747
2.741
2.730
2.716
2.701
2.683
2.614
2.591
2.570
2.548
2.413
2.396
2.393
2.389
2.370
2.345
1.466
O
N
Boc
O
5.34
0.97
1.15
1.18
1.12
2.31
1.21
2.26
9.00
4000
3000
2000
1000
0
10.0
ppm (f1)
5.0
0.0

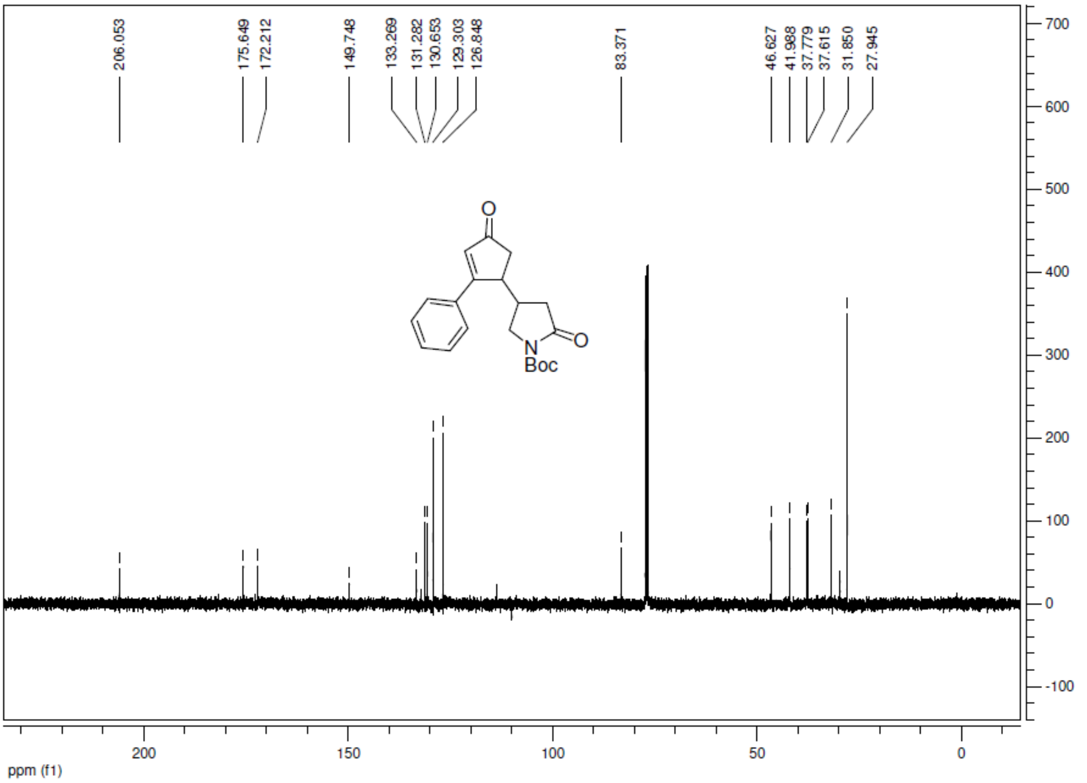

206.053
175.649
172.212
149.748
133.269
131.282
130.653
129.303
126.848
83.371
46.627
41.988
37.779
37.615
31.850
27.945
O
N
Boc
O
700
600
500
400
300
200
100
0
-100
200
150
100
50
0
ppm (f1)

F: HPLC Traces of Doubly Vinylogous Michael Adducts and Derivatives

***tert*-Butyl (*S*)-2-oxo-5-((*R*)-2-(3-oxocyclohex-1-enyl)-1-phenylethyl)-2,5-dihydro-1*H*-pyrrole-1-carboxylate (4a)**

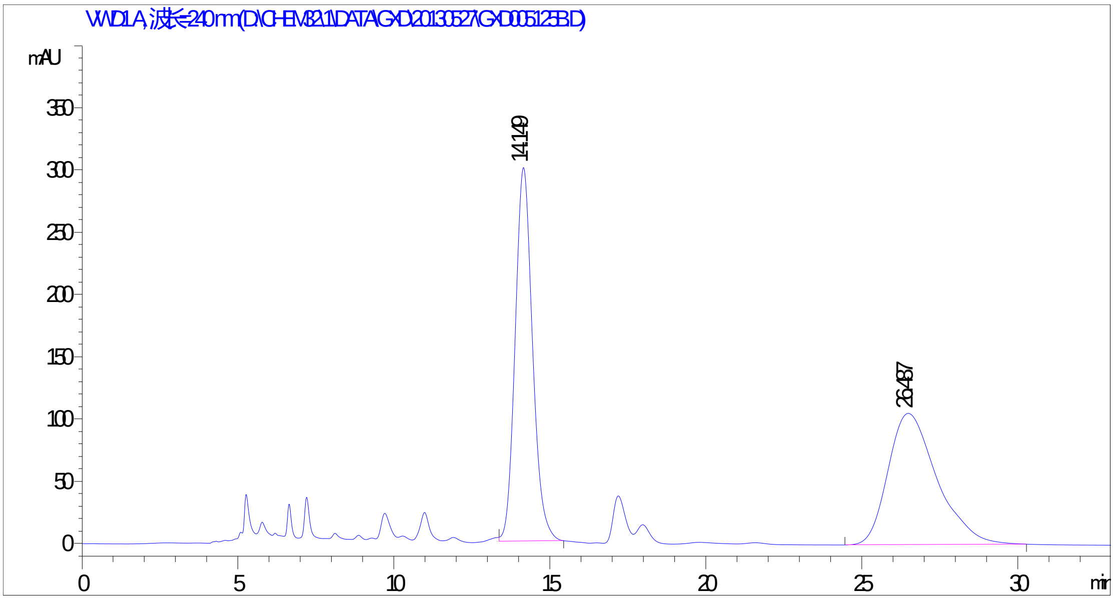

#	Time	Area	Height	Width	Symmetry	Area/%
1	14.149	11619.8	300.2	0.6451	0.881	50.217
2	26.487	11519.2	105.6	1.6749	0.667	49.783

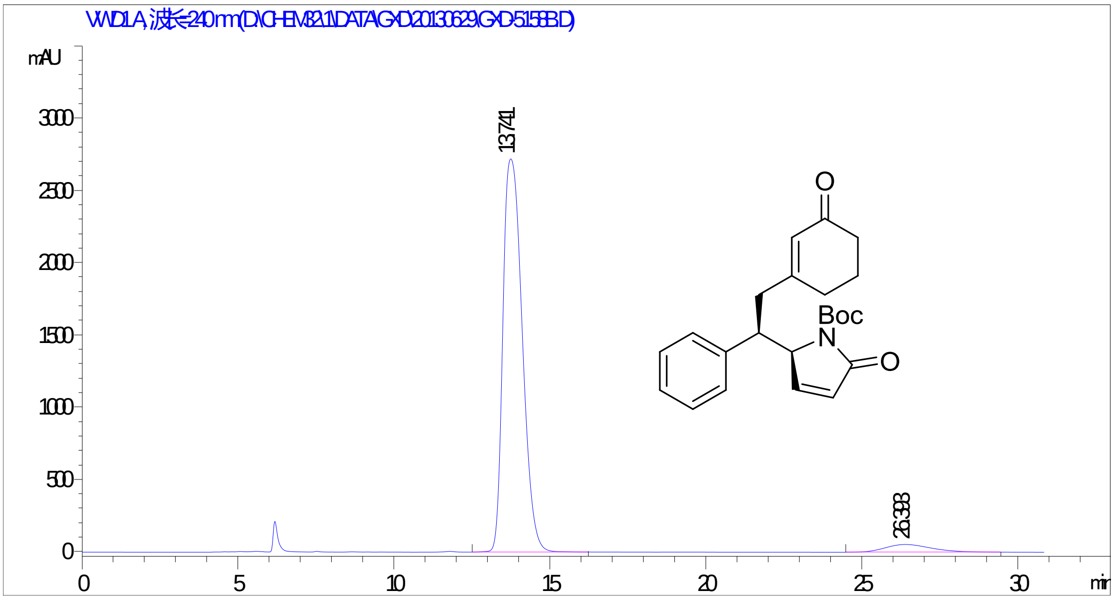

#	Time	Area	Height	Width	Symmetry	Area/%
1	13.741	116916.6	2720.2	0.6791	0.651	95.486
2	26.393	5526.6	53.3	1.5727	0.74	4.514

***tert*-Butyl (*S*)-2-oxo-5-((*R*)-2-(3-oxocyclohex-1-enyl)-1-*p*-tolylethyl)-2,5-dihydro-1*H*-pyrrole-1-carboxylate (4b)**

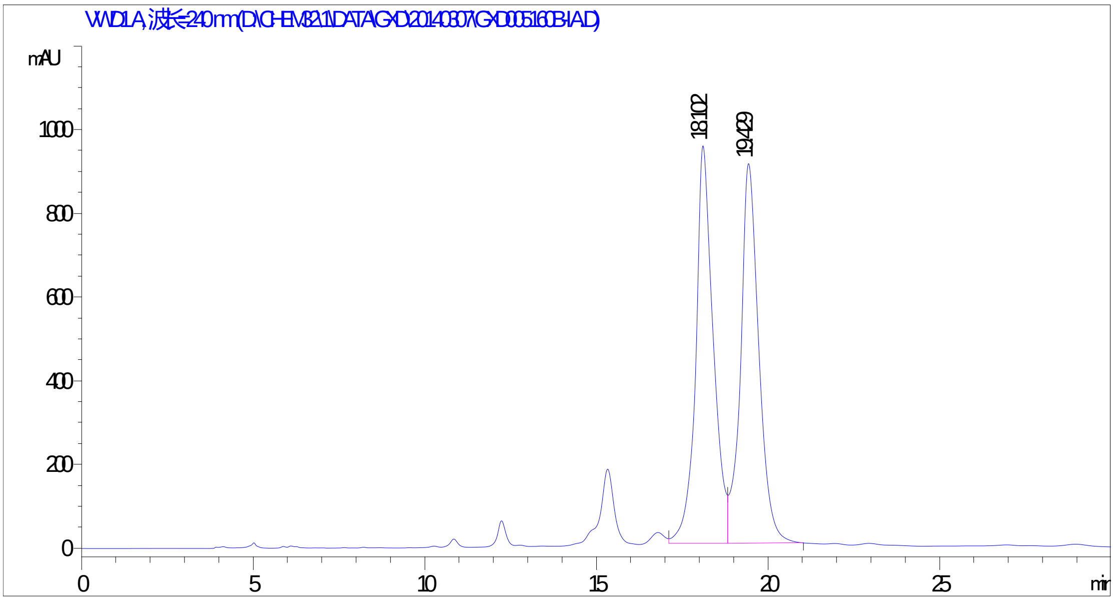

#	Time	Area	Height	Width	Symmetry	Area/%
1	18.102	33904.7	950.8	0.5943	0.722	50.228
2	19.429	33597.5	907.4	0.6171	0.76	49.772

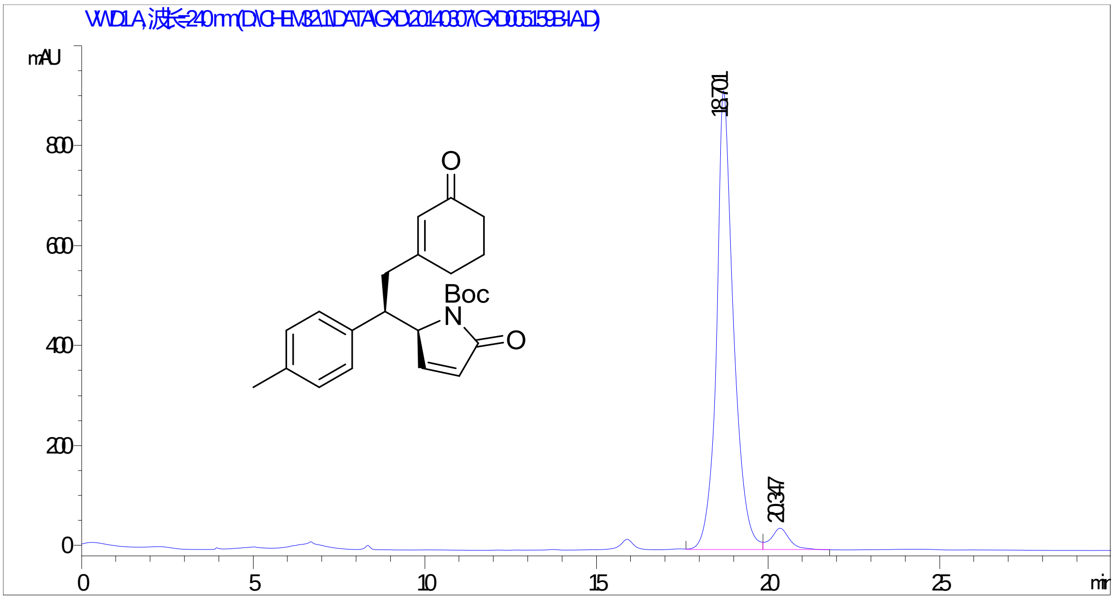

#	Time	Area	Height	Width	Symmetry	Area/%
1	18.701	31705.6	918.1	0.4946	0.693	95.012
2	20.347	1664.5	43.3	0.5586	0.892	4.988

tert-Butyl (S)-2-oxo-5-((R)-2-(3-oxocyclohex-1-enyl)-4-tert-butylphenyl)-2,5-dihydro- 1H-pyrrole-1-carboxylate (4c)

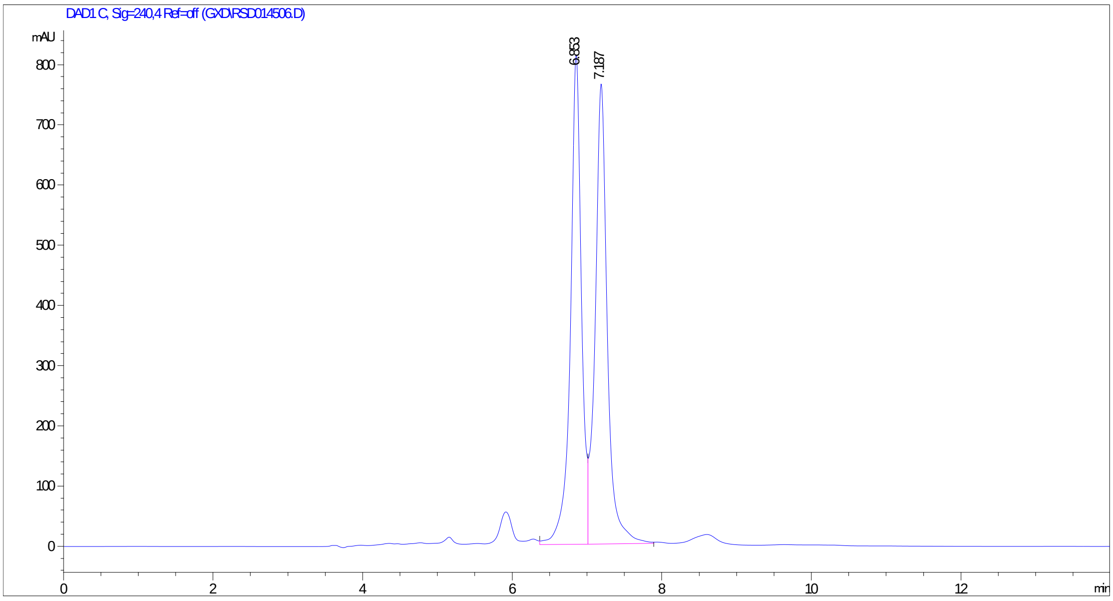

#	Time	Area	Height	Width	Symmetry	Area/%
1	6.853	8297.3	814.2	0.1469	1.105	49.681
2	7.187	8403.8	765.7	0.1829	0.932	50.319

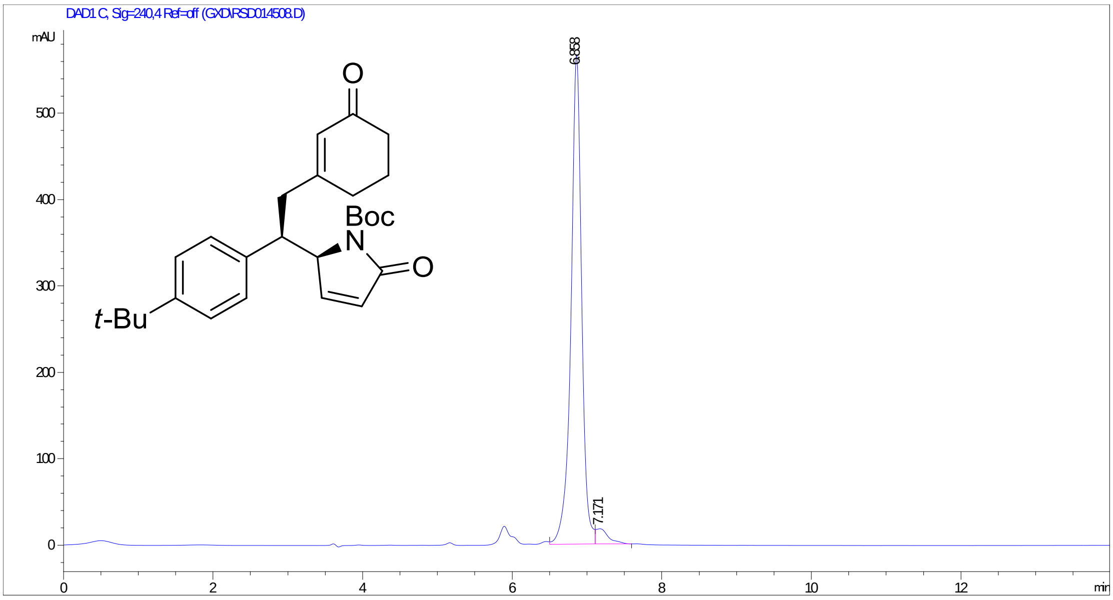

#	Time	Area	Height	Width	Symmetry	Area/%
1	6.858	5619.6	567.2	0.1456	1.076	96.408
2	7.171	209.4	17.9	0.1706	0.407	3.592

***tert*-Butyl (*S*)-2-oxo-5-((*R*)-2-(3-oxocyclohex-1-enyl)-1-*o*-tolylethyl)-2,5-dihydro-1*H*-pyrrole-1-carboxylate (4d)**

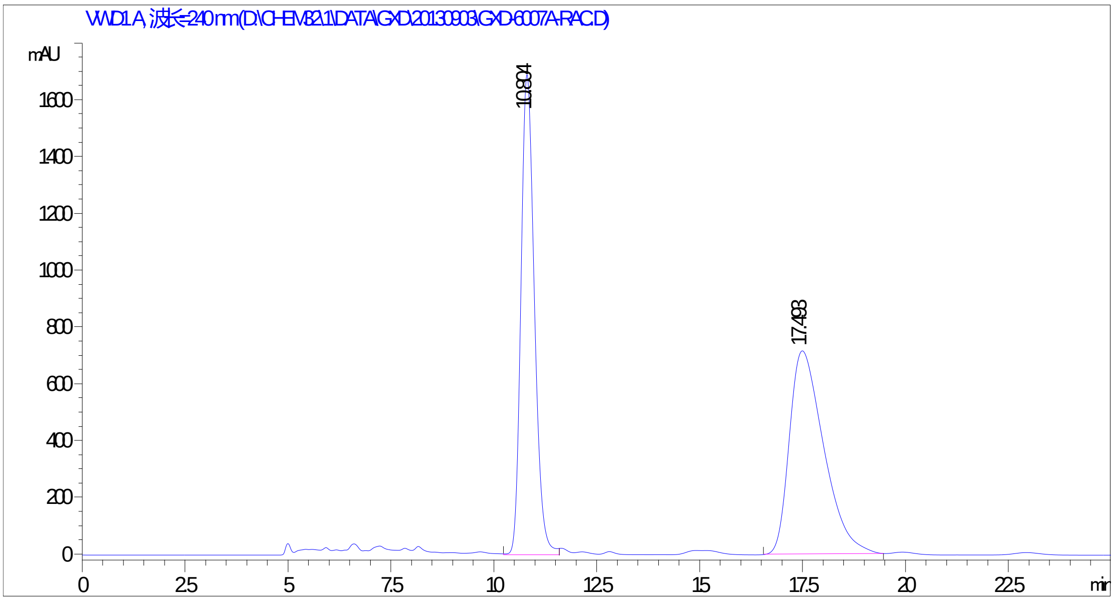

#	Time	Area	Height	Width	Symmetry	Area/%
1	10.804	38509.6	1691.6	0.3794	0.78	48.055
2	17.493	41627.7	716.5	0.9683	0.588	51.945

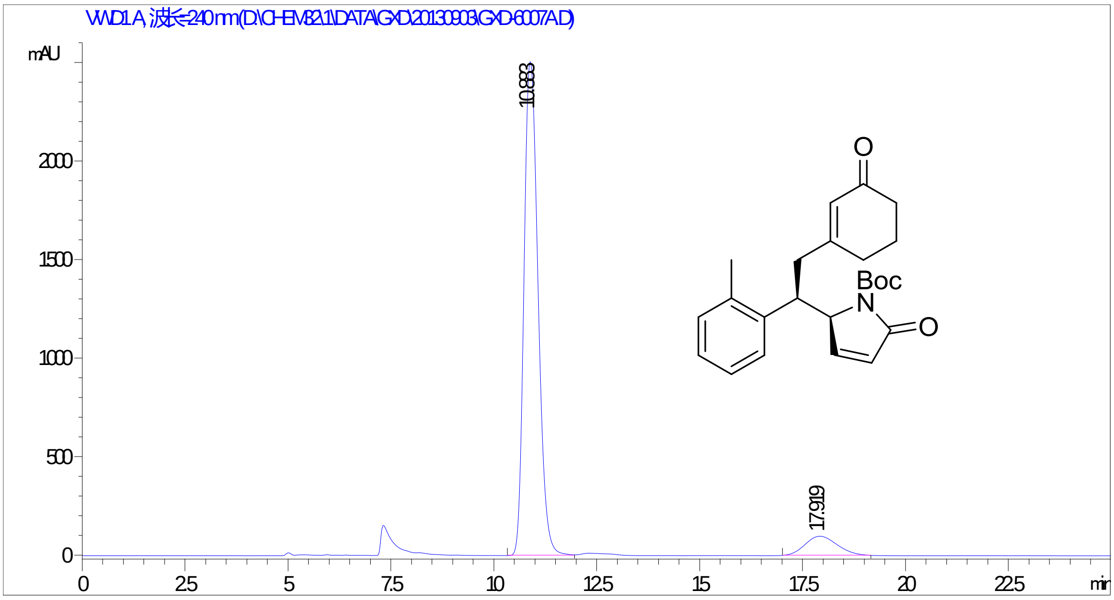

#	Time	Area	Height	Width	Symmetry	Area/%
1	10.883	59481.2	2505.9	0.3701	0.75	91.724
2	17.919	5366.6	97.8	0.9146	0.803	8.276

***tert*-Butyl (*S*)-2-oxo-5-((*R*)-2-(3-oxocyclohex-1-en-1-yl)-1-(2-(pivaloyloxy)phenyl) ethyl)-2,5-dihydro-1*H*-pyrrole-1-carboxylate (4e)**

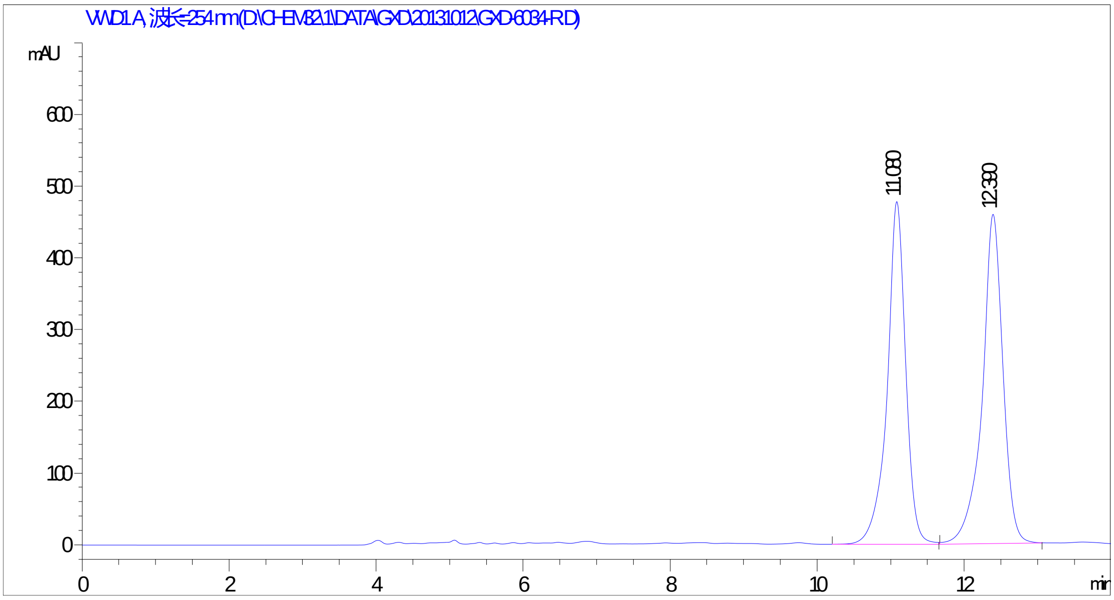

#	Time	Area	Height	Width	Symmetry	Area/%
1	11.08	8391.4	478.1	0.2586	1.077	48.238
2	12.39	9004.6	459.4	0.3267	1.076	51.762

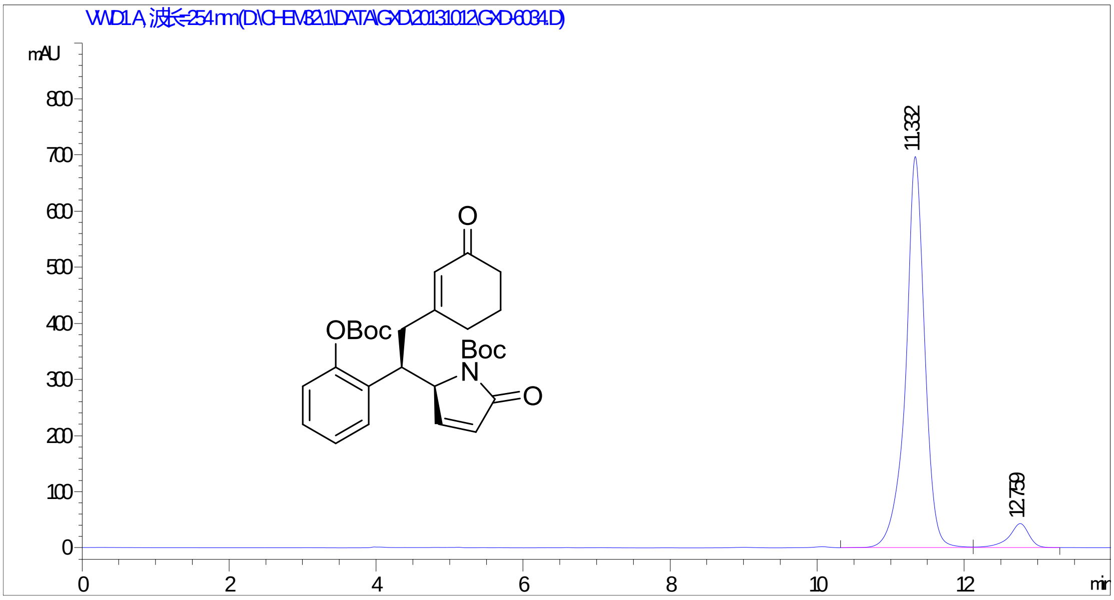

#	Time	Area	Height	Width	Symmetry	Area/%
1	11.332	12484.5	697.1	0.2628	1.01	93.795
2	12.759	825.9	42.9	0.2844	1.349	6.205

tert-Butyl (_S_)-2-((_R_)-1-(3,4-dimethoxyphenyl)-2-(3-oxocyclohex-1-en-1-yl)ethyl)-5-oxo-2,5-dihydro-1_H_-pyrrole-1-carboxylate (4f)

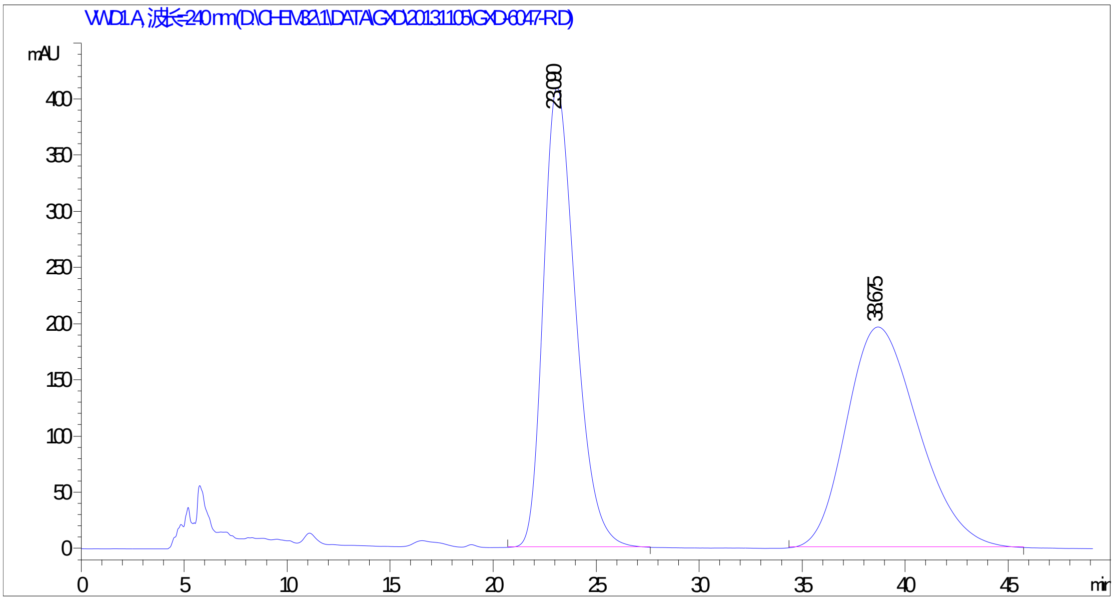

#	Time	Area	Height	Width	Symmetry	Area/%
1	23.09	44511.9	409.5	1.6497	0.69	48.494
2	38.675	47276.6	195.7	4.0255	0.758	51.506

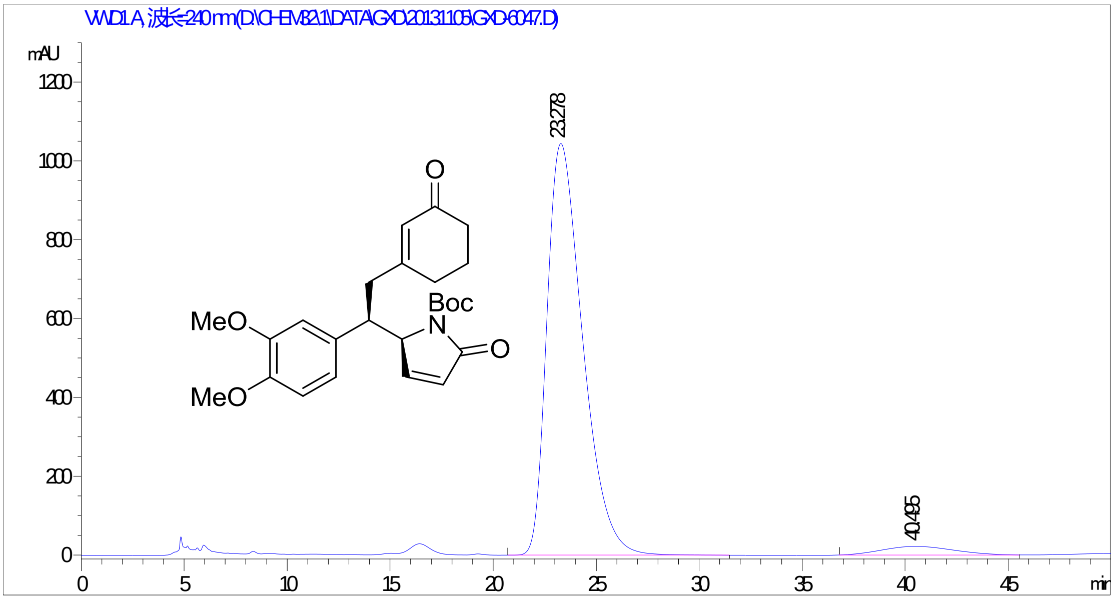

#	Time	Area	Height	Width	Symmetry	Area/%
1	23.278	126664.3	1045.2	2.0198	0.61	95.811
2	40.495	5538.5	22.3	4.1438	0.845	4.189

***tert*-Butyl (*S*)-2-((*R*)-1-(4-nitrophenyl)-2-(3-oxocyclohex-1-enyl)ethyl)-5-oxo- 2,5-dihydro-1*H*-pyrrole-1-carboxylate (4g)**

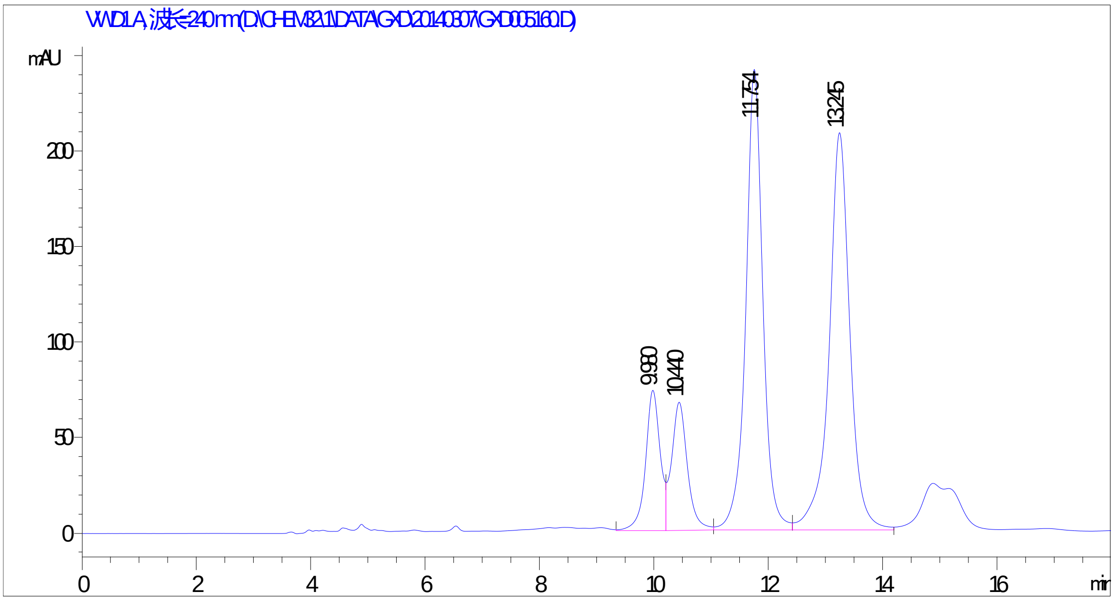

#	Time	Area	Height	Width	Symmetry	Area/%
1	9.98	1317.3	73.5	0.266	1.008	10.380
2	10.44	1299.2	67.2	0.2867	0.838	10.238
3	11.754	5031.4	241.2	0.3104	0.969	39.648
4	13.245	5042.3	208	0.4041	0.996	39.734

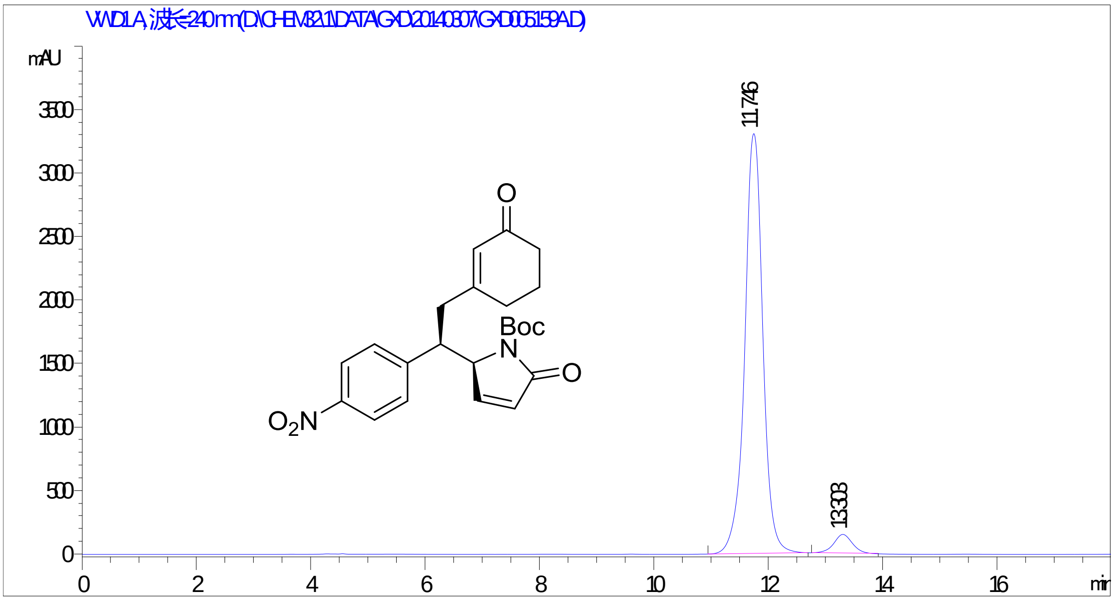

#	Time	Area	Height	Width	Symmetry	Area/%
1	11.746	72610.1	3306	0.3661	0.982	95.478
2	13.303	3438.5	149.1	0.3844	0.924	4.522

***tert*-Butyl (*S*)-2-((*R*)-1-(3-bromophenyl)-2-(3-oxocyclohex-1-enyl)ethyl)-5-oxo-2,5-dihydro-1*H*-pyrrole-1-carboxylate (4h)**

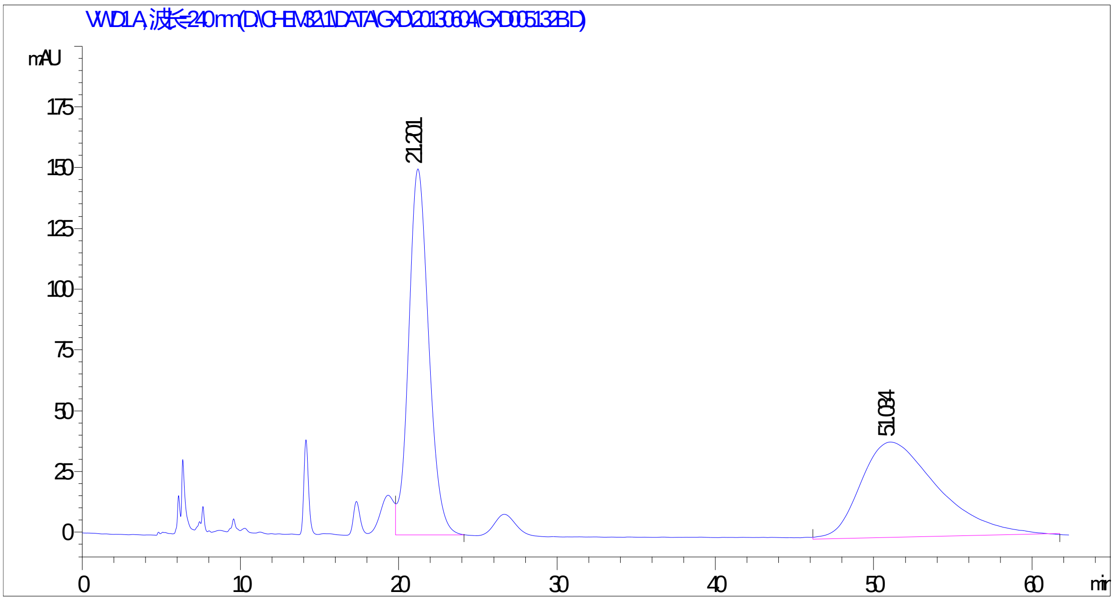

#	Time	Area	Height	Width	Symmetry	Area/%
1	21.201	13255.4	150.6	1.349	0.812	49.951
2	51.034	13281.5	39	5.6796	0.583	50.049

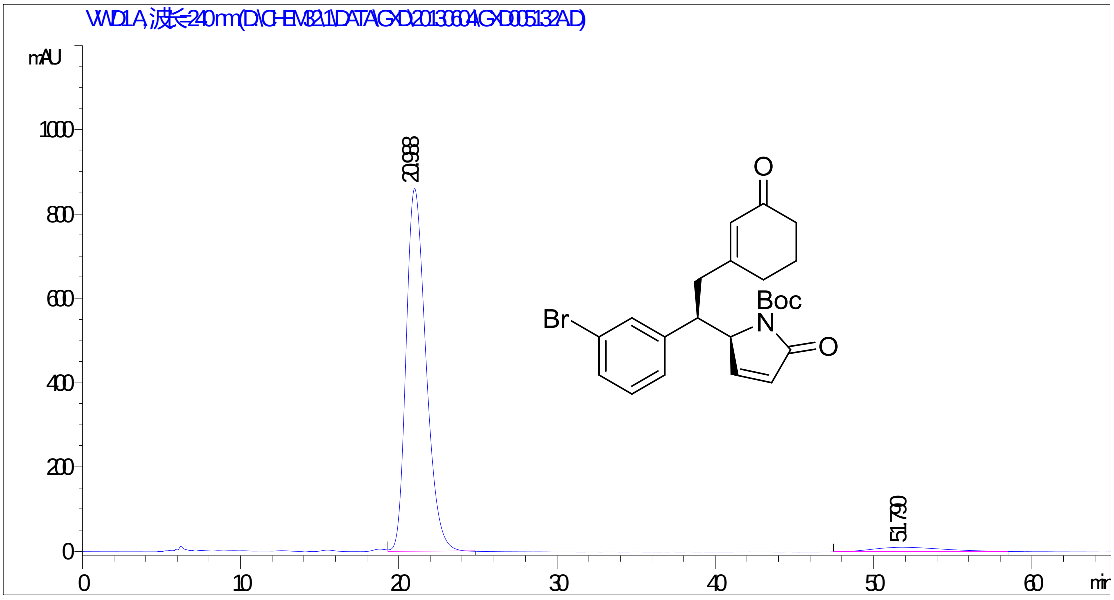

#	Time	Area	Height	Width	Symmetry	Area/%
1	20.988	75104.4	861	1.3366	0.708	95.921
2	51.790	3193.4	10.3	5.1461	0.653	4.079

tert-Butyl (_S_)-2-((_R_)-1-(4-fluorophenyl)-2-(3-oxocyclohex-1-enyl)ethyl)-5-oxo- 2,5-dihydro-1_H_-pyrrole-1-carboxylate (4i)

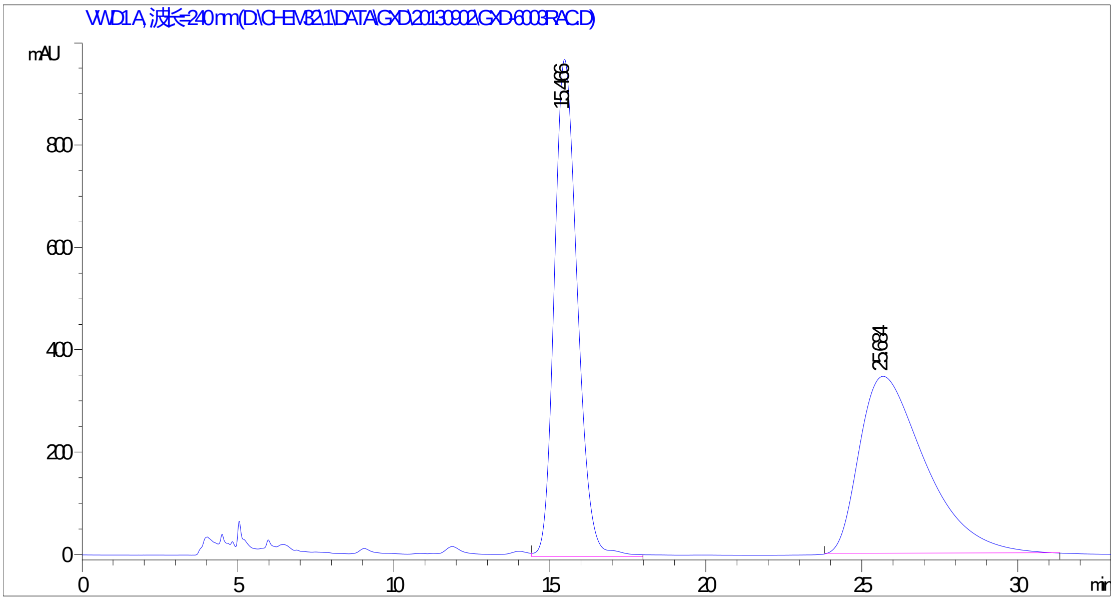

#	Time	Area	Height	Width	Symmetry	Area/%
1	15.466	49990	971.9	0.8572	0.769	49.455
2	25.684	51092.7	346.5	2.4574	0.546	50.545

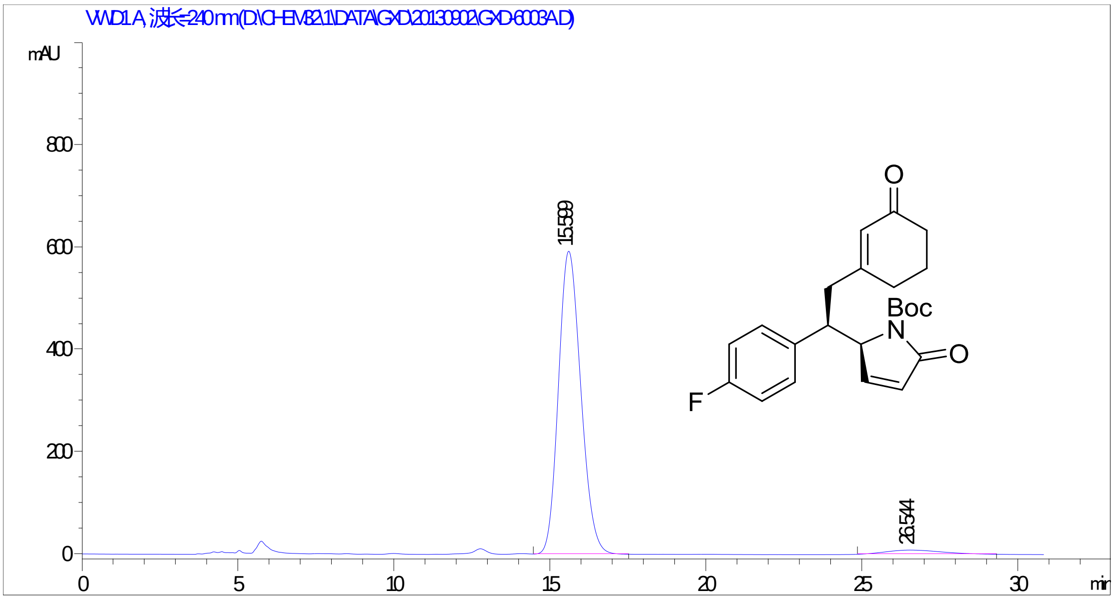

#	Time	Area	Height	Width	Symmetry	Area/%
1	15.599	29583.2	592.9	0.7772	0.809	96.718
2	26.544	1003.7	8.1	2.0593	0.733	3.282

tert-Butyl (_S_)-2-((_R_)-1-(4-chlorophenyl)-2-(3-oxocyclohex-1-enyl)ethyl)-5-oxo- 2,5-dihydro-1_H_-pyrrole-1-carboxylate (4j)

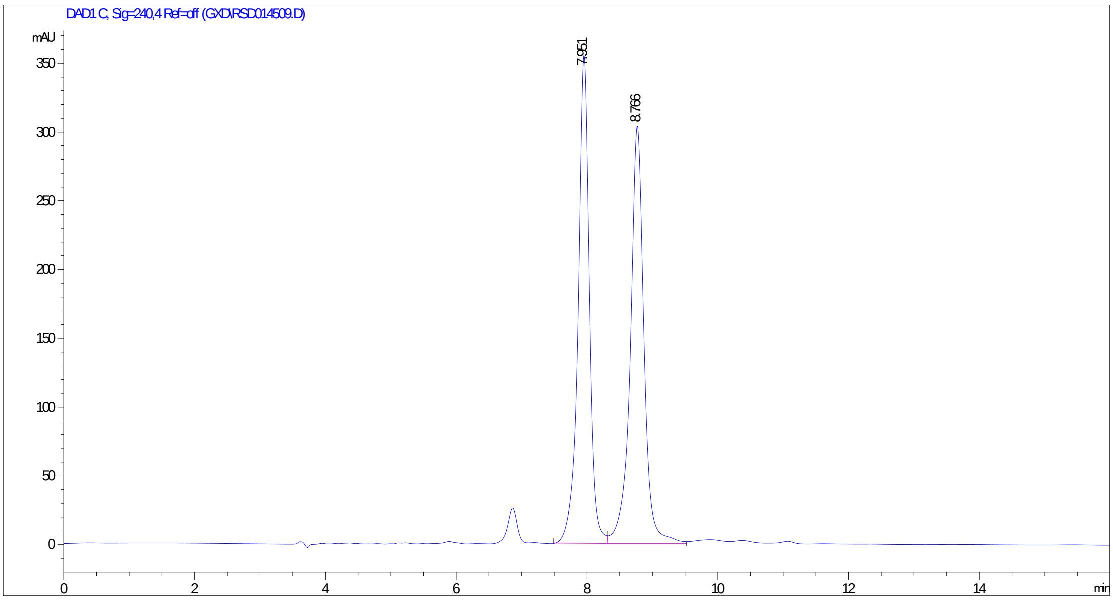

#	Time	Area	Height	Width	Symmetry	Area/%
1	7.951	4248.7	355.2	0.1776	1.075	49.753
2	8.766	4290.9	303.7	0.2355	1.037	50.247

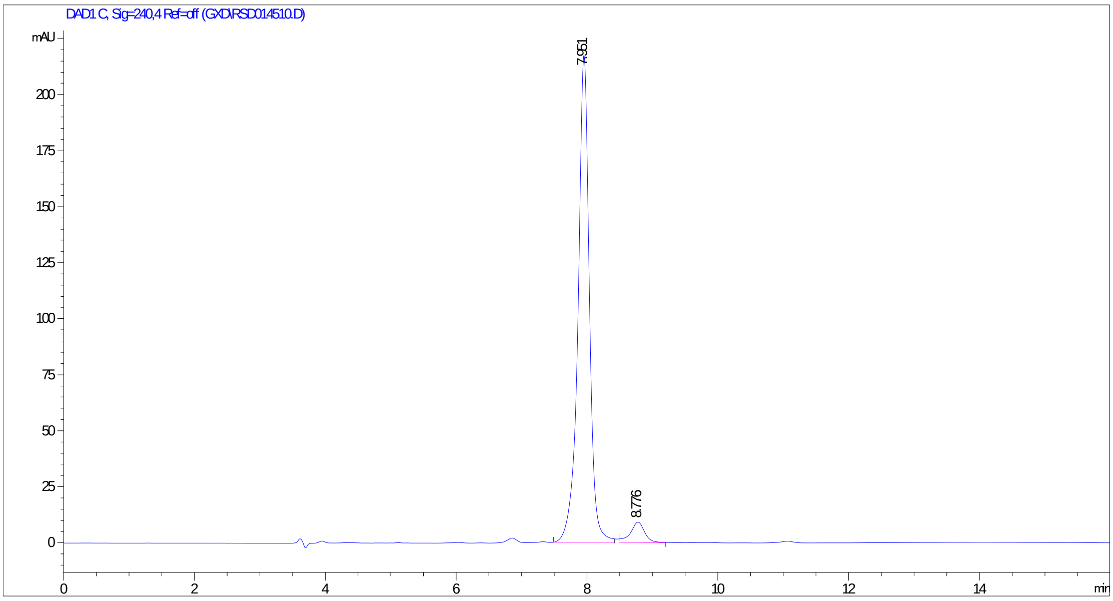

#	Time	Area	Height	Width	Symmetry	Area/%
1	7.951	2624.6	217.5	0.1788	1.074	95.001
2	8.776	138.1	9.1	0.2226	1.142	4.999

***tert*-Butyl (*R*)-2-oxo-5-((*S*)-1-(3-oxocyclohex-1-enyl)propan-2-yl)-2,5-dihydro- 1*H*-pyrrole-1-carboxylate (4k)**

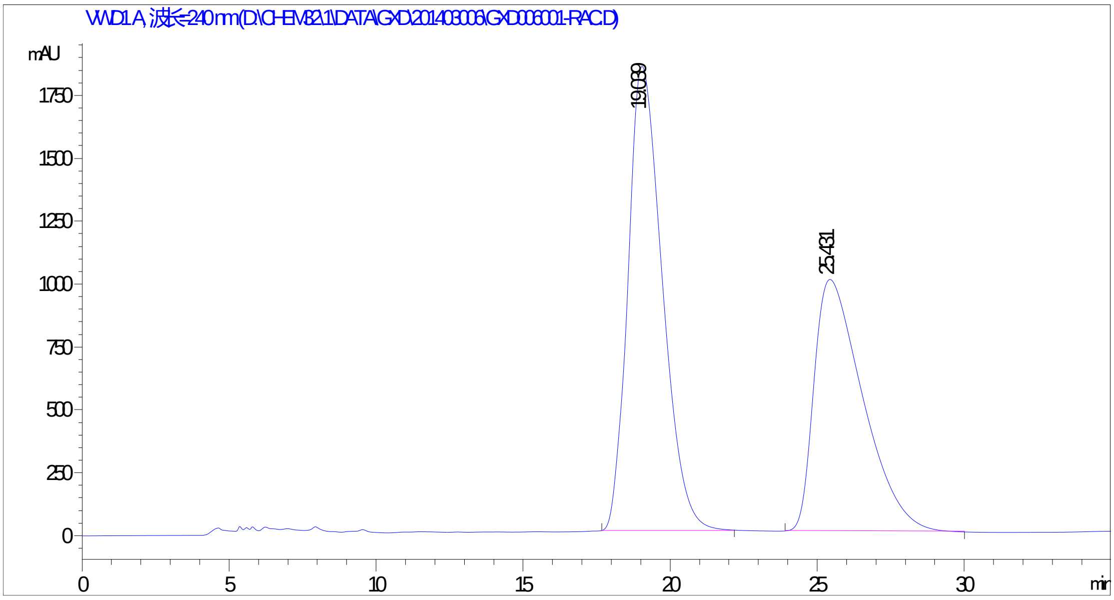

#	Time	Area	Height	Width	Symmetry	Area/%
1	19.039	120441	1858.6	1.08	0.483	50.536
2	25.431	117886.1	1007.9	1.7809	0.456	49.464

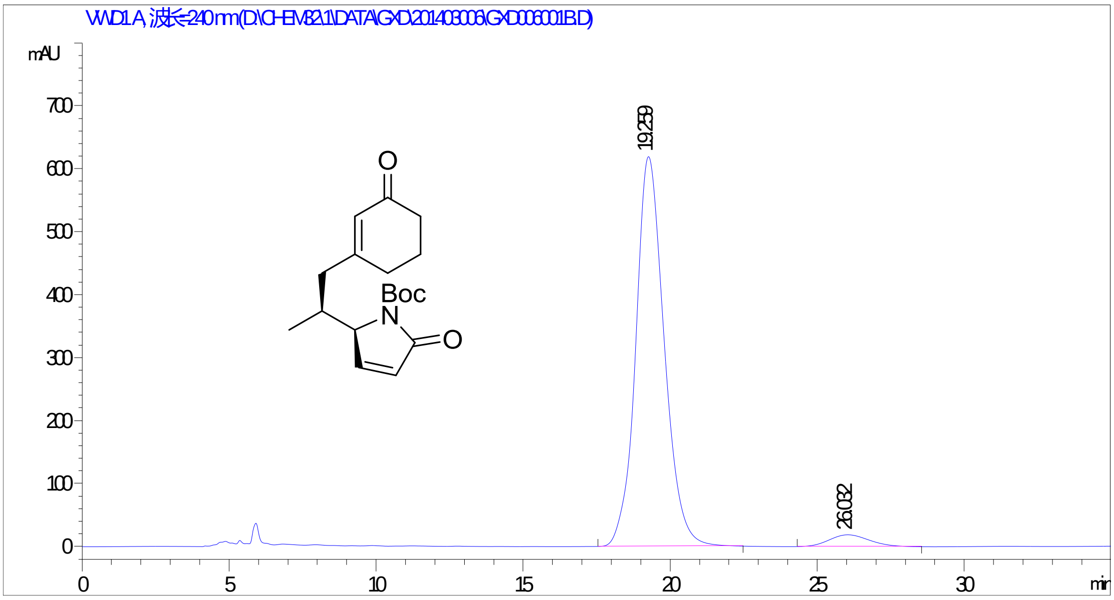

#	Time	Area	Height	Width	Symmetry	Area/%
1	19.259	41700.6	619	1.0263	0.787	95.863
2	26.032	1799.7	18.8	1.403	0.819	4.137

tert-Butyl (_R_)-2-oxo-5-((_S_)-1-(3-oxocyclohex-1-enyl)pentan-2-yl)-2,5-dihydro- 1_H_-pyrrole-1-carboxylate (4l)

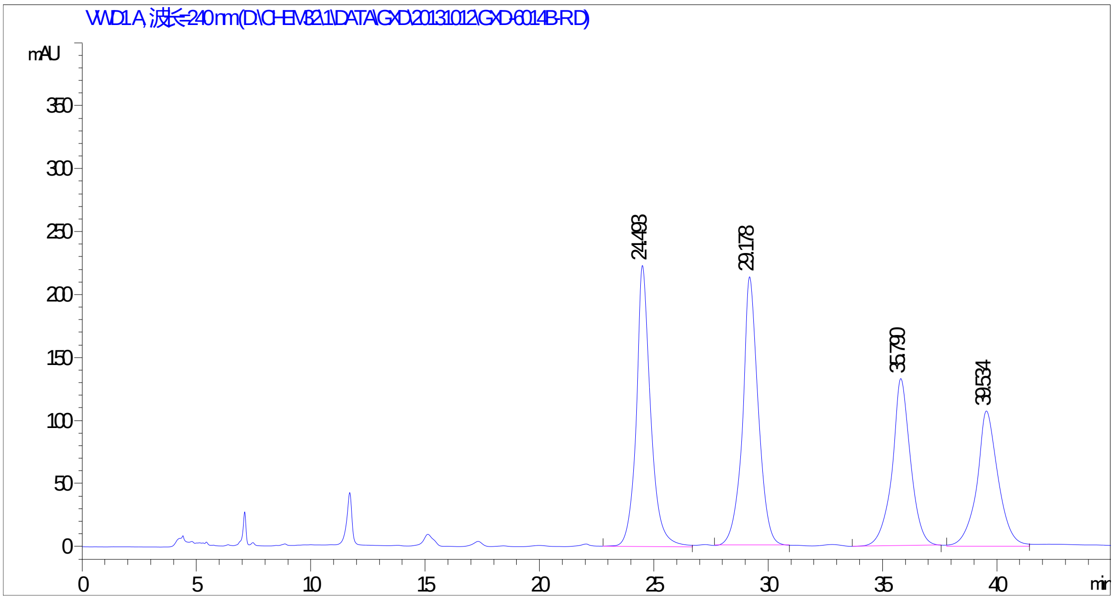

#	Time	Area	Height	Width	Symmetry	Area/%
1	24.493	9782.4	223.4	0.7299	0.73	28.540
2	29.178	10149.6	213.2	0.6908	0.809	29.611
3	35.79	7404.6	132.7	0.7937	0.897	21.603
4	39.534	6940	107.5	1.0764	0.808	20.247

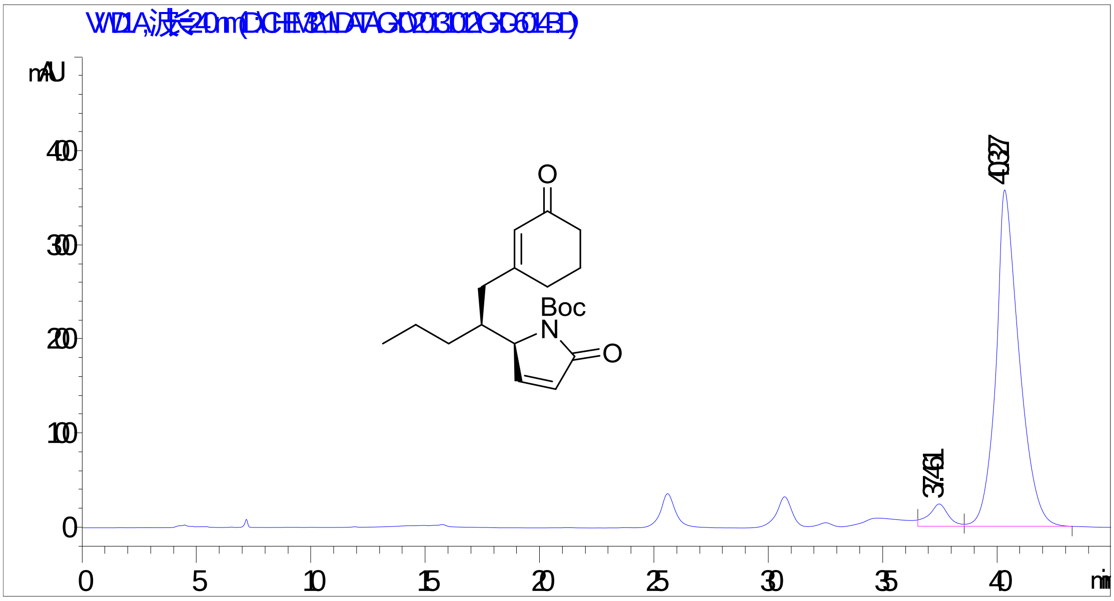

#	Time	Area	Height	Width	Symmetry	Area/%
1	37.461	1507.3	24.5	1.0246	1.11	5.600
2	40.327	25408.8	358.1	0.9909	0.617	94.400

***tert*-Butyl (*S*)-2-((*R*)-2-(5,5-dimethyl-3-oxocyclohex-1-enyl)-1-phenylethyl)-5-oxo-2,5-dihydro-1*H*-pyrrole-1-carboxylate (4m)**

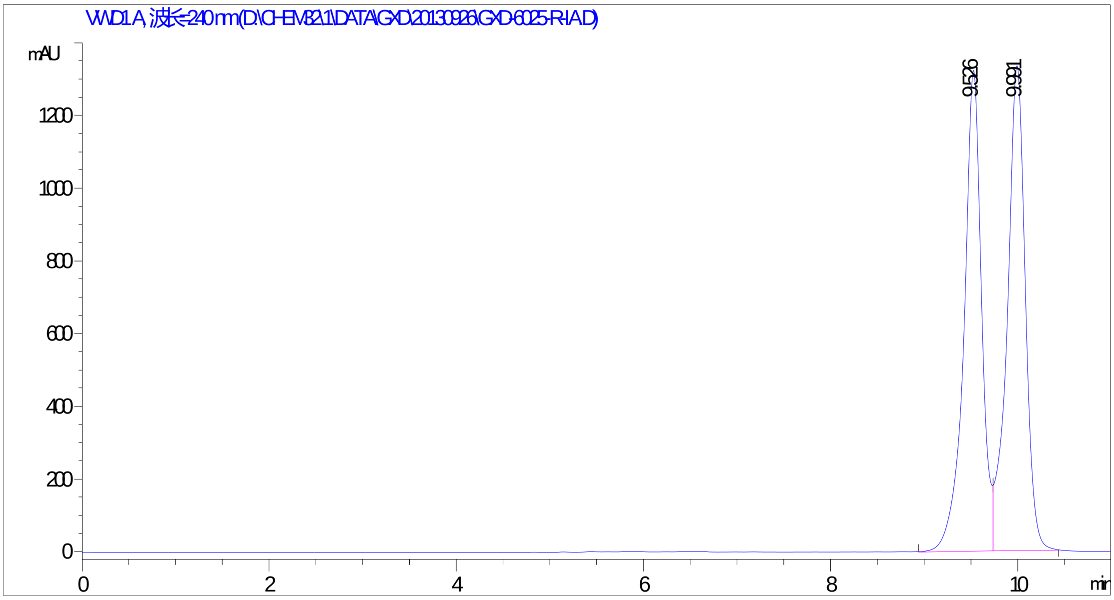

#	Time	Area	Height	Width	Symmetry	Area/%
1	9.526	17362.2	1323.1	0.2187	1.157	49.227
2	9.991	17907.6	1334.3	0.2237	1.123	50.773

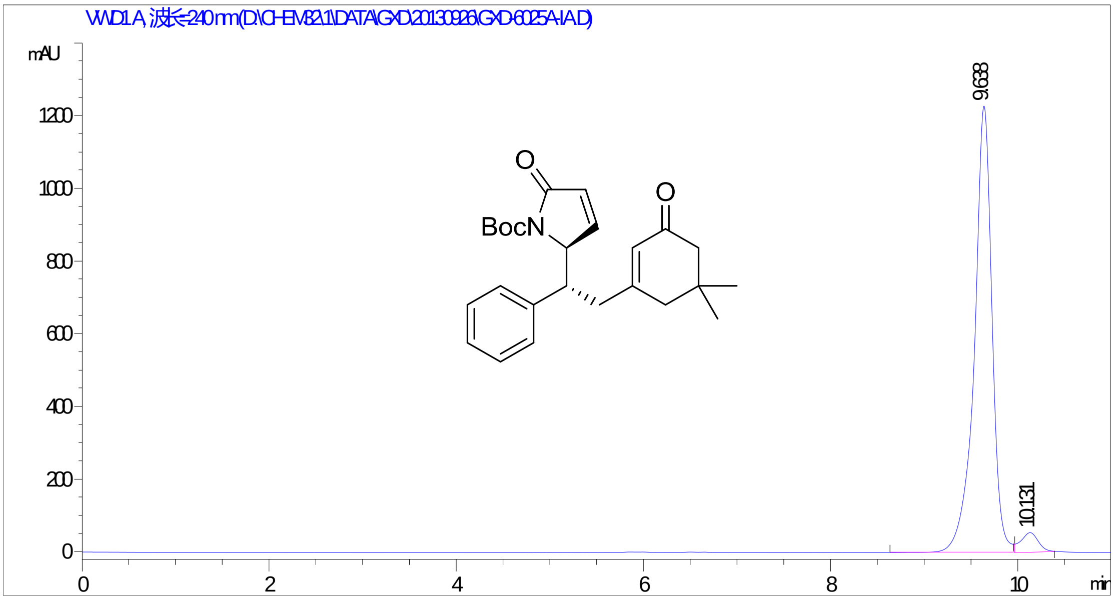

#	Time	Area	Height	Width	Symmetry	Area/%
1	9.638	16093.8	1228.3	0.1951	1.187	95.744
2	10.131	715.4	54.7	0.2181	1.066	4.256

***tert*-Butyl (*S*)-2-((*R*)-2-(6,6-dimethyl-3-oxocyclohex-1-enyl)-1-phenylethyl)-5-oxo-2,5-dihydro-1*H*-pyrrole-1-carboxylate (4n)**

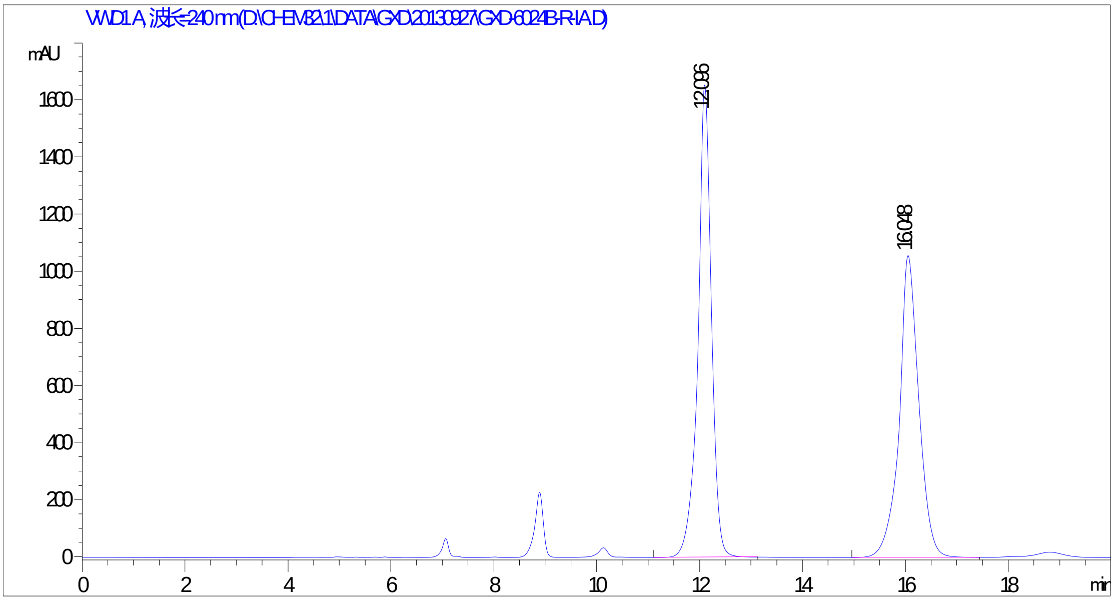

#	Time	Area	Height	Width	Symmetry	Area/%
1	12.096	29954.1	1653.9	0.2652	0.992	51.696
2	16.048	27988.4	1057.6	0.3815	0.825	48.304

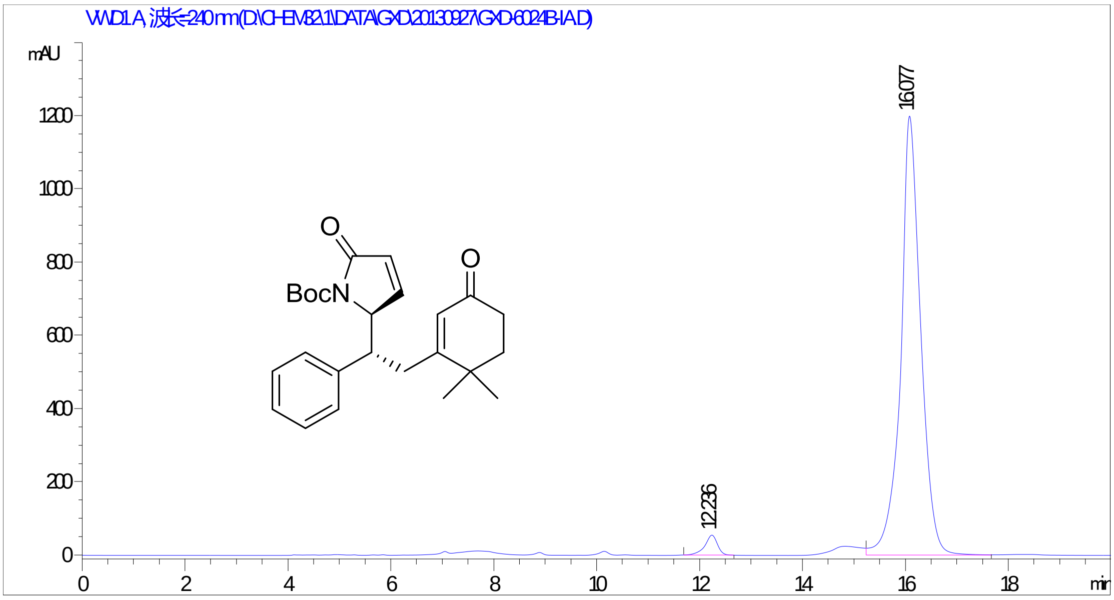

#	Time	Area	Height	Width	Symmetry	Area/%
1	12.236	950.8	55	0.2881	1.227	2.838
2	16.077	32551.4	1199.8	0.3908	0.804	97.162

tert-Butyl (S)-2-((R)-1-(naphthalen-2-yl)-2-(3-oxocyclohex-1-enyl)ethyl)-5-oxo-2,5-dihydro-1H-pyrrole-1-carboxylate (4o)

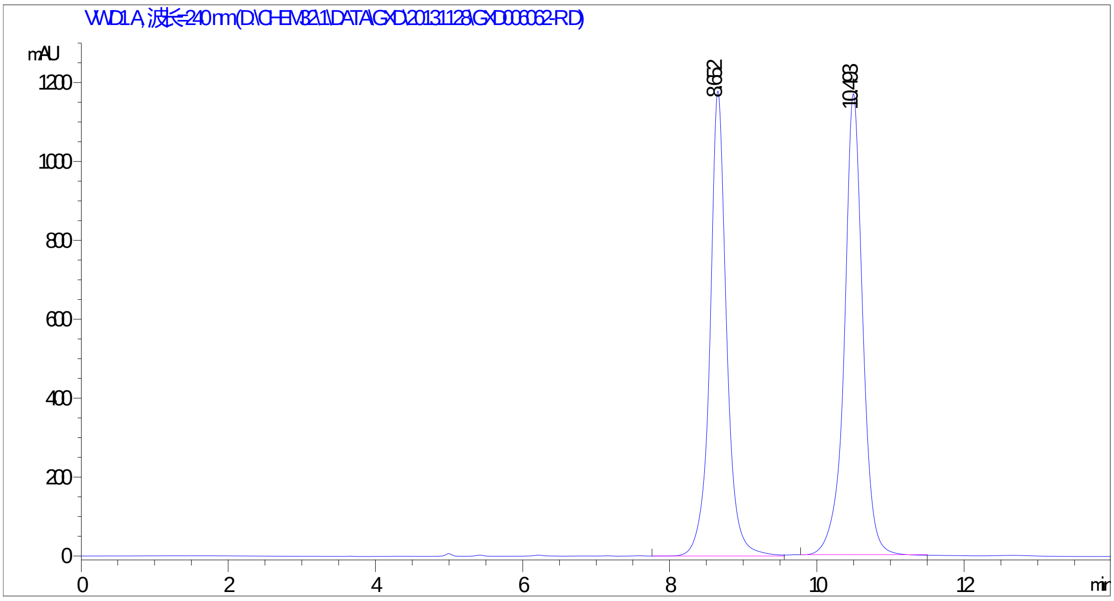

#	Time	Area	Height	Width	Symmetry	Area/%
1	8.652	18593.2	1179.3	0.232	0.88	47.841
2	10.493	20271.6	1169	0.289	0.943	52.159

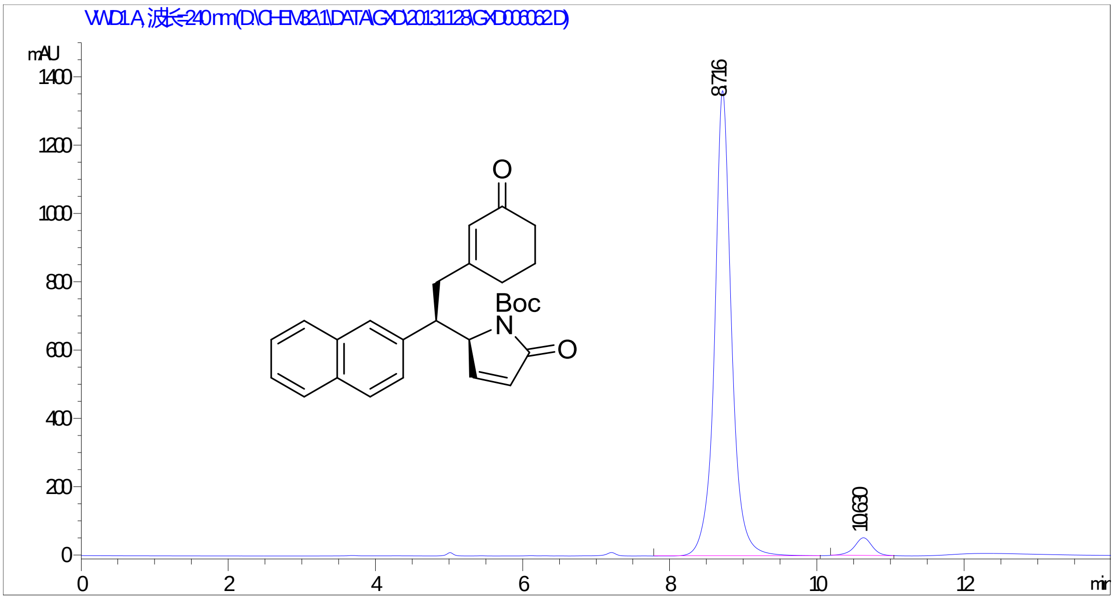

#	Time	Area	Height	Width	Symmetry	Area/%
1	8.716	21832.3	1361.2	0.2366	0.865	96.102
2	10.63	885.6	52.1	0.2831	1.015	3.898

tert-Butyl (_S_)-2-oxo-5-((_R_)-2-(3-oxocyclohex-1-enyl)-1-(thiophen-3-yl)ethyl)-2,5-dihydro-1_H_-pyrrole-1-carboxylate (4p)

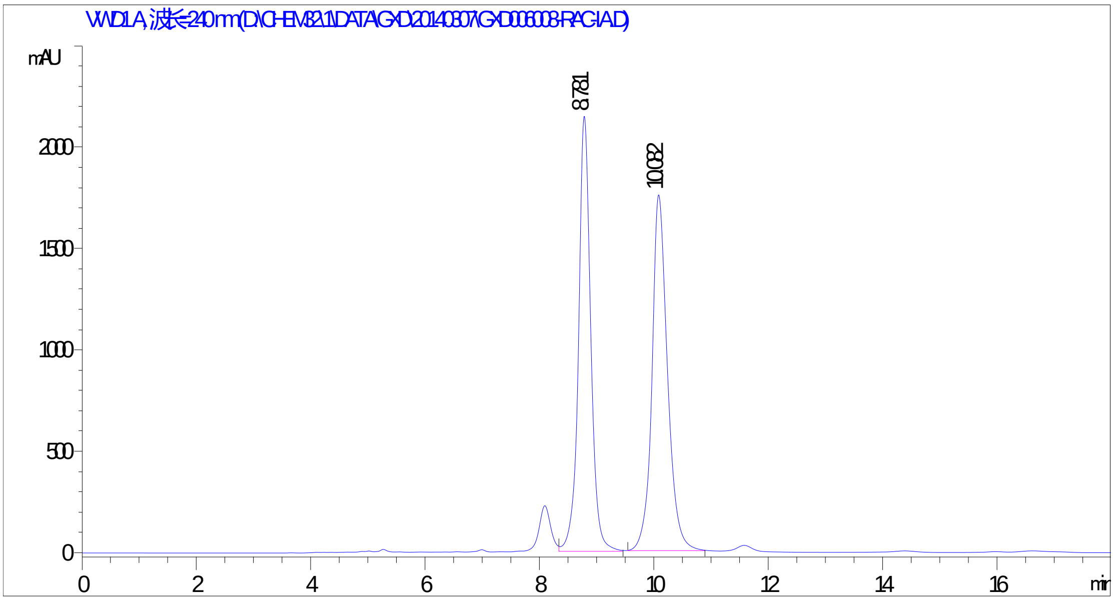

#	Time	Area	Height	Width	Symmetry	Area/%
1	8.781	31971	2147.3	0.2482	0.877	49.478
2	10.082	32645.8	1756.3	0.3098	0.771	50.522

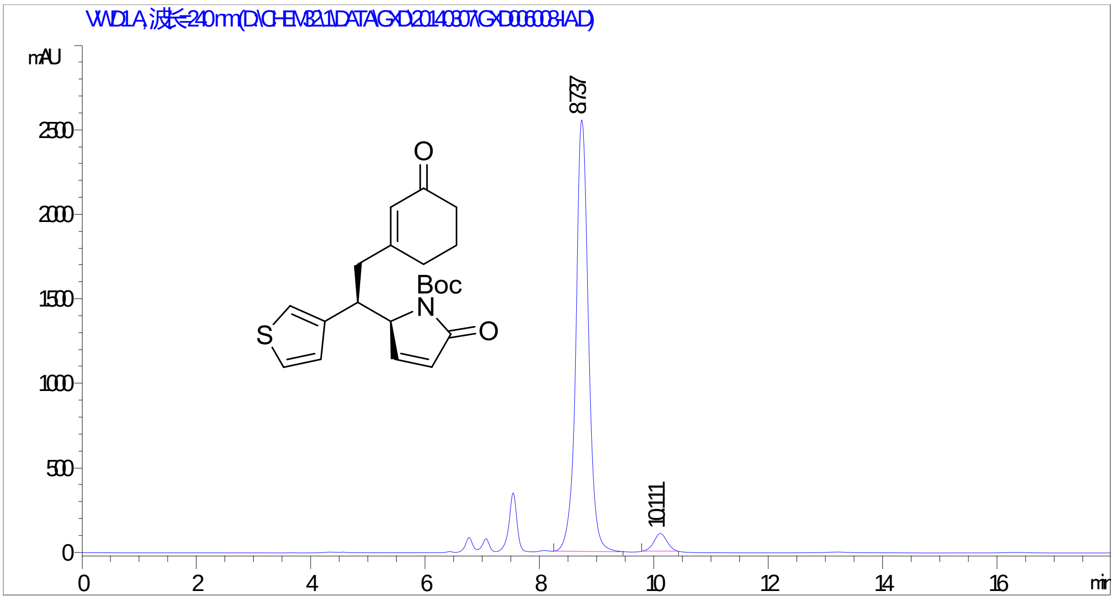

#	Time	Area	Height	Width	Symmetry	Area/%
1	8.737	39134.7	2553.8	0.2554	0.866	95.732
2	10.111	1744.7	106.7	0.2726	0.952	4.268

Benzyl (*S*)-2-oxo-5-((*R*)-2-(3-oxocyclohex-1-enyl)-1-phenylethyl)-2,5-dihydro- 1*H*-pyrrole-1-carboxylate (4q)

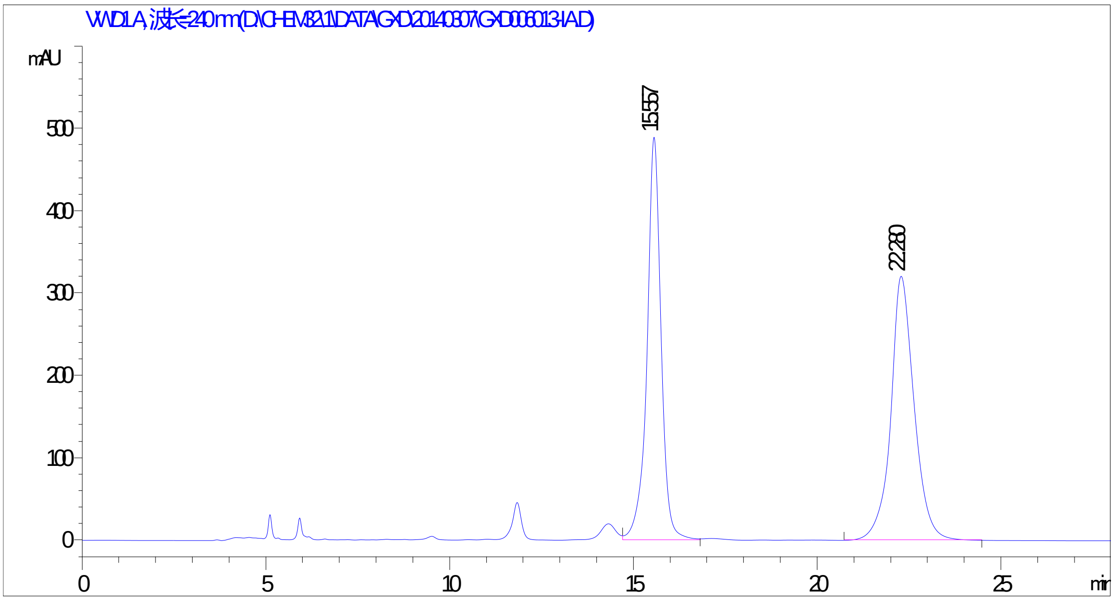

#	Time	Area	Height	Width	Symmetry	Area/%
1	15.557	13063.8	489.3	0.3959	0.989	49.989
2	22.28	13069.6	312.9	0.6962	0.793	50.011

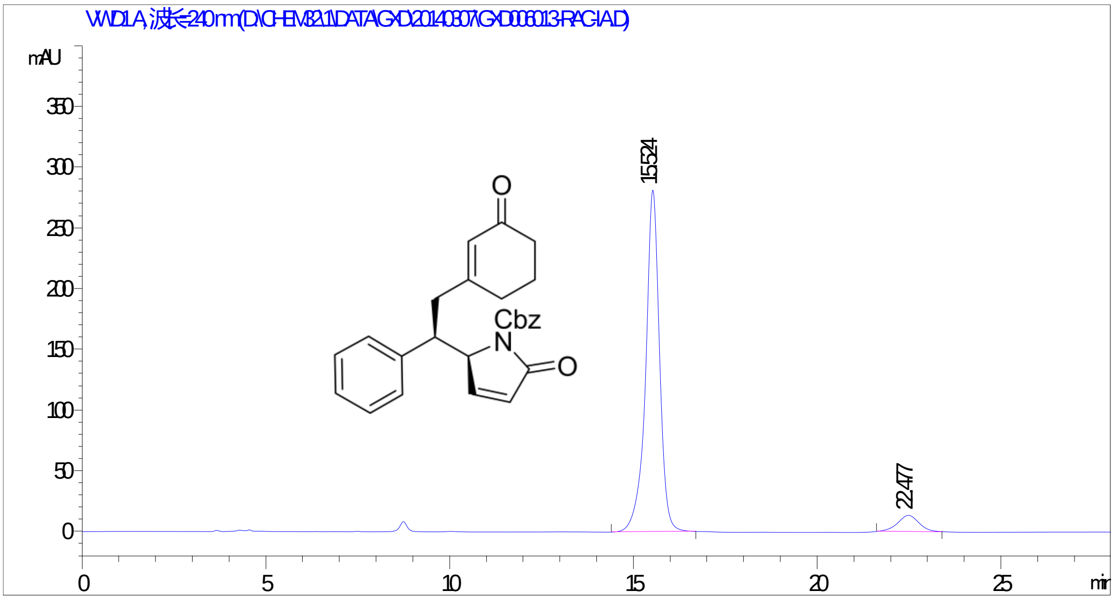

#	Time	Area	Height	Width	Symmetry	Area/%
1	15.524	7413.8	281.3	0.3919	1.068	95.048
2	22.477	386.3	11.5	0.56	1.014	4.952

Benzyl (*S*)-2-((*R*)-2-(5,5-dimethyl-3-oxocyclohex-1-enyl)-1-phenylethyl)-5-oxo-2,5-dihydro-1*H*-pyrrole-1-carboxylate (4r)

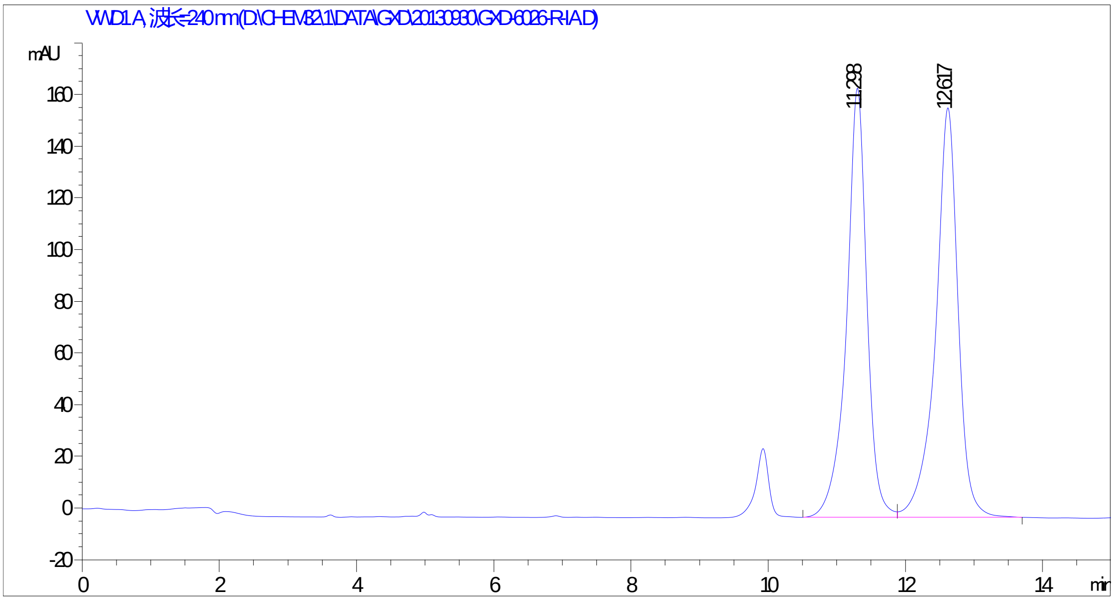

#	Time	Area	Height	Width	Symmetry	Area/%
1	11.298	3425.1	166.2	0.3031	1.123	48.667
2	12.617	3612.8	158.5	0.3343	1.185	51.333

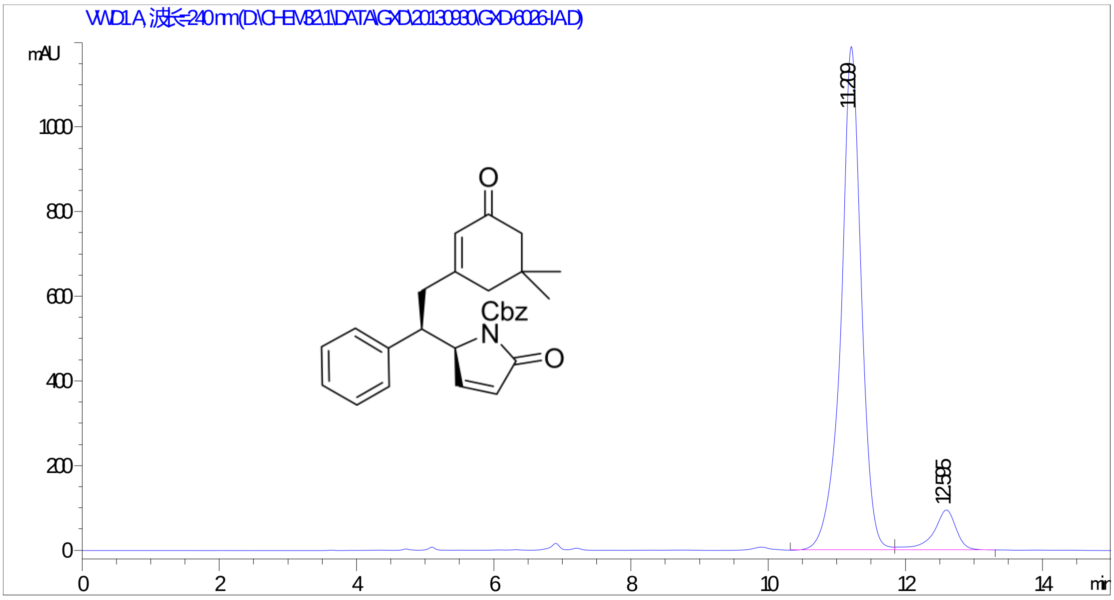

#	Time	Area	Height	Width	Symmetry	Area/%
1	11.209	24437.9	1190.2	0.3036	1.03	91.558
2	12.595	2253.2	95	0.3438	1.379	8.442

Benzyl (*S*)-2-((*R*)-1-(4-fluorophenyl)-2-(3-oxocyclohex-1-enyl)ethyl)-5-oxo-2,5-dihydro-1*H*-pyrrole-1-carboxylate (4s)

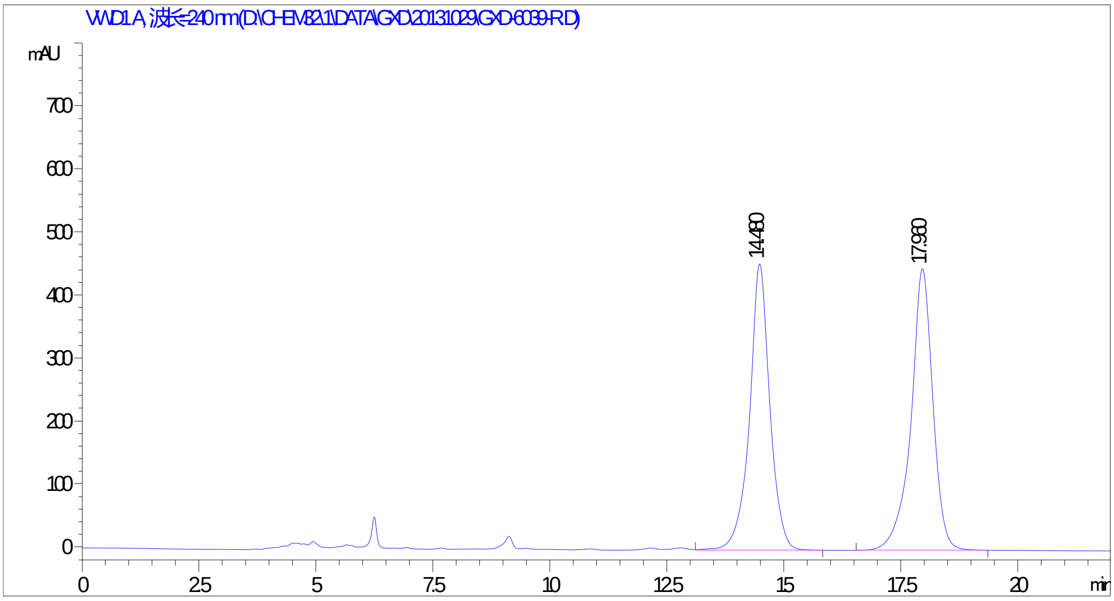

#	Time	Area	Height	Width	Symmetry	Area/%
1	14.48	13736.4	454.2	0.4402	0.987	48.513
2	17.96	14578.8	446.8	0.4797	1.092	51.487

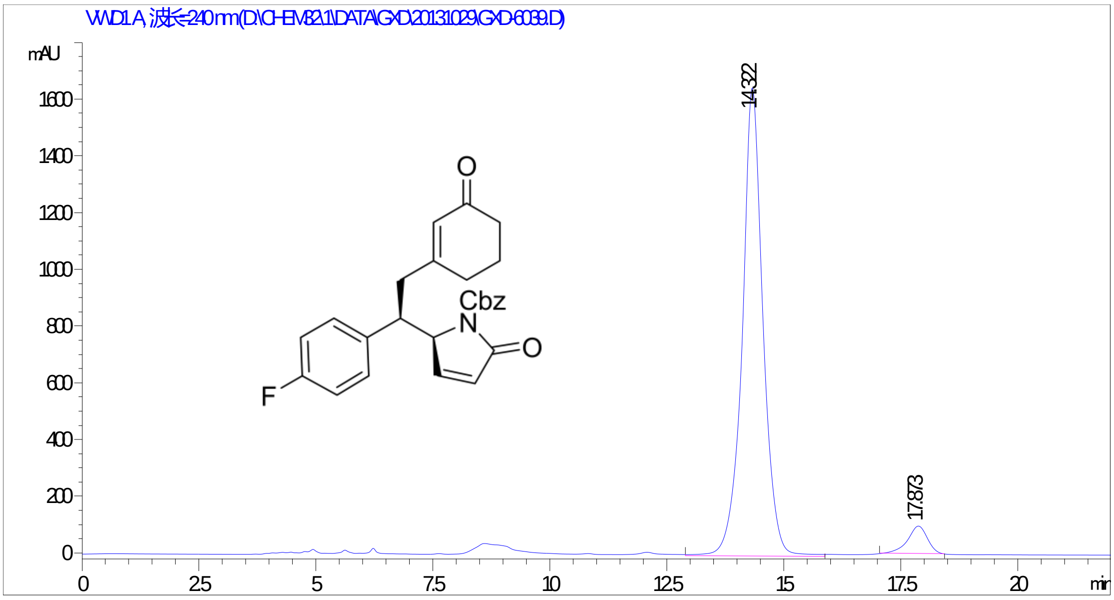

#	Time	Area	Height	Width	Symmetry	Area/%
1	14.322	51005.6	1650.9	0.5149	0.917	94.612
2	17.873	2904.5	97.7	0.4954	1.179	5.388

(*S*)-5-((*R*)-2-(3-oxocyclohex-1-enyl)-1-phenylethyl)-1-tosyl-1,5-dihydro-2*H*-pyrrol-2-one (4t)

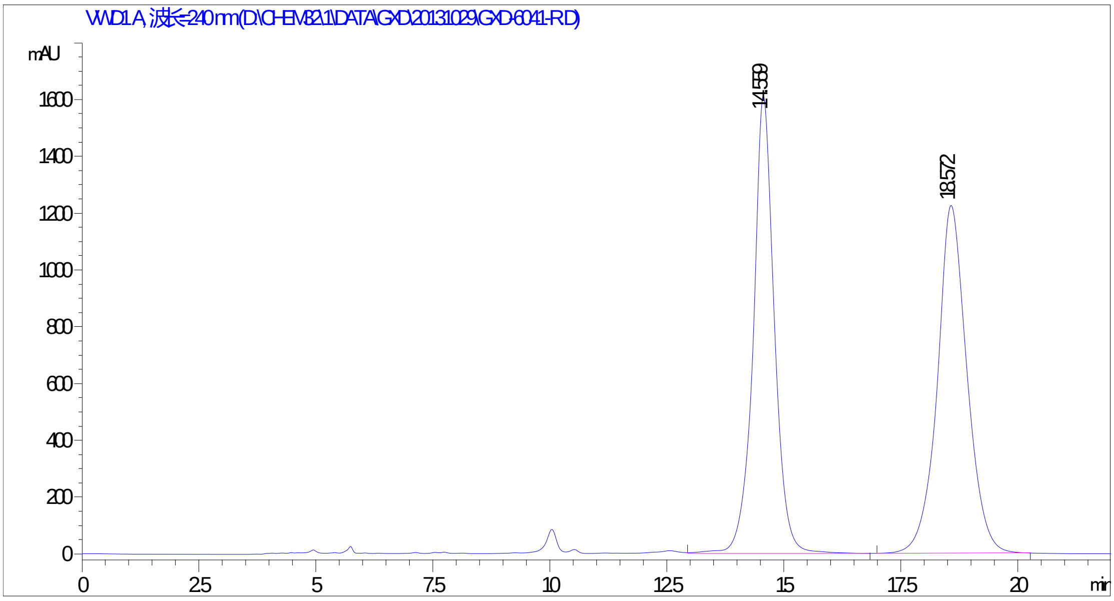

#	Time	Area	Height	Width	Symmetry	Area/%
1	14.559	50989.3	1612.8	0.4635	0.906	48.525
2	18.572	54089.7	1224.9	0.7359	0.858	51.475

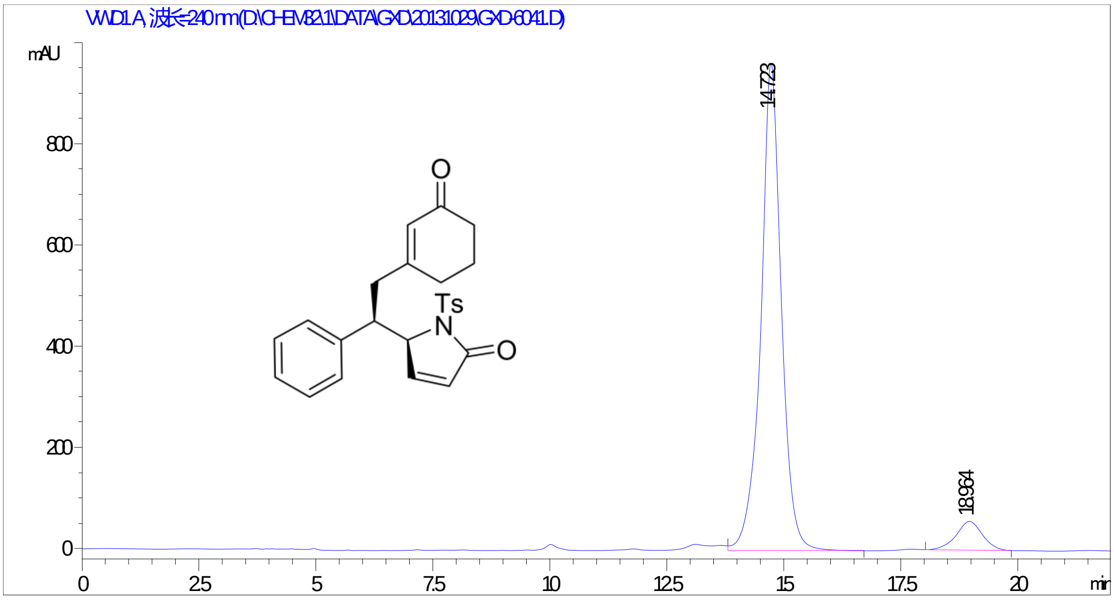

#	Time	Area	Height	Width	Symmetry	Area/%
1	14.723	29314.1	963.1	0.4454	0.958	92.737
2	18.964	2295.9	57.4	0.6663	1.02	7.263

(*S*)-5-((*R*)-1-(4-fluorophenyl)-2-(3-oxocyclohex-1-enyl)ethyl)-1-tosyl-1,5-dihydro-2*H*-pyrrol-2-one (4u)

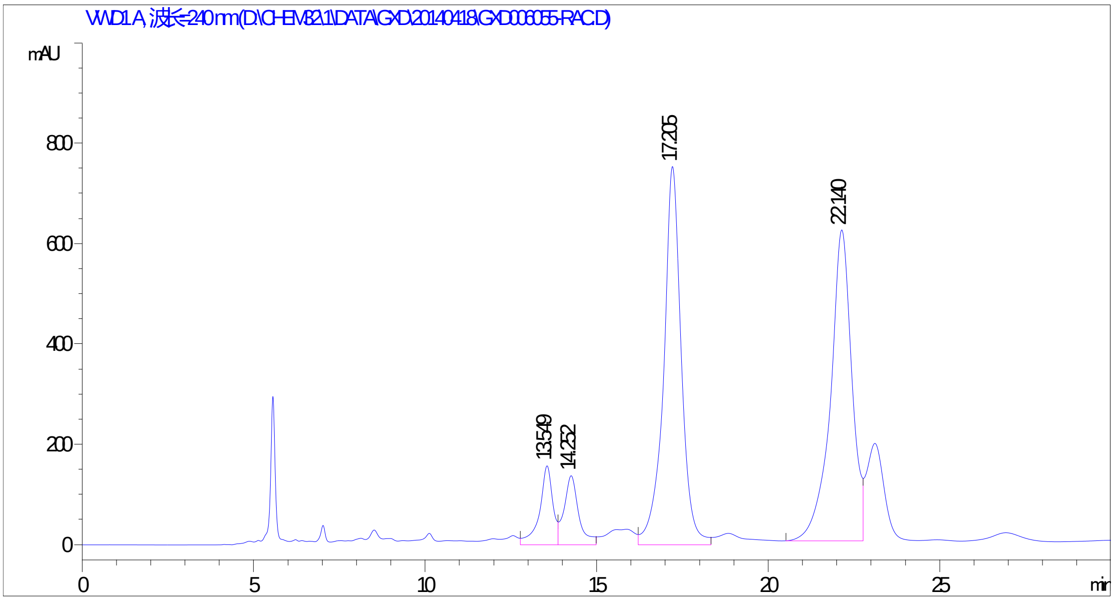

#	Time	Area	Height	Width	Symmetry	Area/%
1	13.549	4255.3	157.5	0.3866	1.332	6.822
2	14.252	4067.6	137.8	0.4259	0.85	6.521
3	17.205	27103.6	753.9	0.5242	0.993	43.454
4	22.14	26946.3	620.1	0.7243	1.114	43.202

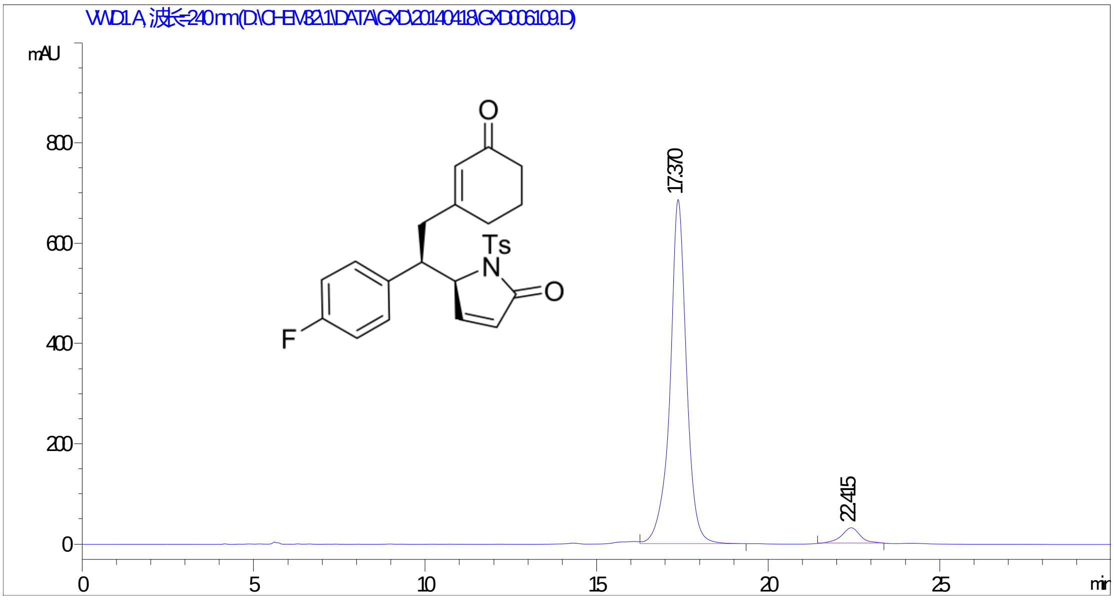

#	Time	Area	Height	Width	Symmetry	Area/%
1	17.37	22969	686.9	0.4949	0.963	94.851
2	22.415	1247	31.4	0.6627	1.056	5.149

(*S*)-5-((*R*)-1-(3,4-dimethoxyphenyl)-2-(3-oxocyclohex-1-enyl)ethyl)-1-tosyl-1,5-dihydro-2*H*-pyrrol-2-one (4v)

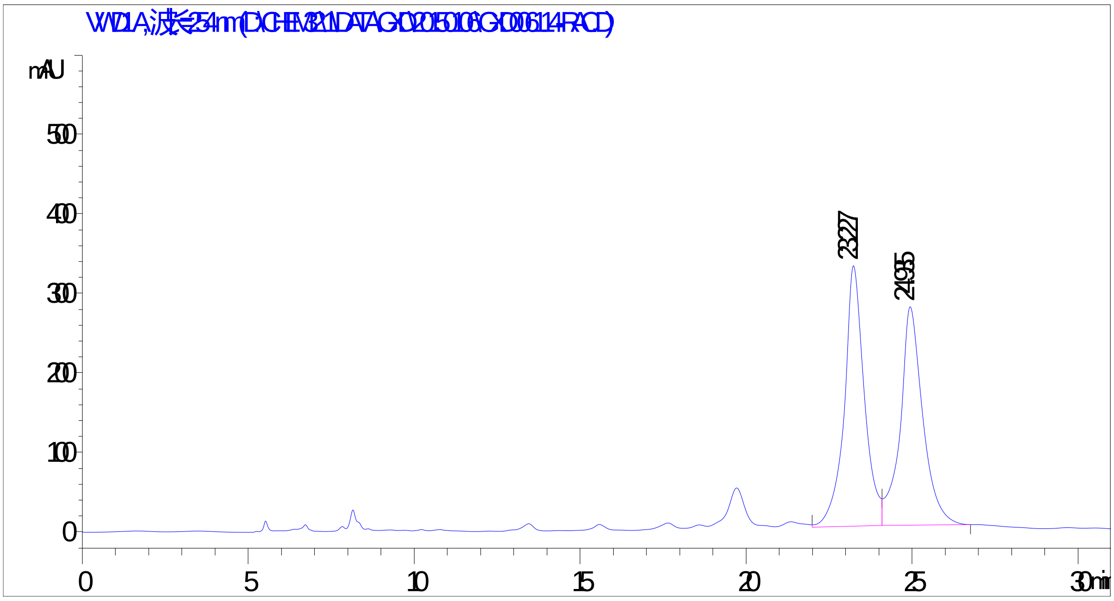

#	Time	Area	Height	Width	Symmetry	Area/%
1	23.227	13127.3	328.2	0.6666	0.744	50.063
2	24.935	13094.5	275.4	0.6814	0.781	49.937

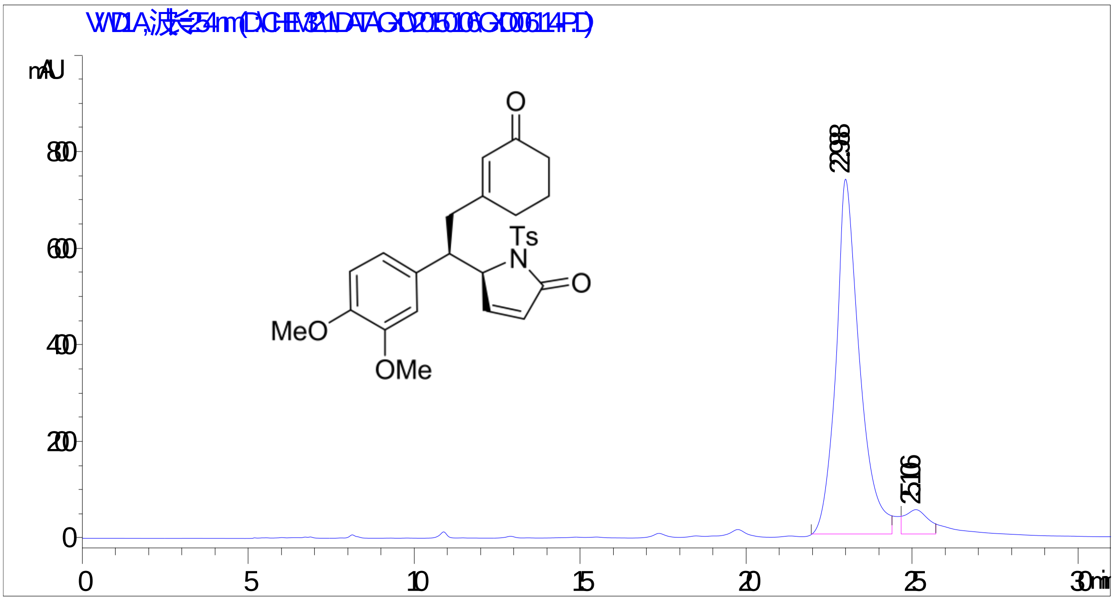

#	Time	Area	Height	Width	Symmetry	Area/%
1	22.988	36375	735.6	0.8241	0.671	95.547
2	25.106	1695.1	41.4	0.6825	0.998	4.453

***tert*-Butyl (3a*R*,4*R*,5a*S*,8b*S*)-2,7-dioxo-4-phenyl-1,3a,4,5,5a,6,7,8b-octahydrocyclo penta[*e*]indole-3(2*H*)-carboxylate (6a)**

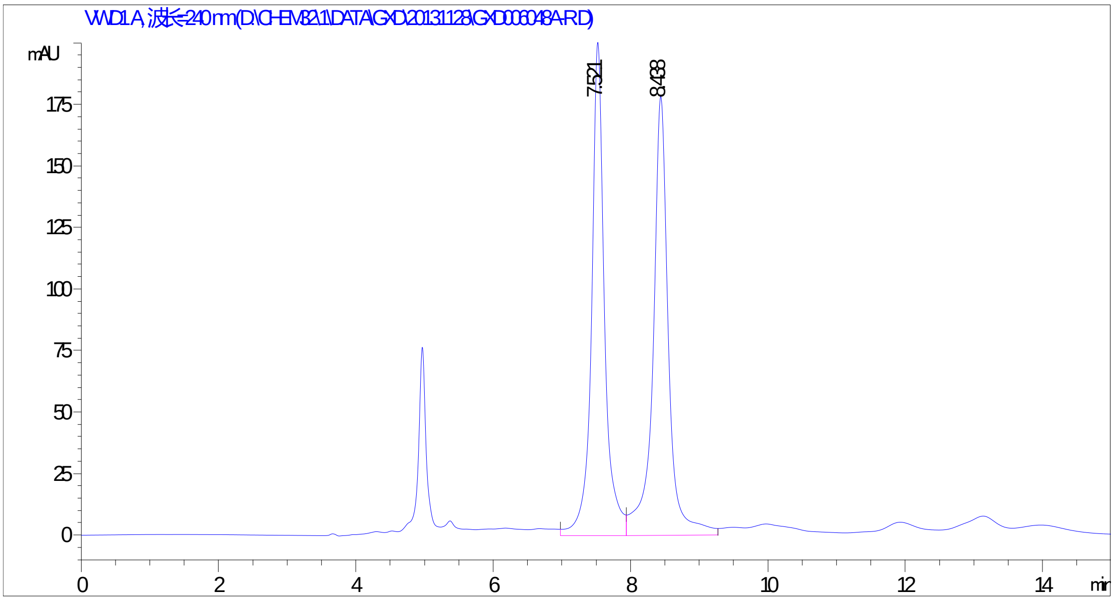

#	Time	Area	Height	Width	Symmetry	Area/%
1	7.521	2650.1	200.5	0.1921	0.906	48.622
2	8.438	2800.3	178.4	0.2282	0.966	51.378

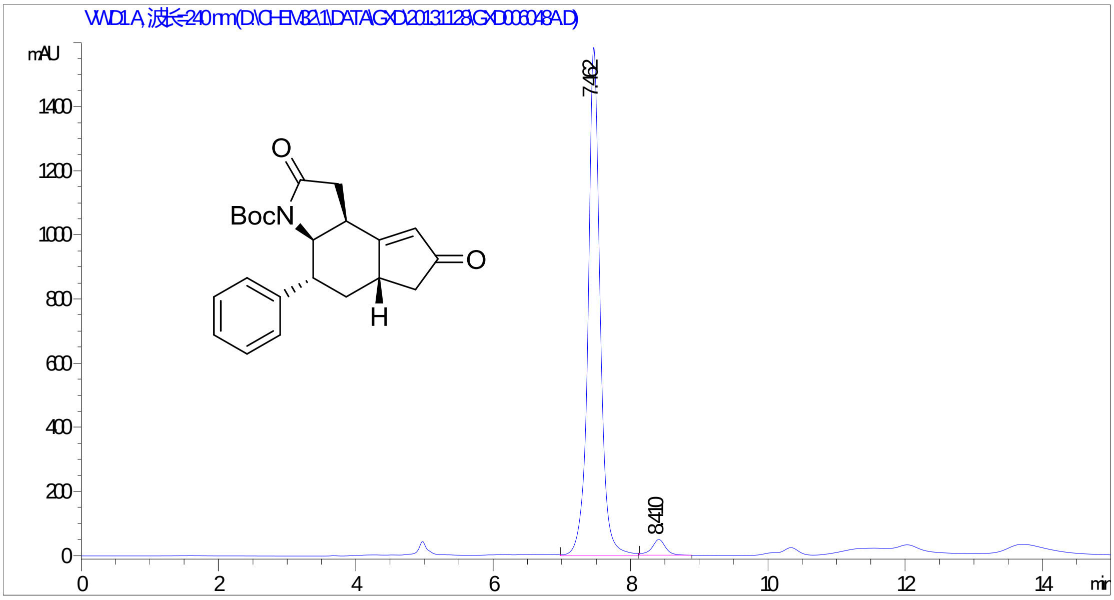

#	Time	Area	Height	Width	Symmetry	Area/%
1	7.462	19440.8	1586.6	0.1823	0.873	96.224
2	8.41	762.9	50.9	0.2497	0.973	3.776

***tert*-Butyl (3a*R*,4*R*,5a*S*,8b*S*)-2,7-dioxo-4-(*p*-tolyl)-1,3a,4,5,5a,6,7,8b-octahydrocyclopenta[*e*]indole-3(2*H*)-carboxylate (6b)**

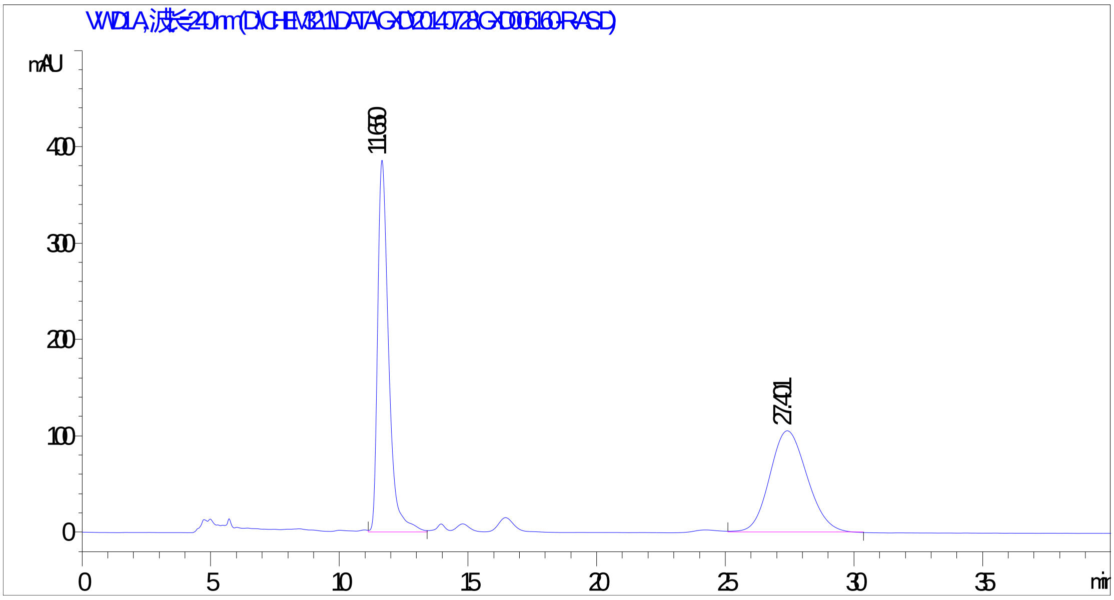

#	Time	Area	Height	Width	Symmetry	Area/%
1	11.65	11032.4	386	0.4764	0.66	50.532
2	27.401	10800.3	105.7	1.5593	0.852	49.468

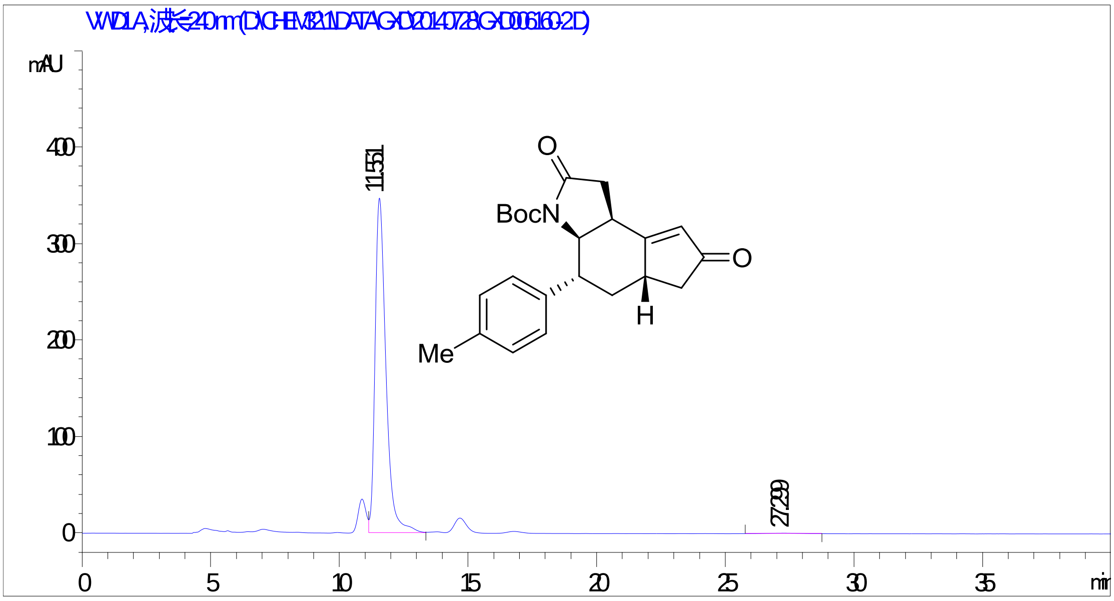

#	Time	Area	Height	Width	Symmetry	Area/%
1	11.551	10248.3	347.4	0.449	0.614	99.524
2	27.299	49.1	5.8E-1	1.421	1.076	0.476

***tert*-Butyl (3a*R*,4*R*,5a*S*,8b*S*)-4-(4-(*tert*-butyl)phenyl)-2,7-dioxo-1,3a,4,5,5a,6,7,8b-octahydrocyclopenta[*e*]indole-3(2*H*)-carboxylate (6c)**

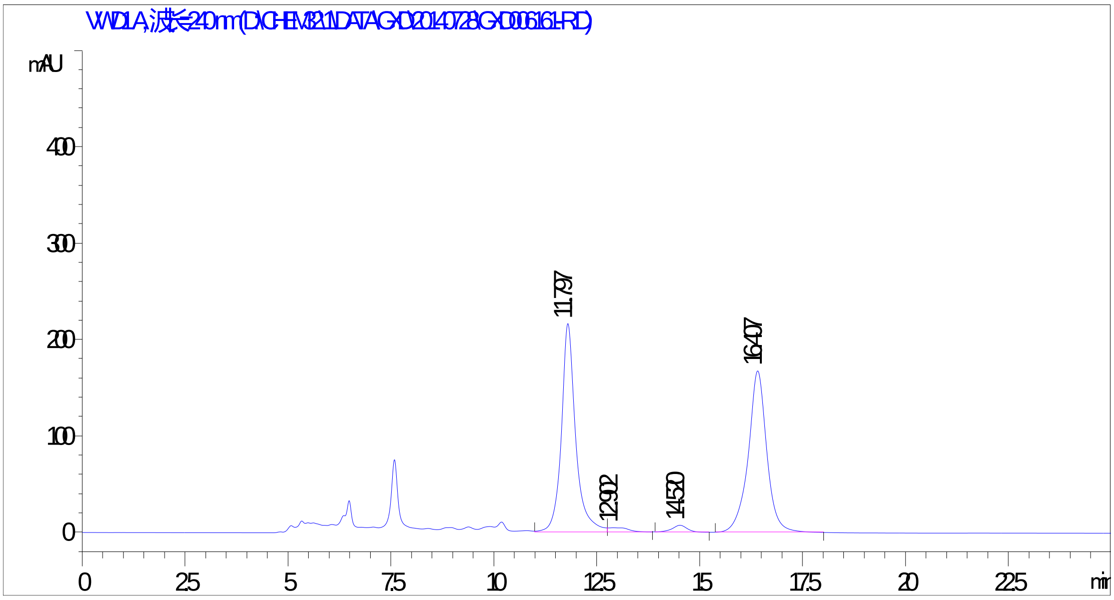

#	Time	Area	Height	Width	Symmetry	Area/%
1	11.797	5302	217	0.3521	0.791	48.129
2	12.902	192.6	5.1	0.4766	0.25	1.748
3	14.52	222.4	7.7	0.4213	1.079	2.019
4	16.407	5299.3	167.8	0.4616	1.01	48.104

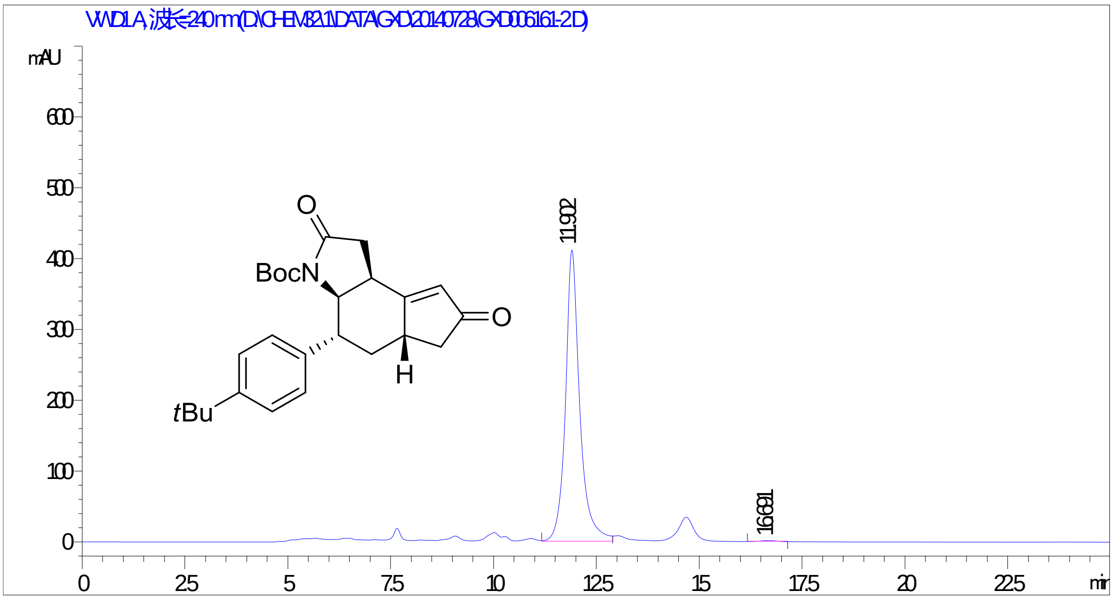

#	Time	Area	Height	Width	Symmetry	Area/%
1	11.902	10014.4	411.7	0.3507	0.769	99.574
2	16.691	42.9	1.5	0.4674	1.008	0.426

***tert*-Butyl (3a*R*,4*R*,5a*S*,8b*S*)-4-(naphthalen-2-yl)-2,7-dioxo-1,3a,4,5,5a,6,7,8b-octahydrocyclopenta[*e*]indole-3(2*H*)-carboxylate (6d)**

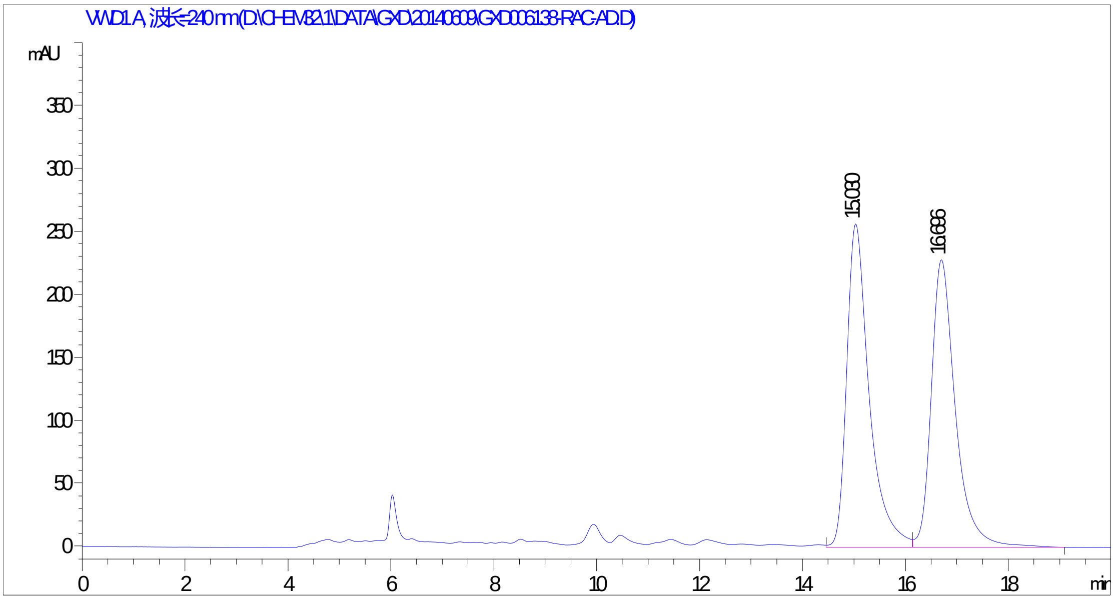

#	Time	Area	Height	Width	Symmetry	Area/%
1	15.03	7300.5	252.4	0.482	0.666	50.197
2	16.696	7243	226.8	0.5322	0.706	49.803

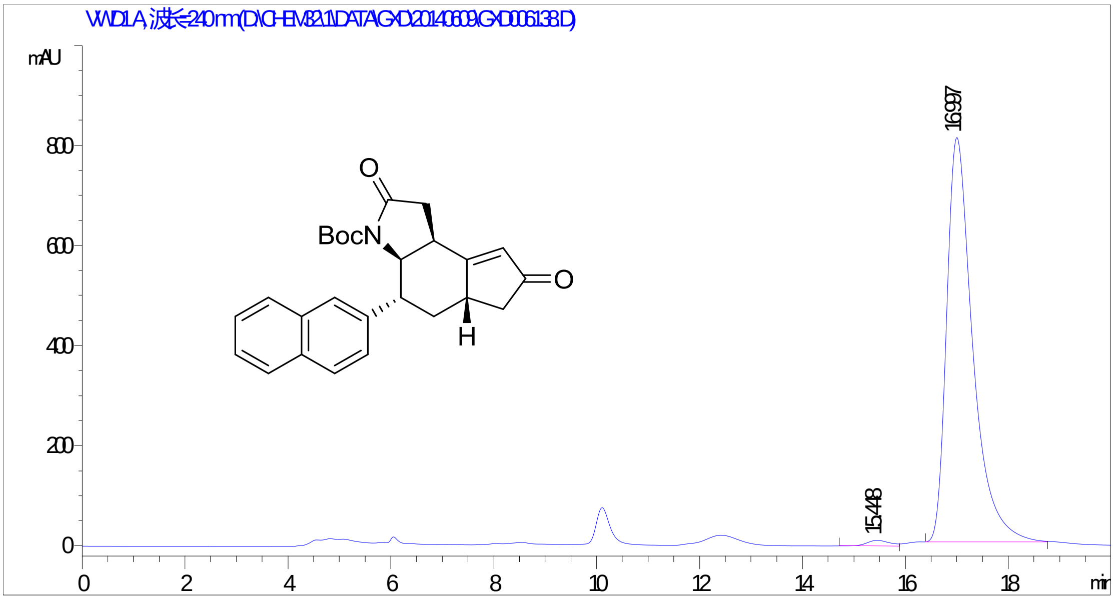

#	Time	Area	Height	Width	Symmetry	Area/%
1	15.448	370.9	11.6	0.5331	0.837	1.282
2	16.997	28565.4	808.3	0.544	0	98.718

***tert*-Butyl (3a*S*,4*R*,5a*S*,8b*S*)-2,7-dioxo-4-(thiophen-3-yl)-1,3a,4,5,5a,6,7,8b-octahydrocyclopenta[*e*]indole-3(2*H*)-carboxylate (6e)**

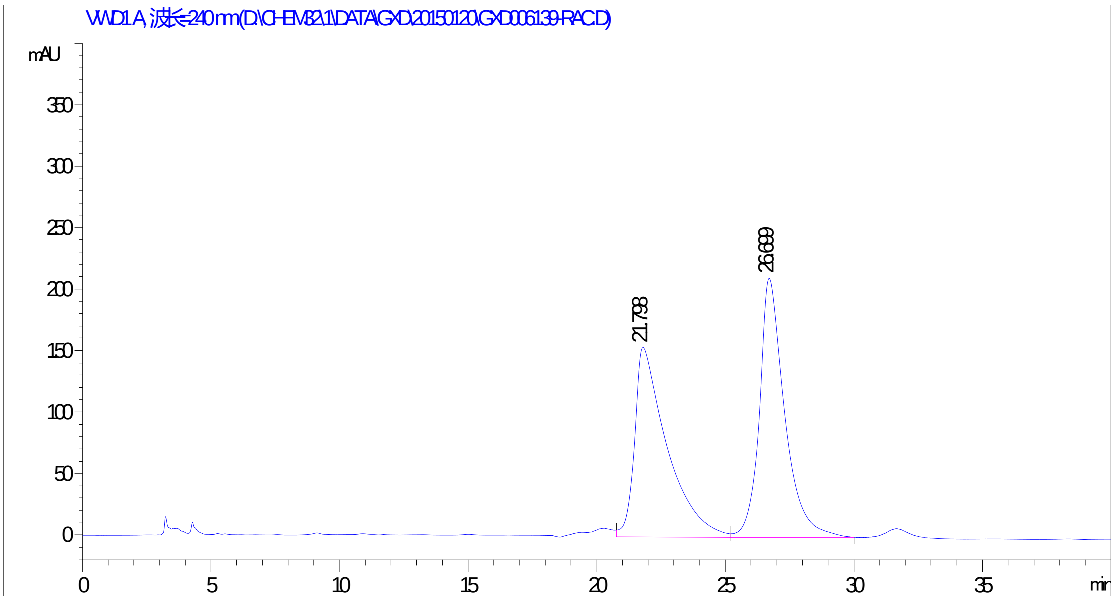

#	Time	Area	Height	Width	Symmetry	Area/%
1	21.798	13595.3	154.4	1.1742	0.345	49.781
2	26.699	13715	207.9	1.0997	0.623	50.219

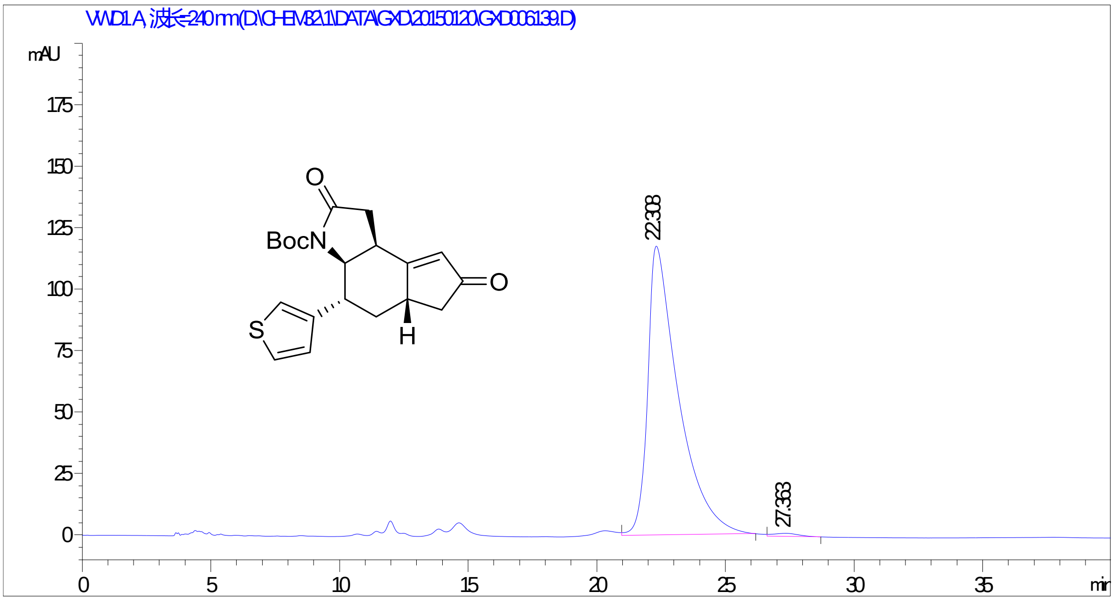

#	Time	Area	Height	Width	Symmetry	Area/%
1	22.308	9812.5	117.6	1.1656	0.372	98.881
2	27.363	111	1.5	1.2646	0.887	1.119

Benzyl (3a*R*,4*R*,5a*S*,8b*S*)-2,7-dioxo-4-phenyl-1,3a,4,5,5a,6,7,8b-octahydrocyclopenta[*e*]indole-3(2*H*)-carboxylate (6f)

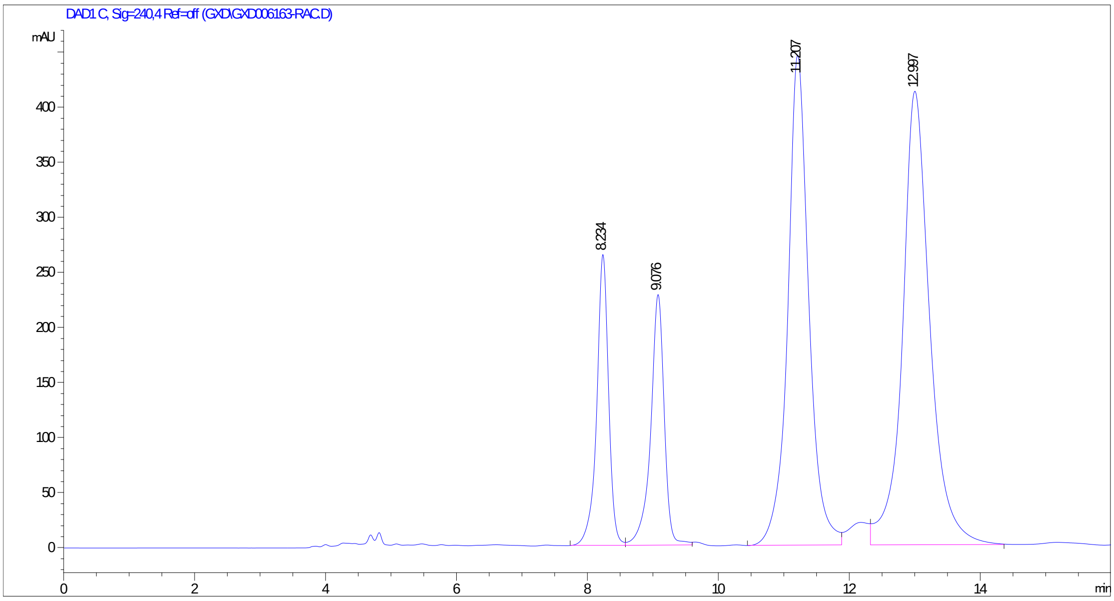

#	Time	Area	Height	Width	Symmetry	Area/%
1	8.234	3342.5	264.4	0.1875	1.08	12.575
2	9.076	3298.3	227.9	0.2148	1.177	12.409
3	11.207	9966.2	445.4	0.331	0.859	37.496
4	12.997	9972.5	389.1	0.4271	0.812	37.520

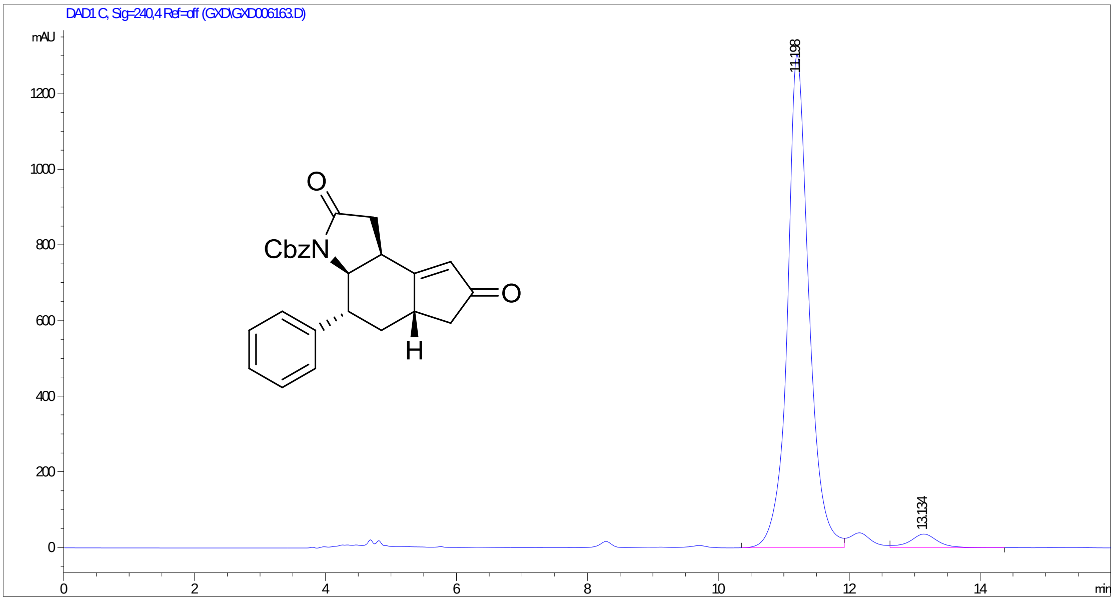

#	Time	Area	Height	Width	Symmetry	Area/%
1	11.198	29875.5	1304	0.3373	0.787	96.536
2	13.134	1071.9	36.1	0.4382	0.977	3.464
